# Putative ligand binding sites of two functionally characterized bark beetle odorant receptors

**DOI:** 10.1186/s12915-020-00946-6

**Published:** 2021-01-26

**Authors:** Jothi K. Yuvaraj, Rebecca E. Roberts, Yonathan Sonntag, Xiao-Qing Hou, Ewald Grosse-Wilde, Aleš Machara, Dan-Dan Zhang, Bill S. Hansson, Urban Johanson, Christer Löfstedt, Martin N. Andersson

**Affiliations:** 1 Department of Biology, Lund University, SE-223 62 Lund, Sweden; 2 Division of Biochemistry and Structural Biology, Department of Chemistry, Lund University, SE-223 62 Lund, Sweden; 3 Department of Evolutionary Neuroethology, Max Planck Institute for Chemical Ecology, 07745 Jena, Germany; 4 Present address: Faculty of Forestry & Wood Sci, Excellent Team for Mitigation, Czech University Life Sci Prague, Kamycka 129, Prague 6, 16521 Suchdol, Czech Republic; 5 Institute of Organic Chemistry and Biochemistry, Academy of Sciences of the Czech Republic, Gilead Sciences and IOCB Research Center, Flemingovo n. 2, 166 10 Prague 6, Czech Republic

**Keywords:** Deorphanization, Functional evolution, HEK293 cells, Odorant receptor, Pheromone receptor, Pest insect, *Xenopus* oocyte

## Abstract

**Background:**

Bark beetles are major pests of conifer forests, and their behavior is primarily mediated via olfaction. Targeting the odorant receptors (ORs) may thus provide avenues towards improved pest control. Such an approach requires information on the function of ORs and their interactions with ligands, which is also essential for understanding the functional evolution of these receptors. Hence, we aimed to identify a high-quality complement of ORs from the destructive spruce bark beetle *Ips typographus* (Coleoptera, Curculionidae, Scolytinae) and analyze their antennal expression and phylogenetic relationships with ORs from other beetles. Using 68 biologically relevant test compounds, we next aimed to functionally characterize ecologically important ORs, using two systems for heterologous expression. Our final aim was to gain insight into the ligand-OR interaction of the functionally characterized ORs, using a combination of computational and experimental methods.

**Results:**

We annotated 73 ORs from an antennal transcriptome of *I. typographus* and report the functional characterization of two ORs (ItypOR46 and ItypOR49), which are responsive to single enantiomers of the common bark beetle pheromone compounds ipsenol and ipsdienol, respectively. Their responses and antennal expression correlate with the specificities, localizations, and/or abundances of olfactory sensory neurons detecting these enantiomers. We use homology modeling and molecular docking to predict their binding sites. Our models reveal a likely binding cleft lined with residues that previously have been shown to affect the responses of insect ORs. Within this cleft, the active ligands are predicted to specifically interact with residues Tyr84 and Thr205 in ItypOR46. The suggested importance of these residues in the activation by ipsenol is experimentally supported through site-directed mutagenesis and functional testing, and hydrogen bonding appears key in pheromone binding.

**Conclusions:**

The emerging insight into ligand binding in the two characterized ItypORs has a general importance for our understanding of the molecular and functional evolution of the insect OR gene family. Due to the ecological importance of the characterized receptors and widespread use of ipsenol and ipsdienol in bark beetle chemical communication, these ORs should be evaluated for their potential use in pest control and biosensors to detect bark beetle infestations.

**Supplementary Information:**

The online version contains supplementary material available at 10.1186/s12915-020-00946-6.

## Background

Olfaction is of utmost importance for the fitness of insects. Detection of odors underlies successful host- and mate-finding, pathogen avoidance, and maintenance of symbioses with essential microbes [1, 2]. Neuronal responses that ultimately may induce an olfactory-guided behavior are triggered when odorants interact with odorant receptors (ORs), which are located in the dendrites of olfactory sensory neurons (OSNs) in the antennae [3]. Insect ORs, which are unrelated to G-protein coupled vertebrate ORs [4, 5], are encoded by a large gene family [6], undergoing a dynamic “birth-and-death” evolution. In this model, gene duplication represents the birth, and pseudogenization and deletion the death of genes [7, 8]. With some exceptions (reviewed in [7]), a single OR gene is expressed in each OSN together with the co-receptor Orco, which is conserved across insects, except in the most basal taxa [9]. Together, the OR and Orco are suggested to form a heterotetrameric receptor complex [10], with Orco being essential for the formation of an ion channel upon ligand-induced activation of the OR [11, 12]. Current knowledge of the functional evolution of the ORs as well as the ligand-OR interaction is however limited, yet crucial for understanding insect chemical ecology and species-specific sensory adaptations. From an applied perspective, functional characterization of ORs and determination of their binding sites are pertinent in pest insects, because receptors that are key to survival and reproduction represent potential targets for pest control using OR antagonists and agonists [13]. Also, with the advancement of biosensor technology towards using ORs to detect insect semiochemicals [14–16], employing receptors tuned to the characteristic odors of a pest could be useful for detection of infestations.

Conifer-feeding bark beetles (Coleoptera; Curculionidae; Scolytinae) pose serious threats to forestry, and bark beetle outbreaks are increasing due to climate change [17–19]. A warmer and drier climate reduces the defenses of the trees, at the same time as the beetles’ populations increase due to decreased generation time and winter mortality, and higher availability of breeding material resulting from severe weather events [18]. In light of the intensifying outbreaks, more efficient control and detection of bark beetles are needed, and one avenue forward may be to exploit the ORs [13] that are crucial for successful mate and host finding. The European spruce bark beetle (*Ips typographus* L.) is the most serious insect pest of Norway spruce (*Picea abies* (L.) H. Karst.) in large parts of Europe and Asia [20]. As other bark beetles, *I. typographus* is a keystone species in forest ecosystems, contributing to the decomposition of wood through direct feeding as well as through the spread of microorganisms, such as its associated fungi [2]. When beetle populations surpass a critical threshold, healthy trees are killed through mass attacks, and entire forest landscapes can be quickly transformed. Attacks on trees are coordinated via a male-produced aggregation pheromone. This pheromone is attractive to both sexes and is comprised of (−)*-cis*-verbenol and 2-methyl-3-buten-2-ol, with average quantities in male hindguts around 40 ng and 500 ng, respectively [21, 22]. These compounds act synergistically and are necessary to trigger maximal attraction [23]. Several other compounds are released by males during the later attack phases (2–6 days after initiation of boring when production of the aggregation pheromone declines; see ref. [22] for additional details), including verbenone, (*S*)*-*(−)-ipsenol, and (*R*)-(−)-ipsdienol [24]. The two former compounds reduce attraction of both sexes to the aggregation pheromone whereas effects of ipsdienol are less clear [25]. The produced quantities of these compounds are generally lower as compared to the aggregation pheromone components, with for instance ipsenol and ipsdienol reaching approximately 10 ng/beetle (although ipsdienol demonstrates a large variation between individuals) [22]. Most of these compounds are also used by other bark beetle species, frequently as aggregation pheromones or pheromone antagonists. Moreover, volatiles released from host trees [26], non-host plants [27], heterospecific bark beetles [28], and fungal symbionts [2] affect the behavior of *I. typographus*. Significant efforts to characterize OSN responses of *I. typographus* have been undertaken, with 23 strongly responding OSN classes reported, including neurons tuned to bark beetle pheromones, host and non-host volatiles, or fungal compounds [2, 29–33]. Among the characterized pheromone–responsive neurons are two OSN classes which are narrowly tuned to the aggregation pheromone components (−)-*cis*-verbenol and 2-methyl-3-buten-2-ol, respectively [29]. In addition, several studies have identified and characterized three different OSN classes that are highly specific for the single enantiomers (*S*)-(−)-ipsenol, (*R*)-(−)-ipsdienol, and (*S*)-(+)-ipsdienol, even when challenged with large odor panels or structurally similar analogs [2, 29, 30, 32, 33]. The (*S*)-(−)-ipsenol-specific OSN class is among the more abundant OSN classes on the antennae of this species, whereas the two ipsdienol-specific OSN classes are slightly less common, and no obvious differences in the relative abundances of these and other OSN classes have been observed between sexes [29, 30]. The comparatively large knowledge of physiologically active compounds and OSN classes makes this species a good model for pursuing functional characterization of ORs.

Functional characterization of insect ORs has been biased towards moths [34–36], flies, and mosquitos [37, 38]. In contrast, Coleoptera—arguably the largest order of the Metazoa—remains an understudied group with only five characterized ORs from a few species: the cerambycid *Megacyllene caryae* [39], the scarab *Holotrichia parallela* [40], and the curculionid *Rhynchophorus ferrugineus* [41]. The paucity of functional data is a bottleneck that severely limits our understanding of the molecular evolution of olfaction in this diverse taxon [42]. For *I. typographus*, a previous study reported 43 ORs (ItypORs) from an antennal transcriptome [43]. The majority of these were, however, only reported as partial genes, and the essential Orco was not identified. Here, we aimed (*i*) to identify a high-quality complement of ORs in *I. typographus*, allowing for improved evolutionary analysis and functional characterization of ecologically important receptors (aim *ii*). Using two systems for heterologous expression and a panel of 68 test compounds, we report the characterization of two ORs (ItypOR46 and ItypOR49), which are narrowly tuned to single enantiomers of the common bark beetle pheromones ipsenol and ipsdienol, respectively, similar to previously characterized OSN classes for these compounds [29, 30]. The third aim (*iii*) was to gain insight into the mechanisms of ligand binding in these ORs that detect structurally similar pheromone compounds. Thus, we took advantage of the recently published cryo-EM structure of Orco [10] to perform homology modeling and ligand docking simulations. This analysis predicted a primarily hydrophobic cavity lined by residues that are likely to interact with the ligands. The predicted functional importance of two residues was supported experimentally using site-directed mutagenesis followed by functional testing. The deorphanization of the two ItypORs and prediction of their ligand binding sites provide new insight into the interaction between insect ORs and their ligands, which is important for understanding the molecular and functional evolution of the divergent insect OR family. This information could guide screenings for more potent OR agonists or antagonists to be used in control of *I. typograph*us. Future applications may also involve the use of these ORs in biosensors to detect a large number of bark beetle pests due to the widespread use of ipsenol and ipsdienol in bark beetle chemical ecology.

## Results

### OR annotation, expression levels, and phylogenetic analysis

To obtain an improved set of ItypOR sequences and to identify the Orco necessary for functional characterization in heterologous in vitro systems, we sequenced, assembled, and annotated an antennal transcriptome of *I. typographus*. The annotation revealed 73 ItypORs (including ItypOrco) of which 52 ORs corresponded to full-length proteins. Five of the original 43 ORs [43] were discarded as previous assembly isoforms. Hence, 35 ORs were novel sequences. The majority of the previously partial OR sequences were extended to full-length, and sequences that contained, e.g., previously unnoticed frameshifts or 5′/3′ intron sequence were corrected (Supplementary Table 1, Additional file 1). The currently still partial ItypOR sequences encode proteins comprising 76 to 377 amino acids. Molecular cloning from cDNA followed by sequence verification allowed us to combine short partial OR sequences encoded by eight transcripts into four unique longer, but still partial, genes (ItypOR57NTE, ItypOR61NTE, ItypOR70FN, and ItypOR71NTE; gene suffixes are explained in the “Methods” section).

The sequenced reads were mapped to the annotated OR gene sequences to estimate their expression levels (in transcripts per million, TPM) in the antennal transcriptome. As expected, the ItypOrco gene showed the highest expression (Supplementary Table 1, Additional file 1). Among the canonical OR genes, the functionally characterized ItypOR46 was the third most highly expressed receptor gene, whereas the expression of ItypOR49 was lower (approx. 1/3 of ItypOR46 and ranked #16 among the OR genes).

**Fig. 1:**
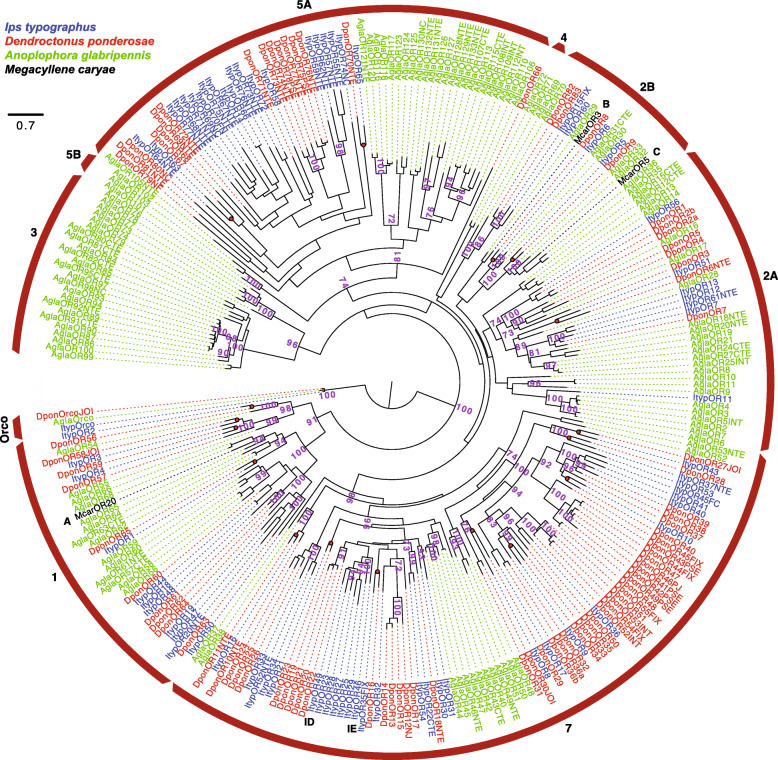
Maximum likelihood tree of beetle odorant receptors (ORs). The tree is based on a MAFFT alignment of amino acid sequences, constructed using RaXML, and rooted with the Orco lineage. Included are ORs from *Ips typographus* (Ityp; blue), *Dendroctonus ponderosae* (Dpon; red), *Anoplophora glabripennis* (Agla; green), and the three functionally characterized ORs from *Megacyllene caryae* (Mcar; black). Major coleopteran OR clades are indicated by the red arcs and numbered according to [44]. Note that OR group 6 is missing in the tree, since this lineage has been lost in species of the Cerambycidae and Curculionidae families. Numbers at nodes represent bootstrap support (*n* = 100), calculated using RaXML, and are only shown for main lineages and if ≥ 70. Red circles at nodes indicate the seventeen highly supported (bootstrap ≥  90) simple ItypOR/DponOR orthologs. Key ligands for functionally characterized ORs indicated in the tree (data for McarORs from [39]): A = (2*S*,3*R*)-2,3-hexanediol (McarOR20); B = (*S*)-2-methylbutan-1-ol (McarOR3); C = 2-phenylethanol (McarOR5); IE = (*S*)*-*(−)-ipsenol (ItypOR46); ID = (*R*)*-*(−)-ipsdienol (ItypOR49). The sources of sequence data and explanation of receptor suffixes are detailed in the “Methods” section

A recent study defined nine major clades of coleopteran ORs [44]. Our phylogenetic analysis of ORs from the Curculionidae and Cerambycidae families showed that the largest number of ItypORs were found in group 7 (29 ORs), followed by group 5A (21 ORs), group 1 (11 ORs), group 2A (7 ORs), and group 2B (4 ORs), which is similar to the OR distribution in the other bark beetle species in the analysis, *Dendroctonus ponderosae* (Fig. 1). The largest lineage expansions of ItypORs were present in group 5A (ten ORs, but with low support) and group 7 (seven ORs with 100% support, including ItypOR46 and ItypOR49). Additionally, *I. typographus* lacked ORs in groups 3, 4, 5B, and 6, which is also true for *D. ponderosae*. In contrast to previous studies [44, 45], our tree did not recapitulate the monophyly of OR group 2, with the two group 4 OR members from the cerambycid *Anoplophora glabripennis* being associated with the 2B group. This discrepancy is likely explained by the limited number of group 4 ORs in the analysis, in combination with the comparatively low node support for the 2A/2B distinction observed previously [44]. Our analysis also indicated the presence of 19 simple 1:1 orthologous relationships between ItypORs and DponORs of which 17 pairs have high bootstrap support (≥ 90%; Fig. 1). Apart from Orco, no simple orthologous relationships were observed between *I. typographus* and *A. glabripennis.*

### Functional characterization of ItypOrco, ItypOR46, and ItypOR49

**Fig. 2:**
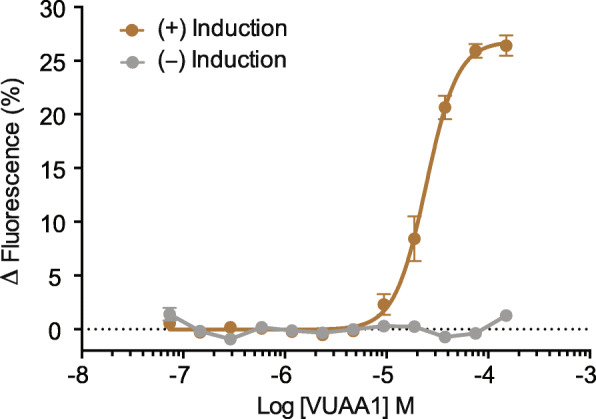
Dose-dependent response to the Orco agonist VUAA1 in TREx/HEK293 cells expressing only ItypOrco. Data represent mean responses ± SEM (*n* = 3 biological replicates, each including 3 technical replicates, i.e., *n*_total_ = 9). EC_50_ of VUAA1 = 24.46 μM. (+)-Induction: response of cells induced to express ItypOrco; (−)-Induction: response of non-induced control cells

ItypOrco and ORs were transfected into inducible TREx/HEK293 cells for functional characterization. Cells stably expressing ItypOrco responded dose-dependently to the Orco agonist VUAA1 (Fig. 2), representing the first example of VUAA1 responses in a beetle Orco protein. This cell line was transfected with each of ItypOR46 and ItypOR49. These two ORs share 43.4% amino acid identity and are part of the same radiation within OR group 7, and hence evolutionary unrelated to the characterized pheromone receptors of *M. caryae* (Fig. 1). The stably expressing ItypOrco/ItypOR46 and ItypOrco/ItypOR49 cell lines were analyzed by Western blot, which showed protein expression of myc-tagged ItypOrco and each of the two V5-tagged ItypORs. Proteins were detected in cells induced to express the exogenous Orco and OR genes, and not in the non-induced control cells, demonstrating proper regulation by the repression system (Supplementary Figure 1, Additional file 2). The *I. typographus*-specific OR radiation that encompasses ItypOR46 and ItypOR49 contains five additional ItypORs (ItypOR23, 25, 27, 28, and 29; Fig. 1). Because this is the largest species-specific OR clade with high bootstrap support and only full-length ORs, our initial aim was to functionally characterize all ORs in this clade. However, the five abovementioned ORs did not express in HEK cells. We therefore dropped these ORs from the present study and focused on the in-depth investigation of ItypOR46 and ItypOR49.

**Fig. 3:**
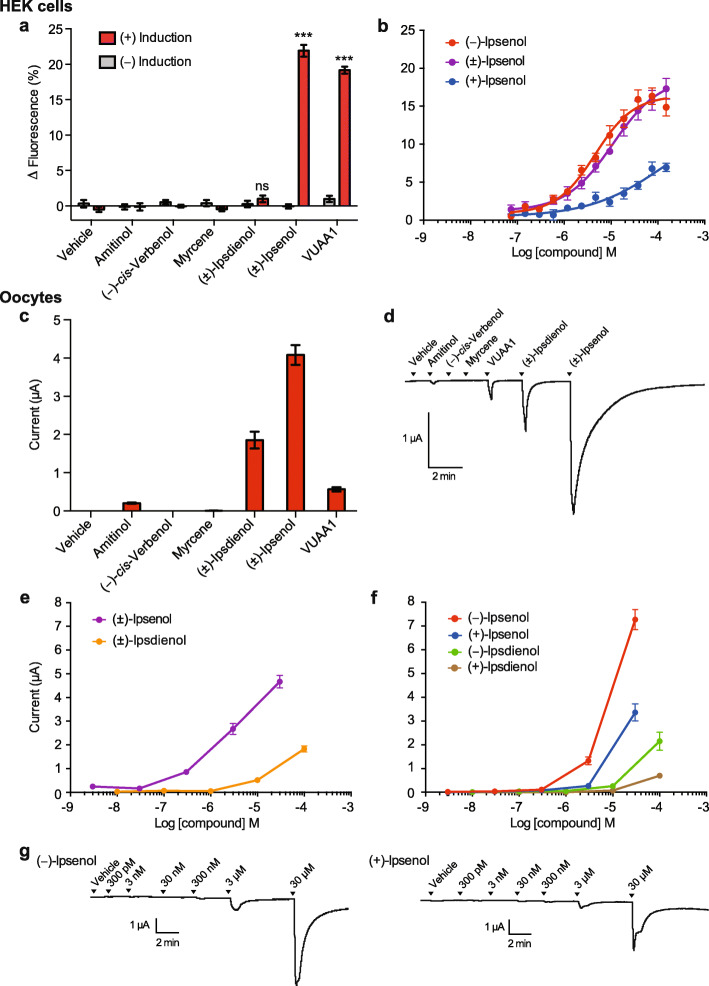
Functional characterization of ItypOR46. **a** Response of TREx/HEK293 cells expressing ItypOR46 and ItypOrco to select stimuli (30 μM) and vehicle control in the screening experiment (responses to all 68 odor stimuli are shown in Supplementary Fig. 2, Additional file 2). (+)-Induction: response of cells induced to express ItypOrco and ItypOR46; (−)-Induction: response of non-induced control cells. Asterisks (***) indicate a significant difference (at *p* < 0.001) between induced and non-induced cells; ns = not significant (*n* = 3 biological replicates, each including 3 technical replicates, i.e., *n*_total_ = 9). **b** Dose-dependent responses of the same TREx/HEK293 cell line to the pure enantiomers of ipsenol and the racemate (*n* = 4 biological replicates, each including 3 technical replicates, i.e., *n*_total_ = 12). EC_50_ values: (*S*)*-*(−)-ipsenol 1.98 μM; (±)-ipsenol 9.06 μM. **c** Current responses of *Xenopus* oocytes expressing ItypOR46 and ItypOrco in the screening experiment to the same stimuli (30 μM) as shown in **a** (*n* = 5). **d** Example of current trace responses from an oocyte expressing ItypOR46 and ItypOrco in the screening experiment (30 μM stimulus concentration). **e** Dose-dependent current response of oocytes expressing ItypOR46 and ItypOrco to racemic ipsenol (*n* = 6) and racemic ipsdienol (*n* = 5). **f** Dose-dependent current response of oocytes expressing ItypOR46 and ItypOrco to pure enantiomers of ipsenol (*n* = 4–5) and ipsdienol (*n* = 4). **g** Examples of current trace responses from oocytes expressing ItypOR46 and ItypOrco in dose-response experiments with (*S*)-(−)-ipsenol (*left* trace) and (*R*)-(+)-ipsenol (*right* trace). Data represent mean responses ± SEM (panels **a**–**c**, **e**–**f**)

Cells expressing ItypOrco/ItypOR46 and ItypOrco/ItypOR49 were screened for responses in a plate reader-based calcium fluorescence assay [46] against a panel of 68 ecologically relevant compounds (Supplementary Table 2, Additional file 2) at 30 μM concentration. In this experiment, ItypOR46 responded specifically to the pheromone compound (±)-ipsenol, with responses only recorded from cells induced to express ItypOrco and ItypOR46 (General Linear Model: F_1,14_ = 786; *p* < 0.001; Fig. 3a; Supplementary Figure 2, Additional file 2). A tendency for a secondary response to racemic ipsdienol was observed, but the response in the induced cells was not higher than that in non-induced cells (F_1,14_ = 1.17; *p* = 0.297). Dose-response trials with racemic ipsenol and its two pure enantiomers, which were synthesized in the present study (Supplementary Methods, Additional file 2), showed that ItypOR46 is highly specific for the natural enantiomer (*S*)*-*(−)-ipsenol, with responses elicited by (*R*)-(+)-ipsenol only occurring at higher concentrations (Fig. 3b). The response to (*R*)-(+)-ipsenol was likely due to the small percentage of the (*S*)*-*(−)-enantiomer present in the (*R*)-(+)-stimulus (Supplementary Table 2, Additional file 2).

Because insect OR responses sometimes depend on the system used for functional characterization [47], the ORs were also tested in *Xenopus* oocytes using a reduced odor panel of six compounds (30 μM). Ipsenol was still the most active ligand for ItypOR46, but ipsdienol elicited a secondary response in this system (Fig. 3c, d). A minor response was elicited also by the structurally similar compound amitinol, but this response could well be attributed to the presence of ipsdienol (3%) in the stimulus, an impurity which was identified by gas chromatography-mass spectrometry (GC-MS). The dose-response trials in the oocyte system indicated a higher sensitivity of ItypOR46 towards racemic ipsenol compared to racemic ipsdienol (Fig. 3e). Additional dose-response experiments testing pure enantiomers of ipsenol and ipsdienol confirmed that (*S*)-(−)-ipsenol is the primary ligand for this OR, although the enantio-specificity appeared lower in oocytes compared to HEK cells (Fig. 3f, g). These experiments also showed that the secondary response of ItypOR46 to racemic ipsdienol is mainly triggered by the (*R*)-(−)-enantiomer (Fig. 3f).

To investigate whether the absence of protein tags in oocytes was responsible for the different response specificity as compared to that in HEK cells (where both ItypOrco and ItypOR46 were tagged), we expressed myc-tagged ItypOrco together with V5-tagged ItypOR46 in oocytes. The secondary response of ItypOR46 to racemic ipsdienol and the minor response to amitinol remained, demonstrating that the tags did not underlie the system-dependent response specificity of ItypOR46 (Supplementary Figure 4, Additional file 2).

**Fig. 4:**
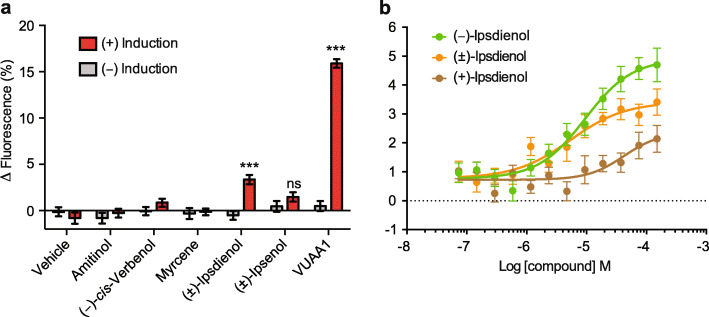
Functional characterization of ItypOR49. **a** Response of TREx/HEK293 cells expressing ItypOR49 and ItypOrco to select stimuli (30 μM) and vehicle control in the screening experiment (responses to all 68 odor stimuli are shown in Supplementary Fig. 3, Additional file 2). (+)-Induction: response of cells induced to express ItypOrco and ItypOR49; (−)-Induction: response of non-induced control cells. Asterisks (***) indicate a significant difference (at *p* < 0.001) between induced and non-induced cells; ns = not significant (*n* = 3 biological replicates, each including 3 technical replicates, i.e., *n*_total_ = 9). **b** Dose-dependent responses of the same TREx/HEK293 cell line to the pure enantiomers of ipsdienol and the racemate (*n* = 6 biological replicates, each including 3 technical replicates, i.e., *n*_total_ = 18). EC_50_ values: (*R*)*-*(−)-ipsdienol 9.47 μM; (±)-ipsdienol 5.34 μM. Data represent mean responses ± SEM

HEK cells expressing ItypOrco/ItypOR49 responded only to racemic ipsdienol in the screening experiment (F_1,14_ = 28.02; *p* < 0.001; Fig. 4a, Supplementary Figure 3, Additional file 2), although the response was smaller compared to the response of cells expressing ItypOrco/ItypOR46 to ipsenol. A tendency for a secondary response to racemic ipsenol was observed, but it was not higher in induced compared to non-induced cells (F_1,14_ = 1.61; *p* = 0.225). Dose-response experiments that included racemic ipsdienol and its two pure enantiomers (synthesized in the present study; Supplementary Methods, Additional file 2 [48–52]) showed that ItypOR49 is specifically tuned to (*R*)-(−)-ipsdienol, with responses to the (*S*)-(+)-enantiomer occurring only at higher stimulus concentrations. As with ItypOR46, these responses were likely due to the low percentage of (*R*)-(−)-ipsdienol in the (*S*)-(+)-enantiomer stimulus (Supplementary Table 2, Additional file 2). ItypOR49 was generally non-responsive in the oocytes, apart from minute ipsdienol-induced changes in current (approx. 5 nA) in occasional oocytes (Supplementary Figure 4, Additional file 2).

As expected, significant responses to the Orco agonist VUAA1 were recorded from HEK cells expressing ItypOrco/ItypOR46 (F_1,14_ = 792; *p* < 0.001; Fig. 3a) and ItypOrco/ItypOR49 (F_1,14_ = 469; *p* < 0.001; Fig. 4a), demonstrating functional Orco expression. VUAA1 responses were also recorded from oocytes co-injected with ItypOrco and each of the two ORs (Fig. 3c, d; Supplementary Figure 4, Additional file 2). In HEK cells expressing ItypOrco/ItypOR46, the VUAA1 response magnitude at the 30 μM concentration was similar to the response elicited by racemic ipsenol (Fig. 3a), whereas the VUAA1 response of Orco in the oocytes was 7-fold lower than the response to ipsenol (Fig. 3c). This variation may be due to differences in the relative expression of the OR versus Orco in the different systems, or system-dependent effects on the assembly of the receptor complex, affecting the accessibility of the binding site for VUAA1.

### Protein modeling and molecular docking

**Fig. 5:**
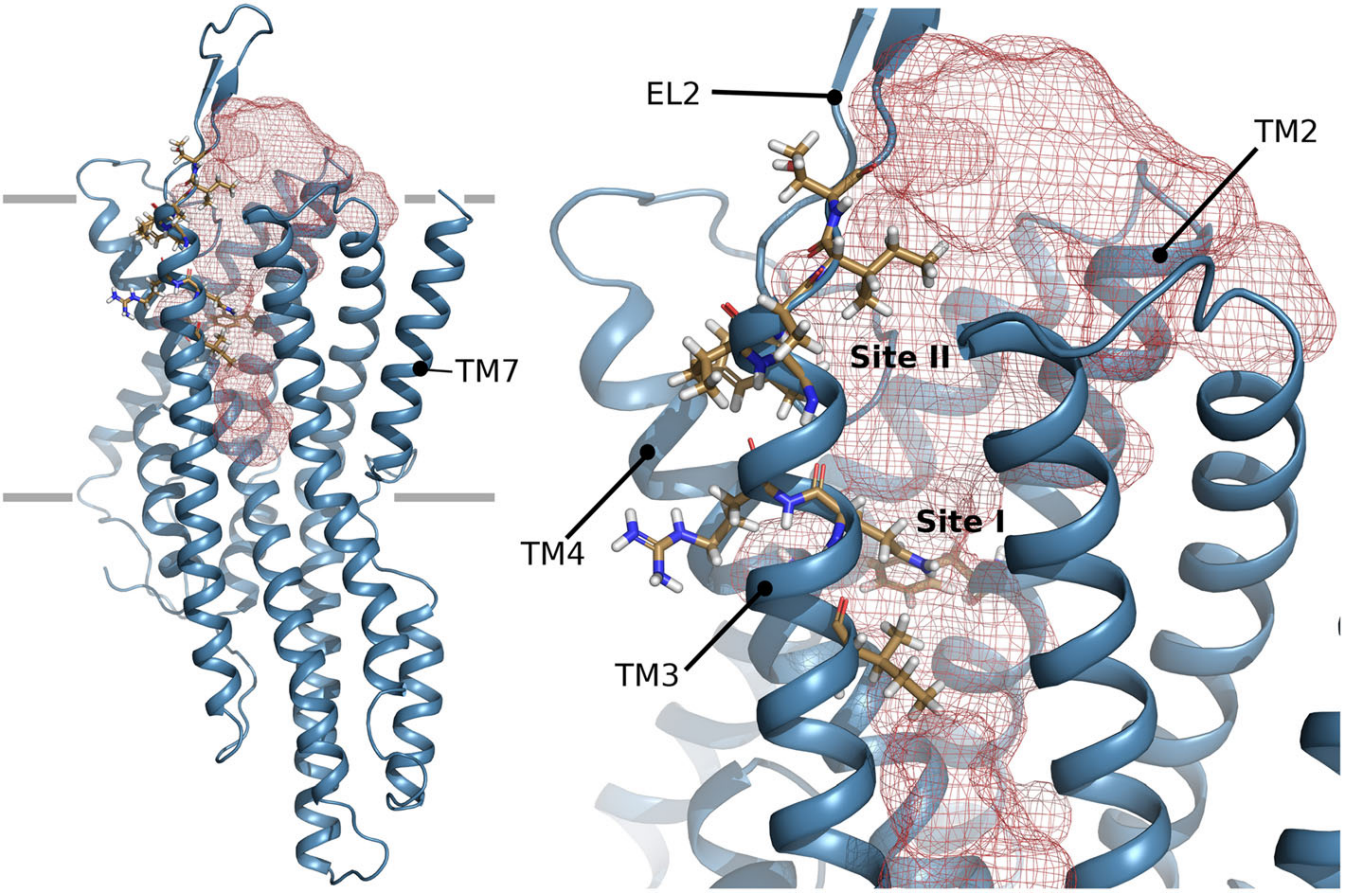
Protein model of ItypOR49. *Left image:* overview model indicating the predicted binding cavity in red mesh. Residues lining this cavity which in previous studies have been shown to affect ligand binding are shown in stick representation. Transmembrane (TM) helix 7 that forms the ion channel in the tetrameric Orco complex is located to the right. The expected extension of the lipid bilayer is indicated by gray lines. *Right image:* closer view of the cavity in the transmembrane region. Predicted binding site I and II as well as TM domains 2–4 and extracellular loop (EL) 2 are indicated

To gain insight into the ligand binding mechanisms of ItypOR46 and ItypOR49, protein homology modeling and molecular docking simulations were performed. First, an alignment of the two ItypORs with a total of 3185 ORs and Orco sequences [53, 54] was generated (Supplementary Data 1, Additional file 3). Key residues with high conservation among ORs and Orco proteins were used to assess correct alignment and threading of the modeled ORs. The models of ItypOR46 and ItypOR49 confirmed the presence of an extracellular cavity observed in the Orco structure and suggested to form a binding cleft [10]. The part of extracellular loop 2 not included in the published Orco structure (residues 156–170) was modeled ab initio, and the extended fold leaving the binding cleft exposed was supported by the cryo-EM density map of the Orco structure [10] (Supplementary Figure 5, Additional file 2). Several residues that have been implied to affect ligand specificity in various ORs (reviewed in [54]) line this cleft (Fig. 5), and significant differences between ItypOR46 and ItypOR49 were observed (described below), which may account for their dissimilarities in ligand specificity. Residues that when mutated were shown to affect inhibition of odor detection of OR59b by DEET in *Drosophila melanogaster* (“Dmel”) are located on transmembrane helix 2 [55], and residues affecting 2-heptanone specificity of DmelOR85b and pheromone response of OR3 in *Ostrinia* moths [56, 57] are located on transmembrane helix 3, extra cellular loop 2, and transmembrane helix 4. The locations of these residues indicate that they possibly affect ligand binding (Fig. 5), both at the extracellular entrance and at the deep end of the cleft, approximately at the center of the transmembrane domain. Hence, the docking site was defined to explore OR-ligand interaction possibilities throughout this confined region.

Molecular docking simulations included both enantiomers of ipsenol and ipsdienol, as well as the structurally similar but inactive compounds amitinol and myrcene, and the unrelated compound 1-hexanol (Supplementary Figure 6, Additional file 2). Ipsenol and ipsdienol docked to two distinct locations in ItypOR46 and ItypOR49, respectively, directed by OR-specific residues that line the cleft. One deep site (site I) is located where mutational studies identified residues important for inhibition of odor detection of *Drosophila* OR59b by DEET [55], and one site is closer to the extracellular opening of the cleft (site II; Fig. 5). Most of the residues that have been shown to affect OR responses are concentrated at these two sites, or in their vicinity (reviewed in [54]).

**Fig. 6:**
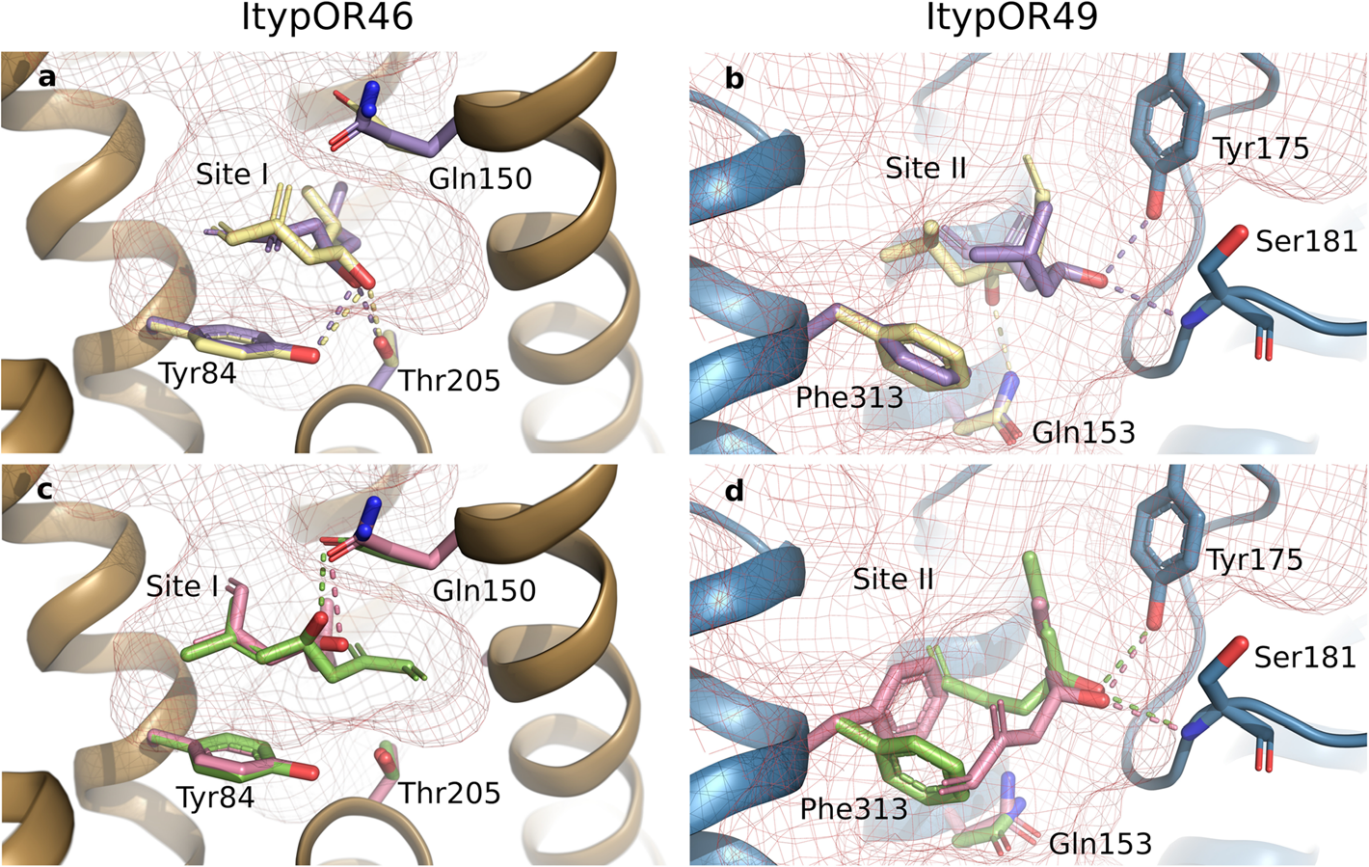
Results from molecular docking analysis. Predicted binding of (*R*)-(+)-ipsenol (purple) and (*S*)-(−)-ipsenol (yellow) to **a** ItypOR46 (site I) and **b** ItypOR49 (site II). Predicted binding of (*R*)-(−)-ipsdienol (rose) and (*S*)-(+)-ipsdienol (green) to **c** ItypOR46 (site I) and **d** ItypOR49 (site II). Binding site I is located approximately midway in the transmembrane region in relation to the plasma membrane. The shallower predicted binding site II is near the extracellular opening of the binding cavity. Potential hydrogen bonds are indicated with dashed lines. Residues in ORs that adopt a new position upon docking of a ligand are colored according to the corresponding ligand

In ItypOR46, both enantiomers of ipsenol interacted via their conjugated double bonds in a π-π electron interaction with Tyr84, while being able to form a hydrogen bond to Thr205 as well as to Tyr84 at site I (Fig. 6a). Hence, their poses were not sufficiently different to account for the enantiomer discrimination of the OR. The corresponding residues in ItypOR49 are Phe87 and Gly203. Therefore, both of these hydrogen bond interactions are absent in ItypOR49, and ipsenol was consequently instead found at site II, contacting transmembrane helices 3 and 5 lined by Phe313 and hydrogen bonded to Tyr175 and Ser181 ((*R*)-(+)-ipsenol) or hydrogen bonded to Gln153 and lined by Phe313 and in a π-π interaction with Tyr175 ((*S*)-(−)-ipsenol) (Fig. 6b).

The same distribution between sites holds true for the ipsdienol enantiomers. In ItypOR46, the π-π interactions resulting from the docking simulations were similar to ipsenol π-π stacking to Tyr84, but no hydrogen bond to Thr205 at site I was observed. Instead, a hydrogen bond between the hydroxy group and Gln150 was favored (Fig. 6c). Noteworthy, none of the 20 top poses of the ipsdienol enantiomers featured both a π-π stacking to the Tyr84 and a hydrogen bond to Thr205 in ItypOR46, substantially diminishing the favorable interaction as compared to the ipsenol enantiomers. Docking of the ipsdienol enantiomers into the ItypOR49 cleft resulted in binding to site II, involving π-π interactions with Phe313 for both enantiomers, hydrogen bond interactions to Tyr175 from extracellular loop 2 as well as to the backbone amine of Ser181 (Fig. 6d). The favorable hydrogen bonds to the hydroxy group at the stereogenic center resulted in differing side chain rotamers and spatial occupancy of the ligand. As for ItypOR46, elucidating the enantiomer-specific activation of ItypOR49 is likely to require knowledge of the conformation of the open ion pore state.

Poses of the inactive compound 1-hexanol did not cluster into any one specific site, but instead offered hydrogen bond donation to backbone carbonyls and hydrophilic side chains, while the extended hydrocarbon chain engaged in unspecific hydrophobic interactions. Myrcene engaged in unspecific hydrophobic interactions and in π-π electron stacking with either an aromatic moiety at the bottom of the cleft (Tyr84 in ItypOR46 and Phe87 in Ityp49) or with a pair of phenylalanines in transmembrane helix 5 (ItypOR46: Phe316/Phe319; ItypOR49: Phe313/Phe317). Likewise, amitinol with its three double bonds, two of which are conjugated as in the myrcene structure, also interacted with the same aromatic residues in π-π stacking with the aforementioned residues. Having a tertiary alcohol group, amitinol is available for hydrogen bonding interactions, but in the confines of the binding cleft they are not equivalent to those available to the ipsenol and ipsdienol enantiomers.

### Site-directed mutagenesis of predicted ligand-binding residues

**Fig. 7:**
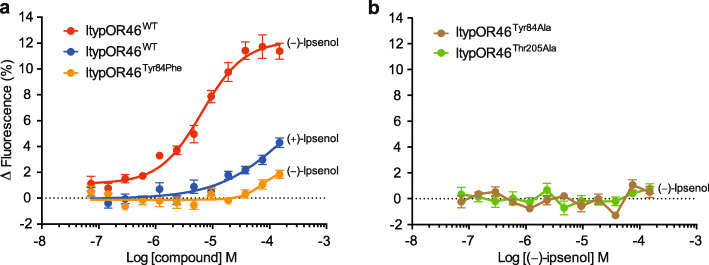
Response to ipsenol enantiomers of cells expressing wildtype (WT) ItypOR46 and mutated versions of this receptor. Data are split between two panels for clarity. **a** ItypOR46^WT^ (positive control; *n* = 3 biological replicates, each including 3 technical replicates, i.e., *n*_total_ = 9) and ItypOR46^Tyr84Phe^ (*n* = 4 biological replicates, i.e., *n*_total_ = 12). **b** ItypOR46^Tyr84Ala^ and ItypOR46^Thr205Ala^ (*n* = 3 biological replicates, *n*_total_ = 9 for both cell lines). For clarity, data for (*R*)-(+)-ipsenol are only shown for ItypOR46^WT^, since this compound did not activate mutated versions of the receptor. Data represent mean responses ± SEM

To gain support for the docking analysis, we introduced mutations to the two predicted key residues (Tyr84 and Thr205) at site I in ItypOR46 and used the HEK cell assay to test the responses of mutated versions of the OR to the enantiomers of ipsenol. Because ipsenol was predicted to form a hydrogen bond with the hydroxy group present on the aromatic moiety of the tyrosine, we introduced two mutations to Tyr84: Tyr84Phe and Tyr84Ala. The former mutation removed the hydroxy group but retained the aromatic structure, whereas the latter mutation removed both features. Responses to (*S*)-(−)-ipsenol in cells expressing ItypOR46^Tyr84Phe^ were highly reduced as compared to responses in the wildtype receptor (included as control) and only observed at the highest concentrations (Fig. 7a). In fact, the responses to (*S*)-(−)-ipsenol in this mutated OR were lower than the responses to (*R*)-(+)-ipsenol in the wildtype receptor. Cells expressing ItypOR46^Tyr84Ala^ did not respond to (*S*)-(−)-ipsenol at any concentration (Fig. 7b), supporting the prediction of hydrogen bonding between the tyrosine and ipsenol at this site, but also suggesting that the presence of an aromatic residue has some importance. To investigate the functional importance of residue 205, we mutated this residue from Thr to Ala. Again, responses to (*S*)-(−)-ipsenol of cells expressing ItypOR46^Thr205Ala^ were completely abolished (Fig. 7b). None of the mutated versions of ItypOR46 responded to (*R*)-(+)-ipsenol. To investigate whether any of the point mutations had resulted in a shift in ligand specificity, the three mutated versions of ItypOR46 were also tested for responses to all screening compounds at 30 μM concentration. Whereas ItypOR46^Tyr84Phe^ and ItypOR46^Thr205Ala^ did not respond to any of these additional compounds, small but significant responses were elicited in ItypOR46^Tyr84Ala^ by (+)-α-pinene (Δ fluorescence = 2.5%; F_1,9_ = 10.94; *p* = 0.009) and (−)-limonene (Δ fluorescence = 2.0%; F_1,9_ = 7.46; *p* = 0.023), suggesting a shift in specificity as these compounds did not activate wildtype ItypOR46 (Supplementary Figure 7, Additional file 2). Proteins of all mutated versions of ItypOR46 were detected by Western blot at equivalent or higher band intensities as the wildtype receptor, indicating sufficient levels of mutated proteins in the cells (Supplementary Figure 1, Additional file 2). Moreover, cell lines expressing mutated versions of ItypOR46 displayed normal VUAA1 responses (Supplementary Figure 7, Additional file 2). The low response of ItypOR49 rendered this receptor unsuitable for mutagenesis experiments.

### Whole mount fluorescence in situ hybridization

**Fig. 8:**
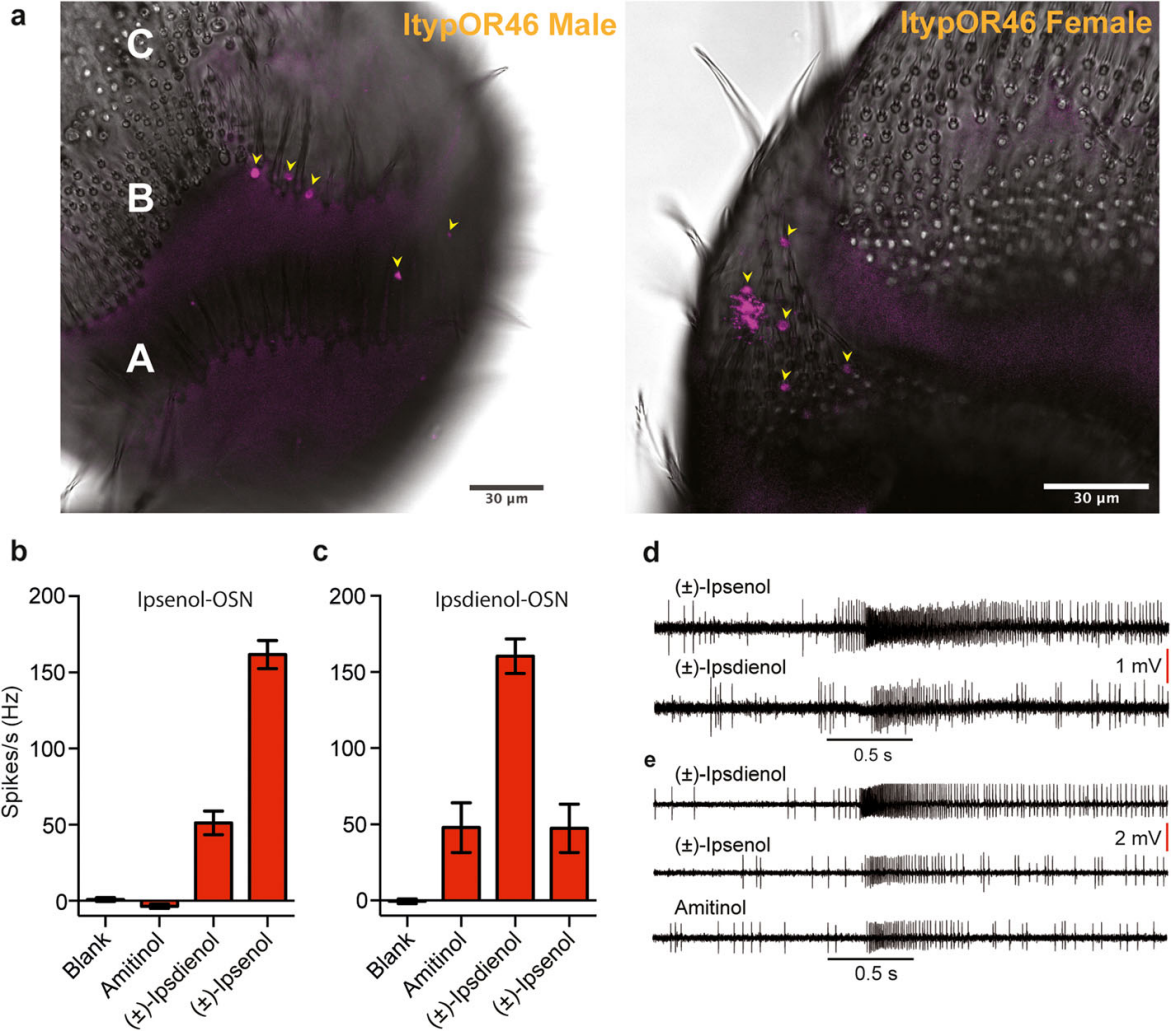
In situ hybridizations and responses of olfactory sensory neurons putatively associated with ItypOR46 and ItypOR49. **a** Detection of ItypOR46 in single-walled sensillum type 1 in antennal areas “A” and “B” in a male (left) and female (right) *I. typographus* as shown by whole mount in situ hybridization using a digoxigenin-labeled probe (magenta; sensilla also highlighted with yellow arrowheads). White capital letters (A, B, and C) **a**, indicate the three areas of sensilla [58], and ipsenol-specific olfactory sensory neurons (OSNs) are found in areas A and B [2, 29]. Electrophysiological responses of the two OSN classes tuned to **b** ipsenol (*n* = 10; 6 from males, 4 from females) and **c** ipsdienol (*n* = 4; 2 from each sex) to OR and/or OSN-active compounds and solvent blank, with representative excitatory response traces shown in **d** and **e**, respectively. Compounds were diluted in paraffin oil and presented at a high dose of 10 μg on filter paper inside standard Pasteur pipette stimulus cartridges. The black horizontal bars indicate the 0.5 s odor stimulation. OSN data in **b–e** originally collected by Andersson et al. [29], but re-analyzed for the purpose of the present study. For data showing the enantio-selectivity of these OSN classes, see original publications [30, 32, 33]

Antennal olfactory sensilla in *I. typographus* are localized in two bands, labeled “A” and “B” (proximal and medial, respectively), that transverse the ventral surface of the antennae, and a distal “C” area [58]. OSN classes tuned to ipsenol and ipsdienol, respectively, are in both sexes found inside single-walled sensillum type 1 (SW1) in the A and B bands of sensilla, but not in area C where the longer SW2 is most abundant [2, 29, 58]. Hence, we performed whole mount in situ hybridizations with digoxigenin-labeled probes against ItypOR46 and ItypOR49 to investigate whether the antennal localization of these receptors corresponds to the distribution of the ipsenol and ipsdienol OSN classes. As expected, signals for ItypOR46 were detected underneath SW1 in the A and B bands of sensilla, but not in area C (Fig. 8a). The sparse and defined signals only in these antennal areas and sensillum type suggest a high specificity of the hybridization. ItypOR49 could not be detected from any of the included antennae using this method.

## Discussion

We sequenced a new antennal transcriptome of *I. typographus* and re-annotated its expressed ORs. The current dataset represents a marked improvement of the originally reported OR repertoire of this species [43], including the identification of 35 novel ORs, 52 full-length ORs, and the essential co-receptor Orco. This, in turn, allowed us to perform functional characterization of the first bark beetle ORs and predict their binding sites, including amino acid residues with which the ligands seem to interact. The identification of ItypOrco also allowed us to record the first responses of a coleopteran Orco to the agonist VUAA1 [59].

### The *I. typographus* ORs and their phylogenetic relationships

The number of ORs (73) in the *I. typographus* transcriptome is close to the number of putatively functional ORs (79) in the genome of the mountain pine beetle *D. ponderosae* [44] and clearly higher compared to previous analyses of antennal transcriptomes of this and other species of bark beetles [43, 60], suggesting a good coverage of our analysis. The larger number of ORs compared to the original ItypOR dataset is likely due to improved sequencing depth and methodology [43]. A recent phylogenetic analysis of ORs from ten species across the Coleoptera identified nine major OR clades [44]. It showed that OR-lineage radiations and losses among the nine clades differ remarkably between taxa, but also that OR distributions are more similar between closely related beetle families. Accordingly, both species of bark beetles included in our analysis have most of their receptors in OR group 7 and 5A, and both lack ORs from groups 3, 4, 5B, and 6. Similar distributions were reported also from other species of the Curculionidae family [60, 61]. In contrast, the OR distributions in other beetle families, including the relatively closely related cerambycids (e.g., *A. glabripennis*) [62], are different, with most species having representatives from several of the groups missing in bark beetles, and a lower proportion of ORs in group 7. Whether these differences are adaptive and relate to ecological specializations or if they represent chance evolutionary events [8] remains unknown. The general lack of simple OR orthology across beetle families, however, suggests that convergent evolution is an important driver for the function of ORs, since unrelated beetle species often have OSNs specialized for the same compounds, such as green leaf volatiles [29, 63]. The phylogenetic positions of the pheromone receptors ItypOR46 and ItypOR49 in relation to the positions of characterized receptors in *M. caryae* suggest that pheromone receptors in beetles do not cluster in specific clades as they do in Lepidoptera [36].

### Specific detection of ipsenol and ipsdienol by ORs and OSNs

The current ItypOR dataset forms an important platform for functional characterization. Among the comparatively large number of characterized OSN classes in this species [2, 18, 29–33] are three well-characterized OSN classes that respond strongly and specifically to (*S*)-(−)-ipsenol, (*R*)-(−)-ipsdienol, and (*S*)-(+)-ipsdienol, respectively [30, 32, 33], whereas no OSNs have been identified for the non-natural (*R*)-(+)-ipsenol [30]. The three OSN classes show no obvious difference in their relative abundance between sexes [29, 30]. Pioneering single sensillum recordings (SSR) elucidated the enantio-specificity of these OSNs and revealed a high specificity when testing a variety of bark beetle pheromone compounds and structural analogs [30, 32, 33]. More recent SSR studies using expanded odor panels with up to 85 stimuli (largely overlapping with the stimuli in the present study) confirmed the high specificities of the ipsenol- and ipsdienol-detecting neurons [2, 29] (Fig. 8b-e). In these two studies, a combined total of 362 sensilla were screened, showing that ipsenol and ipsdienol do not activate OSN classes with other known key ligands to any significant extent (apart from ipsdienol eliciting a clear secondary response in the OSNs responding primarily to amitinol), nor do they specifically activate OSNs with low sensitivity to these compounds only [2, 29]. This suggests that the olfactory system of *I. typographus* has evolved to detect these compounds primarily via three classes of dedicated OSNs, which are similarly specific for their respective key ligands as the two OSN classes tuned to the aggregation pheromone components *cis*-verbenol and 2-methyl-3-buten-2-ol, respectively [29].

(*S*)-(−)-ipsenol and (*R*)-(−)-ipsdienol are produced by male *I. typographus* [24] during the later attack phases, i.e., the production starts when males have admitted females (approx. 2–3 days after initiation of boring), and production peaks when females have started to lay eggs (after approx. 2–6 days) [22]. Field experiments have shown a clear antagonistic effect of ipsenol on the attraction of both sexes to the aggregation pheromone (“shut-off” signal for aggregation), whereas behavioral effects of ipsdienol are less clear and concentration dependent [25]. Here, we report two ORs that specifically detect these compounds and may hence be the ORs that are associated with the abovementioned OSN classes. In HEK cells, ItypOR46 responded exclusively to ipsenol, whereas a secondary response to ipsdienol was recorded in the oocytes. The OSNs tuned to (*S*)-(−)-ipsenol also showed a secondary response to ipsdienol (Fig. 8b, d), and our responses from the oocytes therefore match the in vivo data [29, 30], whereas the HEK cell recordings suggest a higher specificity. Also, the discrimination between ipsenol enantiomers appeared higher in HEK cells compared to oocytes. The reason(s) for the system-dependent specificity of this OR remains obscure, but may be due to differences between HEK cells and oocytes in their cell membrane compositions, affecting the folding of the receptor and access to the ligand binding site. System-dependent OR responses were recently documented for moth ORs [47]. The weak response to amitinol of ItypOR46 in oocytes is not reflected at the OSN level (Fig. 8b) and was likely due to the 3% ipsdienol impurity in the stimulus. This could also explain why “amitinol” was inactive on ItypOR46 in HEK cells (i.e., ItypOR46 did not respond to ipsdienol in this system). Our analysis of OR gene expression indicated that ItypOR46 is the third most highly expressed OR gene in this species, and its high expression correlates with the high abundance of ipsenol-responsive neurons in the antenna [2, 29, 30]. Additionally, results from our in situ experiments showed that ItypOR46 is expressed in the morphological sensillum type SW1 in the A and B bands of antennal sensilla in both sexes, which corresponds to the localization of the OSN class specific for (*S*)-(−)-ipsenol [29]. However, given the high expression of ItypOR46, surprisingly few signals were detected. This, in combination with the lack of detection of the less highly expressed ItypOR49 suggests that our whole mount in situ protocol was not particularly efficient for these tiny and hard beetle antennae.

Our HEK cell data for ItypOR49 showed a specific response to ipsdienol, but this response was weaker than the response of ItypOR46 to ipsenol. In contrast, the VUAA1 responses of Orco in these two cell lines were comparable, which is expected since the two OR cell lines were generated from the same Orco cell line. Our Western blot analysis revealed only a faint band for ItypOR49 (from two independent cell lines), which suggests that protein expression of this OR was low, probably underlying the weak response. Insufficient expression has been highlighted as a limitation for functional characterization of insect ORs using HEK cells with transient expression [64]. This hypothesis is also in line with results from our HEK cell system stably expressing ORs from this and previously tested species, where strongly responding ORs typically have been distinctly detected by Western blot and non-responding or weakly responding ORs as fainter bands, or not detected at all [36, 47, 65, 66], including the five ItypORs related to ItypOR46 and ItypOR49. Alternatively, ItypOR49 may be tuned to a compound not included in the odor panel. However, since all known key ligands of previously characterized OSN classes were tested on the OR and because no OSN class has displayed a selective but weak response to ipsdienol, this scenario appears less likely. ItypOR49 did not respond in oocytes, which also may be due to poor expression or incorrect protein folding. Unfortunately, our attempts to detect any of the two tested ORs or Orco in oocytes by Western blot were unsuccessful; hence, the reason for the silence of ItypOR49 in this system remains unknown. The OSNs tuned to (*R*)-(−)-ipsdienol displayed secondary responses to ipsenol and amitinol [29, 32] (Fig. 8c, e), but these responses were not observed for ItypOR49, which may be due to the overall low responses of this OR in the HEK cells. The antennal expression of the ItypOR49 gene was lower than that of ItypOR46, which correlates with the somewhat lower abundance of ipsdienol-responsive OSNs reported in previous studies, although several additional OR genes showed similar expression levels as ItypOR49 [29, 30].

Similar to previous OSN recordings, especially our HEK cell data reveal high selectivity for enantiomers of ipsenol and ipsdienol in the two ORs, with apparent responses to the non-selected enantiomer being mostly explained by presence of small enantiomeric impurities. Ipsenol and ipsdienol are produced and have been shown to affect behavior and/or trigger OSN responses in several *Ips* and *Dendroctonus* species [33, 67, 68]. Yet, orthologs to ItypOR46 and ItypOR49 have not been identified in the *Dendroctonus* species investigated so far [44, 60], again suggesting that convergent evolution is an important driver for OR function in beetles, even within a taxonomic subfamily. Whether the enantiomers of the two pheromone compounds are detected by orthologous ORs in other *Ips* species remains to be investigated by identifying ORs from additional species in this genus. Also, whether ItypOR46 and ItypOR49 are the sole receptors in *I. typographus* that detect these enantiomers of ipsenol and ipsdienol should be investigated through functional characterization of additional ItypORs. Likewise, future studies should aim to characterize the five remaining ORs in the *Ips*-specific OR radiation that contains ItypOR46 and ItypOR49 to unravel whether these receptors respond to other pheromone compounds, or compounds with different biological origins.

### Prediction of ligand binding in bark beetle ORs

The recent structure of an Orco tetramer [10] can be used in homology modeling of insect ORs, assuming that OR and Orco proteins fold similarly to adopt similar structures. This assumption is reasonable because ORs and Orco are believed to share a common ancestor [6] and because structural features of proteins generally are more conserved than their functions. Similar to the Orco structure [10], our models of ItypOR46 and ItypOR49 revealed a cleft exposed to the extracellular side. Based on its location, it is reasonable to assume that this cleft is important for ligand binding, and this assumption is further supported by numerous studies of other ORs where mutations to residues lining this cleft have affected the responses (summarized in [54]). Our docking analysis towards this cleft suggested two discrete binding sites in ItypOR46 and ItypOR49, respectively. Apart from (*R*)-(+)-ipsenol and (*S*)-(+)-ipsdienol, the inactive compounds were not predicted to interact sufficiently with these residues. Ipsenol was predicted to interact with Tyr84 and Thr205 at site I in ItypOR46, whereas ipsdienol did not interact with the latter residue, which may relate to its lower activity on this OR. The corresponding residue to Tyr84 (Val91) in DmelOR59b has been shown to be central for the inhibitory effect of DEET on odor detection [55]. This residue is also adjacent to one of the sites that affects VUAA1 responses in Orco proteins from different species [53]. Interactions between enantiomers of the two active compounds to Gln150 in ItypOR46 and the corresponding Gln153 in ItypOR49 were also observed, and this residue has been shown to be important for the responses to 2-heptanone in DmelOR85b [69]. Based on its robust responses to ipsenol, we targeted ItypOR46 to provide experimental support for the predicted importance of Tyr84 and Thr205 in ligand binding. Mutating any of these residues to alanine completely abolished the response to ipsenol, whereas cells expressing ItypOR46^Tyr84Phe^ retained a small response at the highest stimulus concentrations. The latter response indicates that this mutant OR is likely correctly folded, although severely affected in the binding of ipsenol. Because the aromatic phenylalanine differs from tyrosine only by lacking the hydroxy group, the predicted hydrogen bonding between ipsenol and Tyr84 was supported, suggesting that this interaction is crucial for the mechanism leading to opening of the ion channel of the receptor complex. Similarly, the response shift towards (+)-α-pinene and (−)-limonene shown in the ItypOR46^Tyr84Ala^ mutant is in line with the hypothesis that ligand binding was affected, rather than protein stability or folding. In contrast, due to the lack of any response in ItypOR46^Thr205Ala^, it is possible that this mutation affected the stability or folding of the protein, even though our docking analysis predicts a role in ligand binding and alanine is present at this position in 18 ItypORs, including ItypOR49 (Supplementary Table 1, Additional file 1; Supplementary Data 1, Additional file 3).

Opening of the ion channel upon binding of ligands is likely to involve a two-step mechanism—binding of the activating ligand, followed by a conformational change transmitted to helix 7, which is blocking the ion channel in the tetrameric Orco complex. The structure of the *Apocrypta bakeri* Orco [10] revealed the closed ion channel structure, and as such leaving entry into ligand binding sites open. In analogy with mechanisms postulated for other ion channels, opening of the channel results from ligand binding, and it follows that the open state binding site must have higher affinity than the binding site at the closed state in order to drive the conformational change [70]. Additionally, because the cleft is water filled and primarily hydrophobic, as revealed by the Orco structure and likewise our OR homology models, binding of aliphatic chain ligands could likely favor the open state by lowering the free energy barrier required for the transition as well, via expulsion of water molecules. Without a structure of the open channel, the underlying mechanism of channel opening upon binding of ipsenol and ipsdienol, and how the different enantiomers are discriminated by the ORs remain obscure. Our findings also raise questions regarding how ligand specificity may evolve in insect ORs. Ipsdienol differs from ipsenol only by the presence of an additional double bond. Yet, the two compounds are detected by different ORs that only share 43.4% amino acid identity. Although all of this variation is unlikely to affect selectivity, this suggests that complex molecular changes may underlie specificity shifts in insect ORs detecting similar compounds. On the other hand, such major changes may be required for ORs to display such a high discrimination for compounds being that similar. Further investigation is needed to understand the molecular evolution that determines ligand selectivity in insect ORs; in particular, revealing the structure of a ligand-binding OR would be especially rewarding.

## Conclusions

We report a high-quality complement of ORs in *I. typographus*, which allowed us to functionally characterize the two first bark beetle ORs, specifically responding to (*S*)-(−)*-*ipsenol and (*R*)-(−)-ipsdienol. Responses from the ORs correspond well with those of previously characterized OSN classes, and the OR expression levels and antennal distribution are consistent with the antennal frequency and distribution of these OSNs, especially for ItypOR46. Our investigation of the ligand-OR interaction predicted two discrete binding sites and suggested that hydrogen bonding is important for the binding of ipsenol in ItypOR46. It remains to be investigated whether these putative binding sites are conserved across the insect OR family or if the predicted binding cleft may present a continuum of binding sites in different ORs. Also, further work is needed to investigate whether broadly tuned ORs contain single discrete binding sites, or if they interact with their multiple ligands at different sites.

Because ipsenol elicits strong antagonistic effects on pheromone attraction in *I. typographus*, ItypOR46 could be a target to employ in screenings aimed to identify more potent agonists than the natural ligand. Such a screening can now be directed towards the predicted binding site, and such agonists may be used in the development of more efficient repellents for forest protection. Indeed, agonists that elicit ultra-prolonged activation of the carbon dioxide-sensitive neurons of mosquitos have been identified, with extended effects on host-seeking behavior [71]. Whether or not similar compounds can be identified for ItypOR46, and how effectively they will divert attacks, remains to be investigated. Additionally, the widespread production of ipsenol and ipsdienol across many species of bark beetles makes both ItypOR46 and ItypOR49 suitable candidates to be used in sensitive biosensors [15] for detection of infestations of different bark beetles. Although there are technical challenges to overcome before such sensors can be used for airborne volatiles in a field situation, detection of infestations and removal of attacked trees before the next generation emerges to infest new trees are crucial to limit bark beetle populations, and hence outbreaks and economic loss.

## Methods

### Insect material and RNA isolation

*Ips typographus* individuals originated from a laboratory culture reared on Norway spruce (*P. abies*) logs and were kindly provided by Prof. F. Schlyter. The antennae from 255 adults (males and females combined in an equal sex ratio) were homogenized using Tissue-tearor model 98370-365 (Bartlesville, OK, USA), and total RNA was isolated using the RNeasy Minikit (Qiagen, Hilden, Germany). This yielded 6.2 μg of high-quality total RNA that was used for transcriptome sequencing and molecular cloning.

### Transcriptome sequencing, annotation, expression levels, and phylogenetic analysis of ORs

DNase-treated RNA was subjected to poly-A enrichment and library construction using a RNA-Seq v2 Library Preparation Kit (Illumina, San Diego, CA, USA), followed by 150 bp paired-end sequencing, performed on an Illumina HiSeq 3000 platform at the Max Planck-Genome center (Cologne, Germany). The sequencing yielded 31,622,325 paired-end reads post quality appraisal and initial read filtering using standard methods. Low-quality reads and adaptor sequences were removed. The high-quality reads were de novo assembled using the short reads assembly program Trinity version 2.3.2 [72] as well as a CLC Genomics Workbench version 10 (Qiagen, Carlsbad, CA, USA). The Trinity assembly yielded a total of 74,151 predicted “genes” with their isoforms totaling 171,567 predicted “transcripts” (i.e., on average 2.3 assembly isoforms per predicted gene) with average length of 1487 bp and N_50_ = 3373 bp. The CLC assembly resulted in 47,576 assembled contigs with an average length of 1033 bp and N_50_ = 1488 bp. The overall completeness of these assemblies was assessed using the Benchmarking Universal Single-Copy Orthologs (BUSCOv3.0.1; https://busco.ezlab.org/) tool performed against the Insecta odb9 dataset, which included 1658 reference genes [73]. This analysis indicated that the percentage of complete BUSCOs was 97.4 for the Trinity assembly and 80.1 for the CLC assembly, but also a higher percentage of duplicated BUSCOs in the Trinity assembly (Supplementary Table 3, Additional file 2). The sequence reads have been deposited in the SRA database at NCBI under the BioProject accession number PRJNA602798.

Sequences of *I. typographus* ORs (ItypORs) were annotated through tBLASTn searches against the abovementioned assemblies using query sequences from *I. typographus*, *D. ponderosae* (Curculionidae), *A. glabripennis* (Cerambycidae), and *Leptinotarsa decemlineata* (Chrysomelidae) [43, 74, 75]. An *e*-value cut-off at 1.0 was used to account for the divergent nature of this gene family. All identified ItypORs were included in additional BLAST searches until all novel hits were exhausted. Due to the difference in assembly completeness, the majority of the ItypOR sequences were identified from the Trinity assembly, with only a few ORs being more complete in the CLC assembly (Supplementary Table 1, Additional file 1). A few OR sequences could be extended or completed by joining overlapping sequences from the two current assemblies and/or the published assembly [43]. Short transcripts encoding partial OR sequences that did not overlap with other ItypOR sequences in multiple sequence alignments were discarded to ensure that all reported ORs were unique. The previously identified ORs (ItypOR1–43) retained their original names, and novel ORs were given names from ItypOR44 to ItypOR77 in the order they were identified. Some of the partial original OR sequences [43] were here discarded as assembly isoforms, but their numbers were not recycled for any of the novel ORs to avoid confusion. Transcripts did not always encode full-length OR sequences. Hence, suffixes were added to gene names following established practice [45], with NTE and CTE suffixes given to genes with the N-terminus or C-terminus missing, respectively. A FIX suffix was given to genes that were annotated on transcripts which were manually corrected using raw RNA-seq reads or following the other current or previously published assemblies [43]. In cases where genes had multiple suffixes, one-letter abbreviations were used in combinations (i.e., N, C, and F).

To analyze OR gene expression, clean reads were mapped to the open reading frames (ORF) of annotated ItypOR genes using the align_and_estimate_abundance.pl script from the Trinity v2.8.2 software package [76] using default parameters except for --est_method RSEM --aln_method bowtie2 --trinity_mode. The rationale for mapping to the ORFs of OR genes, and not to all transcripts in the assembly, was because some OR genes were only present in one of the two assemblies (CLC or Trinity) used for the annotation (such genes would thus not be covered by the analysis) and some OR transcripts contained misassembled fragments in non-coding regions, which could bias the estimated expression level of the OR gene.

The amino acid sequences of the ItypORs were aligned with the ORs from the genomes of *D. ponderosae* (Dpon) [45] and *A. glabripennis* (Agla) [74] using MAFFT 7.017 [77], implemented in Geneious software package 7.1.9. The three functionally characterized ORs from *M. caryae* (Mcar) (Cerambycidae) [39] were also included to indicate their positions in the phylogeny. Pseudogenes and partial ORs below 200 amino acids from *A. glabripennis* were excluded to improve the alignment and reduce the size of the tree. Uninformative regions of the alignment were excised using trimAl v1.2 [78] with the following settings: similarity threshold 0, gap threshold 0.7, and minimum 25% conserved positions. Partition finder 2 [79] was used to select a model of evolution, with the best fit obtained for a JTT amino acid substitution matrix, a proportion of invariant sites, gamma distributed rate variation, and empirical equilibrium amino acid frequencies (JTT+I+G+F). These parameters were used to construct a maximum likelihood tree using RAxML 8.1.2 [80, 81], with branch support calculated by rapid bootstrapping (*N* = 100). The tree was visualized, rooted with the Orco lineage, and color coded in FigTree 1.4.3. Final graphical editing was performed using Adobe Illustrator.

### First-strand cDNA synthesis and confirmation of OR sequences

First-strand cDNA was synthesized from 1 μg of DNase-treated antennal RNA using the ThermoScript RT-PCR system for First-Strand cDNA Synthesis (Thermo Fisher Scientific, Carlsbad, CA, USA), according to the manufacturer’s instructions, except for using both random hexamers and oligo-dT primers in the reaction. Multiple sequence alignments of the initial set of ItypORs annotated here indicated that eight OR fragments likely belonged to non-overlapping parts of the same four genes (ItypOR57NTE, ItypOR61NTE, ItypOR70FN, and ItypOR71NTE). Hence, PCR amplification from cDNA followed by Sanger sequencing of the PCR products were performed to verify these joins and to add internal DNA sequence that were missing on the transcripts (25–45 bp). These partial genes were amplified using Pfu Phusion Flash high-fidelity master mix (Thermo Fisher Scientific), and with the forward primer designed for the most N-terminal transcript and reverse primer for the most C-terminal transcript. The PCR products were resolved on 1% TAE agarose gels, and bands of expected length were excised and purified using the Wizard® SV Gel and PCR clean-up system (Promega). Sequencing PCR was performed using the purified PCR products, their gene-specific primers, and the BigDye® Terminator v1.1 Cycle Sequencing Kit (Thermo Fisher Scientific). Sanger sequencing was performed using an Applied Biosystems™ capillary 3500 Genetic Analyzer (Thermo Fisher Scientific) at the sequencing facility at the Department of Biology, Lund University.

### Molecular cloning of ItypOrco and ItypORs for functional characterization in HEK293 cells

Sequences of ItypOrco and ItypORs were amplified from antennal cDNA, using full-length gene-specific primers and the Pfu Phusion Flash high-fidelity master mix. The PCR products were purified as described above, and then included in a second PCR reaction, but this time using extended primers to add a 5′ NotI recognition site, a Kozak sequence (“cacc”) and an N-terminal epitope tag (c-Myc for ItypOrco and V5 for ItypORs), as well as a 3′ ApaI recognition site. The PCR products were purified and then digested using NotI and ApaI restriction enzymes (NEB, Ipswich, MA, USA). The modified OR sequences were separated by gel electrophoresis, and bands of expected length excised, purified and ligated into the expression vectors pcDNA™4/TO (Orco) and pcDNA™5/TO (ORs) (all Thermo Fisher Scientific), followed by transformation into HB101 competent cells (Promega). Successful transformation was confirmed by colony PCR, and positive colonies were grown in LB broth overnight with ampicillin. Plasmids were extracted using the GeneJET Plasmid Miniprep kit (Thermo Fisher Scientific), and then Sanger sequenced. Plasmids with verified Orco or OR sequence were transformed into competent cells, and positive colonies were identified by colony PCR and then grown in LB broth overnight. Large quantities of purified plasmids were obtained using the PureLinkTM HiPure Plasmid Filter Midiprep kit (Thermo Fisher Scientific). The cloned sequences of ItypOrco, ItypOR46, and ItypOR49 have been deposited in GenBank under the accession numbers MN987209-MN987211.

ItypOR46 was subjected to site-directed mutagenesis to verify the functional importance of two residues (Tyr84 and Thr205) predicted (see below) to be central for ligand binding. Three point mutations (Tyr84Phe, Tyr84Ala, and Thr205Ala) were introduced individually to ItypOR46 in pcDNA5™/TO using the Q5 Site-Directed Mutagenesis kit (New England Biolabs) following the manufacturer’s instructions. Successful mutants were identified using Sanger sequencing, and large quantities of plasmids obtained as described above.

### Generation of inducible cell lines expressing ItypOrco and ItypORs, and confirmation of protein expression

HEK293 cells (originating from ATCC) stably expressing ItypOrco and ItypORs were produced and cultured according to previously described methods [46]. Briefly, an isogenic, tetracycline repressor-expressing (TREx) cell line [46] was transfected with pcDNA™4/TO/ItypOrco, and cultured using zeocin and blasticidin selection antibiotics (NEB). Afterwards, this TREx/ItypOrco-expressing cell line was tested in a fluorescent calcium assay (described below) against the Orco agonist VUAA1 (> 98% purity, Sigma-Aldrich), which directly activates the Orco protein in nearly all insect species tested to date [53, 59, 65], for confirmation of functional Orco expression. The TREx/ItypOrco cell line was then used in separate transfections with pcDNA™5/TO/ItypOR46, pcDNA™5/TO/ItypOR49, and mutated versions of ItypOR46 and cultured as described above, but with the addition of the pcDNA5™/TO-specific selection antibiotic hygromycin (Gold Biotech). For functional testing, stable TREx/ItypOrco/ItypOR cell lines (tested negative for Mycoplasma contamination) were cultured for a maximum of 6 weeks or 15 passages (without any obvious change in growth rate, cell morphology, or function) and then discarded. New aliquots of cells from our cell line “bank” were then used for continued culturing and testing if necessary [46]. The TREx/ItypOrco/ItypOR cell lines were analyzed for protein expression of myc-tagged Orco and V5-tagged ORs by Western blot. Cells were cultured, induced to express exogenous Orco and ORs and pelleted as previously described [46], with non-induced cells included as controls. The protein extraction and blotting also proceeded according to previously described methods [36].

### Functional characterization in HEK293 cells

Cells transfected with ItypOrco alone, and ItypOrco in combination with each of the ItypORs, were tested for responses to compounds in the previously described fluorescent calcium assay [36, 46, 65]. Briefly, cells were plated into poly-D-lysine-coated 96-well plates and induced to express ItypOrco and ItypORs. Half of the wells were left non-induced to serve as a negative control. One hour prior to the assay, the wells were loaded with a calcium-sensitive fluorophore (Fluo4-AM, Thermo Fisher Scientific) and incubated at room temperature for 30 min, after which cells were washed and assay buffer was added to wells. After 30 additional min, cells were investigated for ligand-induced receptor activation using a FLUOstar Omega plate reader (BMG Labtech, Ortenberg, Germany). Cells were tested in triplicates (technical replicates) on each plate (biological replicate). The cells in each well were subjected to a single stimulation, and then discarded. The recordings proceeded as follows: The background fluorescence was measured from three wells with non-induced cells and three wells with induced cells just before adding a single stimulus into these six wells. The ligand-induced change in fluorescence was then immediately measured from the six wells for every 5 s up to 1 min, starting 10 s post stimulation. For consistency between cell lines and stimuli, and because the ligand-induced responses generally decline over time, the first reading was used to calculate the ligand-induced response. Hence, the fluorescence measured 10 s post stimulation was used to calculate Δ fluorescence (%) for cells in each stimulated well in relation to the background fluorescence of that particular well. The assay was finished when all desired wells of a particular experiment had been stimulated and measured, with a full plate taking approx. 20 min to finish.

Test compounds were diluted in DMSO and assay buffer as previously described [46], with the final DMSO concentration in the wells being 0.5%. The Orco agonist VUAA1 was included in the assays as a control (30 μM concentration) for functional Orco expression, and also in dose-response experiments with the ItypOrco cell line (*n* = 3 biological replicates). Assay buffer with 0.5% DMSO in was included as a negative control (vehicle) in all assays. The two wildtype ItypOrco/ItypOR expressing cell lines were screened against a panel of 68 ecologically relevant compounds (30 μM concentration; *n* = 3 biological replicates), including the pheromones from a variety of bark beetle species, host and non-host compounds, as well as compounds from the fungal symbionts of *I. typographus* (Supplementary Table 2, Additional file 2). This odor panel comprised all known key ligands for the previously characterized OSN classes of this species and several of their secondary ligands [2, 18, 29, 30, 67], along with a few bark beetle pheromone compounds with unknown activity in *I. typographus*. Compound purities were analyzed by gas chromatography-mass spectrometry (GC-MS) (Supplementary Table 2,Additional file 2). Mean ligand-induced responses (± SEM) in ORs were calculated and graphed in GraphPad Prism 6 (GraphPad Software Inc., La Jolla, CA, USA). Ligands that elicited significantly stronger responses in induced versus non-induced cells were regarded as active. Hence, a General Linear Model analysis was performed using IBM SPSS statistics v.23 to identify active compounds, with “induction (yes/no)” included as a fixed factor, and “plate number” as a random factor to account for the variation between plates. Inter-plate variation was never significant (all *p* ≥ 0.092) for any of the active ligands (VUAA1, ipsenol, and ipsdienol on wild type ORs, and (+)-α-pinene and (−)-limonene on mutated ItypOR46) or tested cell lines. Levene’s test of Equality of Error Variances was performed for each statistical model to verify homoscedasticity (all *p* ≥ 0.104). Screening responses below 1% increased fluorescence were regarded as “no response” because they were within the range of random variation of the assay. Active compounds eliciting responses above 3% increased fluorescence at the 30 μM screening concentration were included in subsequent dose-response experiments, which were also designed to elucidate the enantiomer specificities of ItypOR46 (*n* = 4 biological replicates) and ItypOR49 (*n* = 6 biological replicates). The synthesis of ipsenol and ipsdienol enantiomers is described in the Supplementary Methods, Additional file 2. The three mutated versions of ItypOR46, along with the wildtype (WT) ItypOR46 (included as control), were tested against the enantiomers of ipsenol (*n* = 3 biological replicates for ItypOR46^WT^, ItypOR46^Tyr84Ala^, and ItypOR46^Thr205Ala^; *n* = 4 for ItypOR46^Tyr84Phe^), and also screened for responses to the full test odor panel at 30 μM concentration (*n* = 2 biological replicates). Half-maximal effective concentrations (EC_50_) with 95% confidence intervals were estimated using the non-linear curve fit regression function in GraphPad Prism.

### Functional characterization in *Xenopus* oocytes

ItypOR46 and ItypOR49 were also assayed using *Xenopus laevis* oocytes. Gene-specific primers for the two ORs and Orco, designed to include a flanking 5′ Kozak sequence (“gccacc”) and 5′ and 3′ recognition sites (BamHI and XbaI for ORs; EcoRI and XbaI for Orco), were employed in PCR reactions using the OR-containing HEK cell expression vectors as templates. The PCR products were purified, digested and cloned into the pCS2+ expression vector as described above. Large quantities of plasmids containing verified inserts were obtained using the plasmid Maxi kit from Qiagen. The plasmids were linearized using NotI (Promega), and the linearized DNA was purified and transcribed into complementary RNA (cRNA) using the SP6 mMESSAGE mMACHINE® kit (Invitrogen, Carlsbad, CA, USA).

Oocytes were surgically removed from *X. laevis* frogs (purchased from University of Portsmouth, UK) and treated with 1.5 mg/ml collagenase (Sigma-Aldrich, St. Louis, MO, USA) in oocyte Ringer 2 solution (containing 82.5 mM NaCl, 2 mM KCl, 1 mM MgCl_2_, 5 mM HEPES, pH 7.5) at 20 °C for 15–18 min. Stage V–VII oocytes were co-injected with cRNAs from the ItypOrco and ORs (50 ng of each), and then incubated in Ringer’s buffer (96 mM NaCl, 2 mM KCl, 5 mM MgCl_2_, 0.8 mM CaCl_2_, 5 mM HEPES, pH 7.6) containing 550 mg/L sodium pyruvate and 100 mg/L gentamicin at 18 °C for at least 3 days. The previously described two-electrode voltage clamp electrophysiological set-up was used to record whole-cell inward currents from oocytes in good condition (3–6 days post-injection) at a holding potential of − 80 mV [82]. Test compounds were applied to the oocyte chamber by means of a computer-controlled perfusion system at a rate of 2 ml/min for 20 s with extensive washing with Ringer’s buffer at 4 ml/min between stimulations. Data were collected and analyzed using Cellworks software (npi electronic GmbH, Tamm, Germany).

Due to the limited number of channels in the perfusion system, only six compounds were included: VUAA1, the HEK cell-active ligands ipsenol and ipsdienol, and the structurally related compounds amitinol and myrcene, as well as the aggregation pheromone component (−)-*cis*-verbenol. The compounds were dissolved in DMSO and Ringer’s buffer to desired test concentrations and a final DMSO concentration of 0.1%. Ringer’s buffer with 0.1% DMSO served as a negative control. Compounds were screened for receptor activity at a concentration of 30 μM (*n* = 5), and the active compounds were subsequently included in dose-response trials using additional oocytes (*n* = 4–6).

### Whole-mount fluorescence in situ hybridization

Fluorescence in situ hybridization was carried out following previously described protocols [83–85]. Briefly, digoxigenin-labeled antisense riboprobes for ItypOR46 and ItypOR49 were transcribed from linearized recombinant pCS2+ plasmids using the T7 RNA transcription system (Roche). The labeled probes were hydrolyzed to about 800 bp in length with 2× sodium carbonate buffer (80 mM NaHCO_3_, 120 mM Na_2_CO_3_, pH 10.2). Freshly dissected whole-mount antennae were fixed (with 4% paraformaldehyde in 0.1 M NaHCO_3_, pH 9.5, 0.03% Triton X-100) for 24 h at 6 °C on an overhead shaker and permeabilized with 0.2 M HCl. The antennae were then transferred to a drop of PBS buffer on a glass slide and carefully squeezed about 10 times with fine tweezers under binocular control. This created small cracks in the cuticle and facilitated penetration of the reagents into the tissue [79]. The antennae were pre-hybridized for 24 h at 55 °C in the hybridization solution (50% formamide, 5× SSC, 1× Denhardt’s reagent, 50 μg/ml yeast RNA, 1% Tween 20, 0.1% CHAPS, 5 mM EDTA pH 8.0). Single probes were then added to the hybridization solution in 1:100 dilution and hybridized with the antennae for 48 h at 55 °C. Following the blocking step, the probes were detected by anti-digoxigenin AP-conjugated antibodies (Roche; Cat. No. 11 093 274 910) in 1:500 dilution in combination with the substrate Vector Red Alkaline Phosphatase (AP) Substrate Kit (Vector Laboratories, Burlingame, CA, US). After the washing steps, the antennae were mounted in mowiol mounting media. The slides were visualized on a confocal laser scanning microscope (Leica TCS SP8, Leica Microsystems, Wetzlar, Germany) at the Microscopy Facility, Department of Biology, Lund University.

### Protein modeling and ligand docking simulations

Sequence alignment of ItypOR46 and ItypOR49 was performed against a multiple sequence alignment containing 3185 OR and Orco sequences [53, 54] (Supplementary Data 1, Additional file 3), including the sequence for the *A. bakeri* Orco for which a homotetrameric cryo-EM structure was recently published [10]. Homology models of the two ORs were produced in Swiss-Model [86] with the *A. bakeri* Orco structure (PDB ID 6c70) as template. Extracellular loop 2 which is absent in the Orco structure was built using the program SuperLooper2 [87]. The resulting model structures were energy minimized using NAMD [88].

Three-dimensional structures of the two enantiomers of ipsenol and ipsdienol, as well as myrcene, amitinol, and 1-hexanol, were produced, and AutoDockTools 1.5.6 (ADT) was used to convert the ligand structure files to AutoDock ligand format (pdbqt). The ItypOR46 and ItypOR49 homology models were likewise converted to pdbqt format. As in the Orco structure [10], an approximately 20-Å-deep wedge-shaped cavity at the extracellular side, formed by helices 2 to 6, was identified. Residues lining the cavity were defined as flexible and input pdbqt files for AutoDock Vina 1.1.2 [89] were produced using ADT as well as a grid box for molecular docking simulation, covering the entirety of the putative binding cavity. AutoDock Vina 1.1.2 was used to perform molecular docking simulation, and the top 20 poses of each ligand were outputted.

## Supplementary Information

**Additional file 1: Supplementary Table 1.** Annotation details of the *Ips typographus* odorant receptors (ORs), including names, amino acid sequences, expression levels, annotation notes, and correspondence with the original dataset [43].

**Additional file 2: Supplementary Table 2.** Compounds used for characterization of *Ips typographus* odorant receptors (ORs) and Orco, including their purities, source information, and examples of main biological origins. **Supplementary Table 3.** Assessment of the completeness of antennal transcriptome Trinity and CLC assemblies using the Benchmarking Universal Single-Copy Orthologs (BUSCOv3) tool performed against the Insecta odb9 dataset (https://busco.ezlab.org/).**Supplementary Figure 1.** Protein detection of *Ips typographus* odorant receptors (ORs; V5-tagged) and Orco (myc-tagged) from TREx/HEK293 cells by Western blot. *Upper left panel:* Detection of wildtype ItypOR46 and ItypOR49 (two cell lines). *Upper right panel:* detection of Orco in the same cell lines. *Lower panel:* detection of three versions of mutated ItypOR46 proteins and wildtype (WT) ItypOR46 (included as control). Proteins were only detected from cells induced (+) to express ItypORs and Orco, and not from non-induced (−) control cells, indicating proper regulation by the repression system. **Supplementary Figure 2.** Response of TREx/HEK293 cells expressing ItypOR46 and ItypOrco to all stimuli (30 μM) and vehicle control in the screening experiment (*n* = 3 biological replicates, each including 3 technical replicates, i.e., n_total_ = 9). (+)-Induction: response of cells induced to express ItypOrco and ItypOR46; (−)-Induction: response of non-induced control cells. Data represent mean responses ± SEM. **Supplementary Figure 3.** Response of TREx/HEK293 cells expressing ItypOR49 and ItypOrco to all stimuli (30 μM) and vehicle control in the screening experiment (n = 3 biological replicates, each including 3 technical replicates, i.e., n_total_ = 9). (+)-Induction: response of cells induced to express ItypOrco and ItypOR49; (−)-Induction: response of non-induced control cells. Data represent mean responses ± SEM. **Supplementary Figure 4.** A) Current traces of two oocytes expressing ItypOrco/ItypOR49, indicating responses to the Orco agonist VUAA1 and minute responses to racemic ipsdienol. B) Current trace of oocyte expressing myc-tagged ItypOrco and V5-tagged ItypOR46 showing the same specificity as non-tagged versions of these receptors in this system. **Supplementary Figure 5.** Energy minimized model of ItypOR49 (blue cartoon) showing the extended extracellular loop 2 (EL2) overlaid with the published Orco Cryo-EM map (mesh). Homology models of ItypOR46 and ItypOR49 were built using the Orco structure (PDB ID 6c70) as template. The part of EL2 that was not included in the published structure (residues 156–170) was modeled ab initio. An extended β-sheet fold in this part of the loop is supported by the density in the Cryo-EM map. In neither of the models the putative binding cleft is blocked by this part of EL2. The RMSDs between the Cα of the template and energy minimized models for ItypOR46 and ItypOR49 (excluding the ab initio modeled parts) are 0.958 Å and 0.880 Å, respectively. **Supplementary Figure 6.** Chemical structures and IUPAC names of compounds included in the molecular docking analyses against ItypOR46 and ItypOR49. **Supplementary Figure 7.** Response of TREx/HEK293 cells expressing A) ItypOR46^Tyr84Phe^, B) ItypOR46^Tyr84Ala^, and C) ItypOR46^Thr205Ala^ and ItypOrco to all stimuli (30 μM) and vehicle control in the screening experiment (*n* = 2 biological replicates, each including 3 technical replicates, i.e., n_total_ = 6). (+)-Induction: response of cells induced to express ItypOrco and the mutated ItypOR46; (−)-Induction: response of non-induced control cells. Data represent mean responses ± SEM. Asterisks (* *p* < 0.05; ** *p* < 0.01) in B) indicate significant differences between induced and non-induced cells. **Supplementary Methods.** Description of the chemical synthesis of ipsenol and ipsdienol as well as their pure enantiomers.

**Additional file 3:** **Supplementary Data 1.** Multiple sequence alignment used to generate the ItypOR models.

**Additional file 4:** **Supplementary Data 2.** Datasets from the HEK cell and oocyte assays conducted in this study as well as raw data from previous single sensillum recordings.

## Data Availability

The datasets supporting the conclusions of this study are included in this article and its Additional files, with data from HEK cell and oocyte assays, and single sensillum recordings available in Supplementary Data 2, Additional file 4. The sequence reads have been deposited in the SRA database at NCBI (BioProject accession number PRJNA602798) [90]. Cloned sequences of ItypOrco, ItypOR46, and ItypOR49 have been deposited in GenBank (accession numbers MN987209-MN987211) [91].

## Abbreviations

Agla: *Anoplophora glabripennis*
GC-MS: Gas chromatography-mass spectrometry
Dmel: *Drosophila melanogaster*
Dpon: *Dendroctonus ponderosae*
EL: Extracellular loop
HEK: Human embryonic kidney
Ityp: *Ips typographus*
Mcar: *Megacyllene caryae*
OR: Odorant receptor
Orco: Odorant receptor co-receptor
ORF: Open reading frame
OSN: Olfactory sensory neuron
SSR: Single sensillum recordings
SW1: Single-walled sensillum type 1
SW2: Single-walled sensillum type 2
TM: Transmembrane
TPM: Transcripts per million
WT: Wildtype

## References

[1] Hansson BS, Stensmyr MC (2011). Evolution of insect olfaction. Neuron..

[2] Kandasamy D, Gershenzon J, Andersson MN, Hammerbacher A. Volatile organic compounds influence the interaction of the Eurasian spruce bark beetle (*Ips typographus*) with its fungal symbionts. ISME J. 2019;13:1788–800. 10.1038/s41396-019-0390-3 PMC6775991 30872804

[3] Clyne PJ, Warr CG, Freeman MR, Lessing D, Kim J, Carlson JR. A novel family of divergent seven-transmembrane proteins: candidate odorant receptors in *Drosophila*. Neuron. 1999;22:327–38. 10.1016/s0896-6273(00)81093-4 10069338

[4] Kaupp UB (2010). Olfactory signalling in vertebrates and insects: differences and commonalities. Nat Rev Neurosci.

[5] Smart R, Kiely A, Beale M, Vargas E, Carraher C, Kralicek AV, et al. *Drosophila* odorant receptors are novel seven transmembrane domain proteins that can signal independently of heterotrimeric G proteins. Insect Biochem Mol Biol. 2008;38:770–80. 10.1016/j.ibmb.2008.05.002 18625400

[6] Robertson HM, Warr CG, Carlson JR. Molecular evolution of the insect chemoreceptor gene superfamily in *Drosophila melanogaster*. Proc Natl Acad Sci U S A. 2003;100:14537–42. 10.1073/pnas.2335847100 PMC304115 14608037

[7] Andersson MN, Löfstedt C, Newcomb RD (2015). Insect olfaction and the evolution of receptor tuning. Front Ecol Evol.

[8] Nei M, Niimura Y, Nozawa M (2008). The evolution of animal chemosensory receptor gene repertoires: roles of chance and necessity. Nat Rev Genet.

[9] Brand P, Robertson HM, Lin W, Pothula R, Klingeman WE, Jurat-Fuentes JL (2018). The origin of the odorant receptor gene family in insects. eLife..

[10] Butterwick JA, del Mármol J, Kim KH, Kahlson MA, Rogow JA, Walz T (2018). Cryo-EM structure of the insect olfactory receptor Orco. Nature..

[11] Sato K, Pellegrino M, Nakagawa T, Vosshall LB, Touhara K (2008). Insect olfactory receptors are heteromeric ligand-gated ion channels. Nature..

[12] Wicher D, Schäfer R, Bauernfeind R, Stensmyr MC, Heller R, Heinemann SH, et al. *Drosophila* odorant receptors are both ligand-gated and cyclic-nucleotide-activated cation channels. Nature. 2008;452:1007–11. 10.1038/nature06861 18408711

[13] Andersson MN, Newcomb RD (2017). Pest control compounds targeting insect chemoreceptors: another silent spring?. Front Ecol Evol.

[14] Murugathas T, Zheng HY, Colbert D, Kralicek AV, Carraher C, Plank NOV (2019). Biosensing with insect odorant receptor nanodiscs and carbon nanotube field-effect transistors. ACS Appl Mater Interfaces.

[15] Khadka R, Aydemir N, Carraher C, Hamiaux C, Colbert D, Cheema J (2019). An ultrasensitive electrochemical impedance-based biosensor using insect odorant receptors to detect odorants. Biosens Bioelectron.

[16] Khadka R, Carraher C, Hamiaux C, Travas-Sejdic J, Kralicek A. Synergistic improvement in the performance of insect odorant receptor based biosensors in the presence of Orco. Biosens Bioelectron 2020;153:112040. 10.1016/j.bios.2020.112040 31989943

[17] Kurz WA, Dymond CC, Stinson G, Rampley GJ, Neilson ET, Carroll AL (2008). Mountain pine beetle and forest carbon feedback to climate change. Nature..

[18] Raffa KF, Andersson MN, Schlyter F (2016). Chapter one-host selection by bark beetles: playing the odds in a high-stakes game. Adv Insect Physiol.

[19] Biedermann PHW, Grégoire J-C, Gruppe A, Hagge J, Hammerbacher A, Hofstetter R (2018). Bark beetle population dynamics in the Anthropocene: challenges and solutions. Trends Ecol Evol.

[20] Wermelinger B. Ecology and management of the spruce bark beetle *Ips typographus*—a review of recent research. For Ecol Manag. 2004;202:67–82.

[21] Bakke A, Frøyen P, Skattebøl LJN. Field response to a new pheromonal compound isolated from *Ips typographus*. Naturwissenschaften. 1977;64:98–9.

[22] Birgersson G, Schlyter F, Löfqvist J, Bergström G. Quantitative variation of pheromone components in the spruce bark beetle *Ips typographus* from different attack phases. J Chem Ecol. 1984;10:1029–55. 10.1007/BF00987511 24318847

[23] Schlyter F, Birgersson G, Byers JA, Löfqvist J, Bergström G. Field response of spruce bark beetle, *Ips typographus*, to aggregation pheromone candidates. J Chem Ecol. 1987;13:701–16. 10.1007/BF01020153 24302039

[24] Francke W, Sauerwein P, Vité JP, Klimetzek D. The pheromone bouquet of *Ips amitinus*. Naturwissenschaften. 1980;67:147–8.

[25] Schlyter F, Birgersson G, Leufvén A (1989). Inhibition of attraction to aggregation pheromone by verbenone and ipsenol. J Chem Ecol.

[26] Binyameen M, Jankuvová J, Blaženec M, Jakuš R, Song L, Schlyter F (2014). Co-localization of insect olfactory sensory cells improves the discrimination of closely separated odour sources. Funct Ecol.

[27] Unelius RC, Schiebe C, Bohman B, Andersson MN, Schlyter F. Non-host volatile blend optimization for forest protection against the European spruce bark beetle, *Ips typographus*. Plos One. 2014;9:e85381. 10.1371/journal.pone.0085381 PMC3891852 24454855

[28] Byers J. Avoidance of competition by spruce bark beetles,* Ips typographu*s and *Pityogenes chalcographus*. Experientia. 1993;49:272–5.

[29] Andersson MN, Larsson MC, Schlyter F. Specificity and redundancy in the olfactory system of the bark beetle *Ips typographus*: single-cell responses to ecologically relevant odors. J Insect Physiol. 2009;55:556–67. 10.1016/j.jinsphys.2009.01.018 19233334

[30] Tømmerås BÅ. Specialization of the olfactory receptor cells in the bark beetle *Ips typographus* and its predator *Thanasimus formicarius* to bark beetle pheromones and host tree volatiles. J Comp Physiol A. 1985;157:335–42.

[31] Schiebe C, Unelius CR, Ganji S, Binyameen M, Birgersson G, Schlyter F. Styrene, (+)-*trans*-(1*R*,4*S*,5*S*)-4-thujanol and oxygenated monoterpenes related to host stress elicit strong electrophysiological responses in the bark beetle *Ips typographus*. J Chem Ecol. 2019;45:474–89. 10.1007/s10886-019-01070-8 PMC6570694 31053976

[32] Mustaparta H, Tømmerås BA, Baeckström P, Bakke JM, Ohloff G (1984). Ipsdienol-specific receptor cells in bark beetles: structure-activity relationships of various analogues and of deuterium-labelled ipsdienol. J Comp Physiol A.

[33] Tømmerås BA, Mustaparta H, Gregoire J-C. Receptor cells in *Ips typographus* and *Dendroctonus micans* specific to pheromones of the reciprocal genus. J Chem Ecol. 1984;10:759–70. 10.1007/BF00988541 24318738

[34] de Fouchier A, Walker WB, Montagné N, Steiner C, Binyameen M, Schlyter F (2017). Functional evolution of Lepidoptera olfactory receptors revealed by deorphanization of a moth repertoire. Nat Commun.

[35] Große-Wilde E, Gohl T, Bouché E, Breer H, Krieger J (2007). Candidate pheromone receptors provide the basis for the response of distinct antennal neurons to pheromonal compounds. Eur J Neurosci.

[36] Yuvaraj JK, Andersson MN, Corcoran JA, Anderbrant O, Löfstedt C. Functional characterization of odorant receptors from *Lampronia capitella* suggests a non-ditrysian origin of the lepidopteran pheromone receptor clade. Insect Biochem Mol Biol. 2018;100:39–47. 10.1016/j.ibmb.2018.06.002 29894821

[37] Carey AF, Wang G, Su C-Y, Zwiebel LJ, Carlson JR. Odorant reception in the malaria mosquito *Anopheles gambiae*. Nature. 2010;464:66–71. 10.1038/nature08834 PMC2833235 20130575

[38] Hallem EA, Carlson JR (2006). Coding of odors by a receptor repertoire. Cell..

[39] Mitchell RF, Hughes DT, Luetje CW, Millar JG, Soriano-Agatón F, Hanks LM, et al. Sequencing and characterizing odorant receptors of the cerambycid beetle *Megacyllene caryae*. Insect Biochem Mol Biol. 2012;42:499–505. 10.1016/j.ibmb.2012.03.007 PMC3361640 22504490

[40] Wang X, Wang S, Yi J, Li Y, Liu J, Wang J, et al. Three host plant volatiles, hexanal, lauric acid, and tetradecane, are detected by an antenna-biased expressed odorant receptor 27 in the dark black chafer *Holotrichia parallela*. J Agric Food Chem. 2020;68:7316–23. 10.1021/acs.jafc.0c00333 32551589

[41] Antony B, Johny J, Montagné N, Jacquin-Joly E, Capoduro R, Cali K et al. Pheromone receptor of the globally invasive quarantine pest of the palm tree, the red palm weevil (*Rhynchophorus ferrugineus*). bioRxiv preprint. 2020; doi: 10.1101/2020.07.31.230326. 10.1111/mec.15874 33687767

[42] Mitchell RF, Andersson MN, Blomquist GJ, Vogt RG (2020). Olfactory genomics of the Coleoptera. Insect pheromone biochemistry and molecular biology.

[43] Andersson MN, Grosse-Wilde E, Keeling CI, Bengtsson JM, Yuen MM, Li M, et al. Antennal transcriptome analysis of the chemosensory gene families in the tree killing bark beetles,* Ips typographu*s and *Dendroctonus ponderosae* (Coleoptera: Curculionidae: Scolytinae). BMC Genomics. 2013;14:198. 10.1186/1471-2164-14-198 PMC3610139 23517120

[44] Mitchell RF, Schneider TM, Schwartz AM, Andersson MN, McKenna DD (2020). The diversity and evolution of odorant receptors in beetles (Coleoptera). Insect Mol Biol.

[45] Andersson MN, Keeling CI, Mitchell RF. Genomic content of chemosensory genes correlates with host range in wood-boring beetles (*Dendroctonus ponderosae, Agrilus planipennis, and Anoplophora glabripennis*). BMC Genomics. 2019;20:690. 10.1186/s12864-019-6054-x PMC6720082 31477011

[46] Corcoran JA, Jordan MD, Carraher C, Newcomb RD (2014). A novel method to study insect olfactory receptor function using HEK293 cells. Insect Biochem Mol Biol.

[47] Hou X, Zhang D-D, Yuvaraj JK, Corcoran JA, Andersson MN, Löfstedt C. Functional characterization of odorant receptors from the moth *Eriocrania semipurpurella*: a comparison of results in the* Xenopus* oocyte and HEK cell systems. Insect Biochem Mol Biol. 2020;117:103289. 10.1016/j.ibmb.2019.103289 31778795

[48] Brown HC, Randad RS (1990). Chiral synthesis VIA organoboranes. 26. An efficient synthesis of isoprenyl derivatives of borane-valuable reagents for the isoprenylboration of aldehydes. A convenient route to both enantiomers of ipsenol and ipsdienol in high optical purity. Tetrahedron..

[49] Erver F, Hilt G (2011). Multi-component regio-and diastereoselective cobalt-catalyzed hydrovinylation/allylboration reaction sequence. Org Lett.

[50] Klusener PAA, Hommes HH, Verkruijsse HD, Brandsma L (1985). Direct metallation of isoprene. J Chem Soc Chem Comm.

[51] Nemoto H (1994). A new alkenyl ether giving acetal with stereospecific manner. Tetrahedron Lett.

[52] Nemoto H, Zhong W, Kawamura T, Kamiya M, Nakano Y, Sakamoto K (2007). Synthesis of pptically active δ-dodecalactone via chiral resolution using CPF. Synlett..

[53] Corcoran JA, Sonntag Y, Andersson MN, Johanson U, Löfstedt C. Endogenous insensitivity to the Orco agonist VUAA1 reveals novel olfactory receptor complex properties in the specialist fly *Mayetiola destructor*. Sci Rep. 2018;8:3489. 10.1038/s41598-018-21631-3 PMC5823858 29472565

[54] Hopf TA, Morinaga S, Ihara S, Touhara K, Marks DS, Benton R (2015). Amino acid coevolution reveals three-dimensional structure and functional domains of insect odorant receptors. Nat Commun.

[55] Pellegrino M, Steinbach N, Stensmyr MC, Hansson BS, Vosshall LB (2011). A natural polymorphism alters odour and DEET sensitivity in an insect odorant receptor. Nature..

[56] Leary GP, Allen JE, Bunger PL, Luginbill JB, Linn CE, Macallister IE (2012). Single mutation to a sex pheromone receptor provides adaptive specificity between closely related moth species. Proc Natl Acad Sci U S A.

[57] Nichols AS, Luetje CW. Transmembrane segment 3 of *Drosophila melanogaster* odorant receptor subunit 85b contributes to ligand-receptor interactions. J Biol Chem. 2010;285:11854–62. 10.1074/jbc.M109.058321 PMC2852922 20147286

[58] Hallberg E. Sensory organs in *Ips typographus* (Insecta: Coleoptera) - fine structure of antennal sensilla. Protoplasma. 1982;111:206–14.

[59] Jones PL, Pask GM, Rinker DC, Zwiebel LJ (2011). Functional agonism of insect odorant receptor ion channels. Proc Natl Acad Sci U S A.

[60] Gu X-C, Zhang Y-N, Kang K, Dong S-L, Zhang L-W. Antennal transcriptome analysis of odorant reception genes in the red turpentine beetle (RTB), *Dendroctonus valens* Plos One 2015;10:e0125159. 10.1371/journal.pone.0125159 PMC4418697 25938508

[61] Antony B, Soffan A, Jakše J, Abdelazim MM, Aldosari SA, Aldawood AS, et al. Identification of the genes involved in odorant reception and detection in the palm weevil *Rhynchophorus ferrugineu*s, an important quarantine pest, by antennal transcriptome analysis. BMC Genomics. 2016;17:69. 10.1186/s12864-016-2362-6 PMC4722740 26800671

[62] Hunt T, Bergsten J, Levkanicova Z, Papadopoulou A, St. John O, Wild R (2007). A comprehensive phylogeny of beetles reveals the evolutionary origins of a superradiation. Science..

[63] Larsson MC, Leal WS, Hansson BS. Olfactory receptor neurons detecting plant odours and male volatiles in *Anomala cuprea* beetles (Coleoptera: Scarabaeidae). J Insect Physiol. 2001;47:1065–76. 10.1016/s0022-1910(01)00087-7 11472769

[64] Miazzi F, Schulze H-C, Zhang L, Kaltofen S, Hansson BS, Wicher D (2018). Low Ca2+ levels in the culture media support the heterologous expression of insect odorant receptor proteins in HEK cells. J Neurosci Methods.

[65] Andersson MN, Corcoran JA, Zhang D-D, Hillbur Y, Newcomb RD, Löfstedt C. A sex pheromone receptor in the hessian fly *Mayetiola destructor* (Diptera, Cecidomyiidae). Front Cell Neurosci. 2016;10:212. 10.3389/fncel.2016.00212 PMC5013046 27656130

[66] Yuvaraj JK, Corcoran JA, Andersson MN, Newcomb RD, Anderbrant O, Löfstedt C. Characterization of odorant receptors from a non-ditrysian moth, *Eriocrania semipurpurella* sheds light on the origin of sex pheromone receptors in Lepidoptera. Mol Biol Evol. 2017;34:2733–46. 10.1093/molbev/msx215 PMC5850608 29126322

[67] Andersson MN. Mechanisms of odor coding in coniferous bark beetles: From neuron to behavior and application. Psyche J Entomol 2012;2012: Article ID 149572.

[68] Schlyter F, Birgersson GA, Hardie J, Minks AK (1999). Forest beetles. Pheromones of non-Lepidopteran insects associated with agricultural plants.

[69] Nichols AS, Chen S, Luetje CW (2011). Subunit contributions to insect olfactory receptor function: channel block and odorant recognition. Chem Senses.

[70] Kumar P, Wang Y, Zhang Z, Zhao Z, Cymes GD, Tajkhorshid E (2020). Cryo-EM structures of a lipid-sensitive pentameric ligand-gated ion channel embedded in a phosphatidylcholine-only bilayer. Proc Natl Acad Sci U S A.

[71] Turner SL, Li N, Guda T, Githure J, Cardé RT, Ray A (2011). Ultra-prolonged activation of CO2-sensing neurons disorients mosquitoes. Nature..

[72] Grabherr MG, Haas BJ, Yassour M, Levin JZ, Thompson DA, Amit I (2011). Full-length transcriptome assembly from RNA-Seq data without a reference genome. Nat Biotechnol.

[73] Waterhouse RM, Seppey M, Simão FA, Manni M, Ioannidis P, Klioutchnikov G (2017). BUSCO applications from quality assessments to gene prediction and phylogenomics. Mol Biol Evol.

[74] McKenna DD, Scully ED, Pauchet Y, Hoover K, Kirsch R, Geib SM, et al. Genome of the Asian longhorned beetle (*Anoplophora glabripennis*), a globally significant invasive species, reveals key functional and evolutionary innovations at the beetle–plant interface. Genome Biol. 2016;17:227. 10.1186/s13059-016-1088-8 PMC5105290 27832824

[75] Schoville SD, Chen YH, Andersson MN, Benoit JB, Bhandari A, Bowsher JH, et al. A model species for agricultural pest genomics: the genome of the Colorado potato beetle, *Leptinotarsa decemlineata* (Coleoptera: Chrysomelidae). Sci Rep. 2018;8:1931. 10.1038/s41598-018-20154-1 PMC5792627 29386578

[76] Haas BJ, Papanicolaou A, Yassour M, Grabherr M, Blood PD, Bowden J, et al. De novo transcript sequence reconstruction from RNA-seq using the trinity platform for reference generation and analysis. Nat Protoc. 2013;8:1494–512. 10.1038/nprot.2013.084 PMC3875132 23845962

[77] Katoh K, Misawa K, Ki K, Miyata T (2002). MAFFT: a novel method for rapid multiple sequence alignment based on fast Fourier transform. Nucleic Acids Res.

[78] Capella-Gutiérrez S, Silla-Martínez JM, Gabaldón T (2009). trimAl: a tool for automated alignment trimming in large-scale phylogenetic analyses. Bioinformatics.

[79] Lanfear R, Frandsen PB, Wright AM, Senfeld T, Calcott B (2016). PartitionFinder 2: new methods for selecting partitioned models of evolution for molecular and morphological phylogenetic analyses. Mol Biol Evol.

[80] Stamatakis A (2014). RAxML version 8: a tool for phylogenetic analysis and post-analysis of large phylogenies. Bioinformatics..

[81] Miller MA, Pfeiffer W, Schwartz T (2010). Creating the CIPRES Science Gateway for inference of large phylogenetic trees. 2010 gateway computing environments workshop (GCE): 14 Nov.

[82] Zhang D-D, Löfstedt C. Functional evolution of a multigene family: orthologous and paralogous pheromone receptor genes in the turnip moth, *Agrotis segetum*. Plos One. 2013;8:e77345. 10.1371/journal.pone.0077345 PMC3795068 24130875

[83] Krieger J, Grosse-Wilde E, Gohl T, Dewer Y, Raming K, Breer H. Genes encoding candidate pheromone receptors in a moth (*Heliothis virescens*). Proc Natl Acad Sci U S A. 2004;101:11845–50. 10.1073/pnas.0403052101 PMC511062 15289611

[84] Schultze A, Pregitzer P, Walter MF, Woods DF, Marinotti O, Breer H, et al. The co-expression pattern of odorant binding proteins and olfactory receptors identify distinct trichoid sensilla on the antenna of the malaria mosquito *Anopheles gambiae*. Plos One. 2013;8:e69412. 10.1371/journal.pone.0069412 PMC3702612 23861970

[85] Zhang D-D, Wang H-L, Schultze A, Froß H, Francke W, Krieger J, et al. Receptor for detection of a type II sex pheromone in the winter moth *Operophtera brumata*. Sci Rep. 2016;6:18576. 10.1038/srep18576 PMC4700456 26729427

[86] Waterhouse A, Bertoni M, Bienert S, Studer G, Tauriello G, Gumienny R (2018). SWISS-MODEL: homology modelling of protein structures and complexes. Nucleic Acids Res.

[87] Hildebrand PW, Goede A, Bauer RA, Gruening B, Ismer J, Michalsky E (2009). SuperLooper—a prediction server for the modeling of loops in globular and membrane proteins. Nucleic Acids Res.

[88] Phillips JC, Braun R, Wang W, Gumbart J, Tajkhorshid E, Villa E (2005). Scalable molecular dynamics with NAMD. J Comput Chem.

[89] Trott O, Olson AJ (2010). AutoDock Vina: improving the speed and accuracy of docking with a new scoring function, efficient optimization, and multithreading. J Comput Chem.

[90] Yuvaraj JK, Roberts RE, Sonntag Y, Hou X-Q, Grosse-Wilde E, Machara A, Zhang D-D, Hansson BS, Johanson U, Löfstedt C, Andersson MN. Putative ligand binding sites of two functionally characterized bark beetle odorant receptors. *NCBI accession* PRJNA602798, https://www.ncbi.nlm.nih.gov/bioproject/PRJNA602798. Accessed 6 Oct 2020. 10.1186/s12915-020-00946-6 PMC7836466 33499862

[91] Yuvaraj JK, Roberts RE, Sonntag Y, Hou X-Q, Grosse-Wilde E, Machara A, Hansson BS, Johanson U, Löfstedt C, Andersson MN: Functional characterization of two bark beetle odorant receptors and their putative ligand binding site. *GenBank accession* MN987209-MN987211, https://www.ncbi.nlm.nih.gov/nuccore/MN987209. Accessed 20 Oct 2020.

