# Supplementary material for: Putative ligand binding sites of two functionally characterized bark beetle odorant receptors
Source: BMC Biol. 2021 Jan 26;19:16. doi: 10.1186/s12915-020-00946-6 (PMC7836466; doi:10.1186/s12915-020-00946-6)
Supplement: Supplementary file 2 — Additional file 2: Supplementary Table 2. Compounds used for characterization of Ips typographus odorant receptors (ORs) and Orco, including their purities, source information, and examples of main biological origins. Supplementary Table 3. Assessment of the completeness of antennal transcriptome Trinity and CLC assemblies using the Benchmarking Universal Single-Copy Orthologs (BUSCOv3) tool performed against the Insecta odb9 dataset (https://busco.ezlab.org/).Supplementary Figure 1. Protein detection of Ips typographus odorant receptors (ORs; V5-tagged) and Orco (myc-tagged) from TREx/HEK293 cells by Western blot. Upper left panel: Detection of wildtype ItypOR46 and ItypOR49 (two cell lines). Upper right panel: detection of Orco in the same cell lines. Lower panel: detection of three versions of mutated ItypOR46 proteins and wildtype (WT) ItypOR46 (included as control). Proteins were only detected from cells induced (+) to express ItypORs and Orco, and not from non-induced (−) control cells, indicating proper regulation by the repression system. Supplementary Figure 2. Response of TREx/HEK293 cells expressing ItypOR46 and ItypOrco to all stimuli (30 μM) and vehicle control in the screening experiment (n = 3 biological replicates, each including 3 technical replicates, i.e., ntotal = 9). (+)-Induction: response of cells induced to express ItypOrco and ItypOR46; (−)-Induction: response of non-induced control cells. Data represent mean responses ± SEM. Supplementary Figure 3. Response of TREx/HEK293 cells expressing ItypOR49 and ItypOrco to all stimuli (30 μM) and vehicle control in the screening experiment (n = 3 biological replicates, each including 3 technical replicates, i.e., ntotal = 9). (+)-Induction: response of cells induced to express ItypOrco and ItypOR49; (−)-Induction: response of non-induced control cells. Data represent mean responses ± SEM. Supplementary Figure 4. A) Current traces of two oocytes expressing ItypOrco/ItypOR49, indicating responses to th [file 12915_2020_946_MOESM2_ESM.pdf]

Additional file 2

Putative ligand binding sites of two functionally characterized bark beetle odorant receptors

Jothi K. Yuvaraj, Rebecca E. Roberts, Yonathan Sonntag, Xiao-Qing Hou, Ewald Grosse-Wilde, Aleš Machara, Dan-Dan Zhang, Bill S. Hansson, Urban Johanson, Christer Löfstedt, and Martin N. Andersson

Material included

Supplementary Table 2	S-2
Supplementary Table 3	S-3
Supplementary Figure 1	S-4
Supplementary Figure 2	S-5
Supplementary Figure 3	S-6
Supplementary Figure 4	S-7
Supplementary Figure 5	S-7
Supplementary Figure 6	S-8
Supplementary Figure 7	S-9
Supplementary Methods	S-12
-Chemistry	S-12
-Experimental procedures for preparation of pheromones	S-14
-GC analysis with chiral column	S-17
-Copies of NMR spectra	S-20
-References	S-25
List of additional supplementary files	S-25

Supplementary Table 2. Compounds used for characterization of *Ips typographus* odorant receptors (ORs) and Orco, including their purities, source information, and examples of main biological origins.

Compound	Purity (%)*	Source*	Examples of biological origin
Acetophenone	99	Acros	Beetle, fungi
Amitinol	91	R. U.	Beetle
Anisole	>99	Sigma-Aldrich	Fungi
Benzaldehyde	>99	Kebo	Non-host, fungi
Benzyl acetate	>99	Aldrich	Fungi
Benzyl alcohol	99	Aldrich	Non-host, fungi
(±)-*exo*-Brevicomin	99	W. F.	Beetle, fungi
(±)-*endo*-Brevicomin	96	Synergy Semiochemicals	Beetle, fungi
(±)-Camphene	95	Aldrich	Host
(±)-Camphor	97	Aldrich	Host, fungi
(+)-3-Carene	99	Aldrich	Host
(±)-Carvone	>99	Fluka	Fungi
(±)-Chalcogran	90	Celamerck, GmbH	Beetle
1,8-Cineole	99	Aldrich	Host
Citral (*E*/*Z* mix)	99	Aldrich	Fungi
(5*S*,7*S*)-*trans*-Conophthorin	94	W. F.	Non-host, fungi
p-Cymene	>99	Acros	Host
2,3-Dihydrobenzofuran	99	Aldrich	Fungi
3,4-Dimethoxytoluene	98	Givaudan-Roure	Host, fungi
Estragole (4-allylanisole)	>99	Aldrich	Host, fungi
4-Ethylguaiacol	98	Sigma-Aldrich	Fungi
Eugenol methyl ether	>99	Fluka	Host, fungi
(±)-Frontalin	>99	Synergy Semiochemicals	Beetle
Geranyl acetate	97	Aldrich	Fungi
Geranylacetone	>99	Fluka	Non-host, fungi
Hexanal	96	Sigma	Non-host
1-hexanol	>99	Fluka	Non-host, fungi
*E*2-hexenal	98	Aldrich	Non-host
*E*2-hexenol	96	Aldrich	Non-host
*E*3-hexenol	98	Aldrich	Non-host
*Z*2-hexenol	95	Aldrich	Non-host
*Z*3-hexenol	98	Aldrich	Non-host
(±)-Ipsdienol	94	Bedoukian	Beetle
(*R*)-(-)-Ipsdienol	99 (98% ee)	A. M.	Beetle
(*S*)-(+)-Ipsdienol	98 (98% ee)	A. M.	Beetle
(±)-Ipsenol	95	Synergy Semiochemicals	Beetle
(*R*)-(+)-Ipsenol	>99 (99% ee)	A. M.	Not produced by beetles
(*S*)-(-)-Ipsenol	>99 (98% ee)	A. M.	Beetle
(+)-Isopinocamphone	>99	R. U.	Host, fungi
(-)-Isopinocamphone	>99	R. U.	Host, fungi
Lanierone	>99	Synergy Semiochemicals	Beetle
(-)-Limonene	>99	Fluka	Host
4-Methyl anisole	>99	Fluka	Fungi
2-Methyl-1-butanol	>99	Aldrich	Fungi
3-Methyl-1-butanol	99	Aldrich	Fungi
2-Methyl-3-buten-2-ol	>99	Acros	Beetle, fungi
(±)-2-Methylbutyl acetate	>99	SAFC	Fungi
3-Methylbutyl acetate	97	Sigma-Aldrich	Fungi
Myrcene	95	Sigma-Aldrich	Host
E-Myrcenol	>99	Fytofarm	Beetle

(±)-Myrtenol	96	G. B.	Beetle, fungi
(±)-3-Octanol	97	Sigma-Aldrich	Non-host, fungi
(±)-1-Octen-3-ol	98	Janssen Chimica	Non-host, fungi
2-Phenethyl acetate	>99	Aldrich	Fungi
2-Phenylethanol	>99	Sigma	Beetle, fungi
(+)-α-Pinene	98	Janssen Chimica	Host
(-)-α-Pinene	>99	Fluka	Host
(-)-β-Pinene	92	Fluka	Host
(+)-Pinocamphone	84	R. U	Host, fungi
(-)-Pinocamphone	81	R. U.	Host, fungi
(-)-Pinocarvone	99	D. K.	Beetle, fungi
(±)-Sabinene	97	Chemos GmbH	Host, non-host
Styrene	>99	Fluka	Fungi
γ-Terpinene	97	Aldrich	Host
Terpinolene	98	Fluka	Host
(+)-*trans*-4-Thujanol	97	Sigma-Aldrich	Host, fungi
Toluene	>99	Merck	Fungi
(-)-*cis*-Verbenol	99	Borregaard	Beetle
(+)-*trans*-Verbenol	92	SCM	Beetle
(-)-*trans*-Verbenol	97	SciTech Ltd., Prague	Beetle
(-)-Verbenone	>99	Fluka	Beetle, fungi
4-Vinyl anisole	97	Aldrich	Fungi
VUAA1	98	Sigma-Aldrich	None

* Abbrevations: ee = enantiomeric excess; R. U. = gift from Rikard Unelius (Linnaeus University, Kalmar, Sweden); W. F. = gift from Wittko Francke (University of Hamburg, Germany); A. M. = synthesized in this study by Aleš Machara; G. B. = gift from Gunnar Bergström (University of Gothenburg, Sweden); D. K. = gift from Dineshkumar Kandasamy (Max Planck Institute for Chemical Ecology, Jena, Germany).

Supplementary Table 3. Assessment of the completeness of antennal transcriptome Trinity and CLC assemblies using the Benchmarking Universal Single-Copy Orthologs (BUSCOv3) tool performed against the Insecta odb9 dataset (https://busco.ezlab.org/).

	Trinity	CLC
Complete BUSCOs (C)	1615 (97.4%)	1328 (80.1%)
Complete and single-copy BUSCOs (S)	378	1284
Complete and duplicated BUSCOs (D)	1237	44
Fragmented BUSCOs (F)	27	230
Missing BUSCOs (M)	16	100
Total BUSCO groups searched	1658	1658

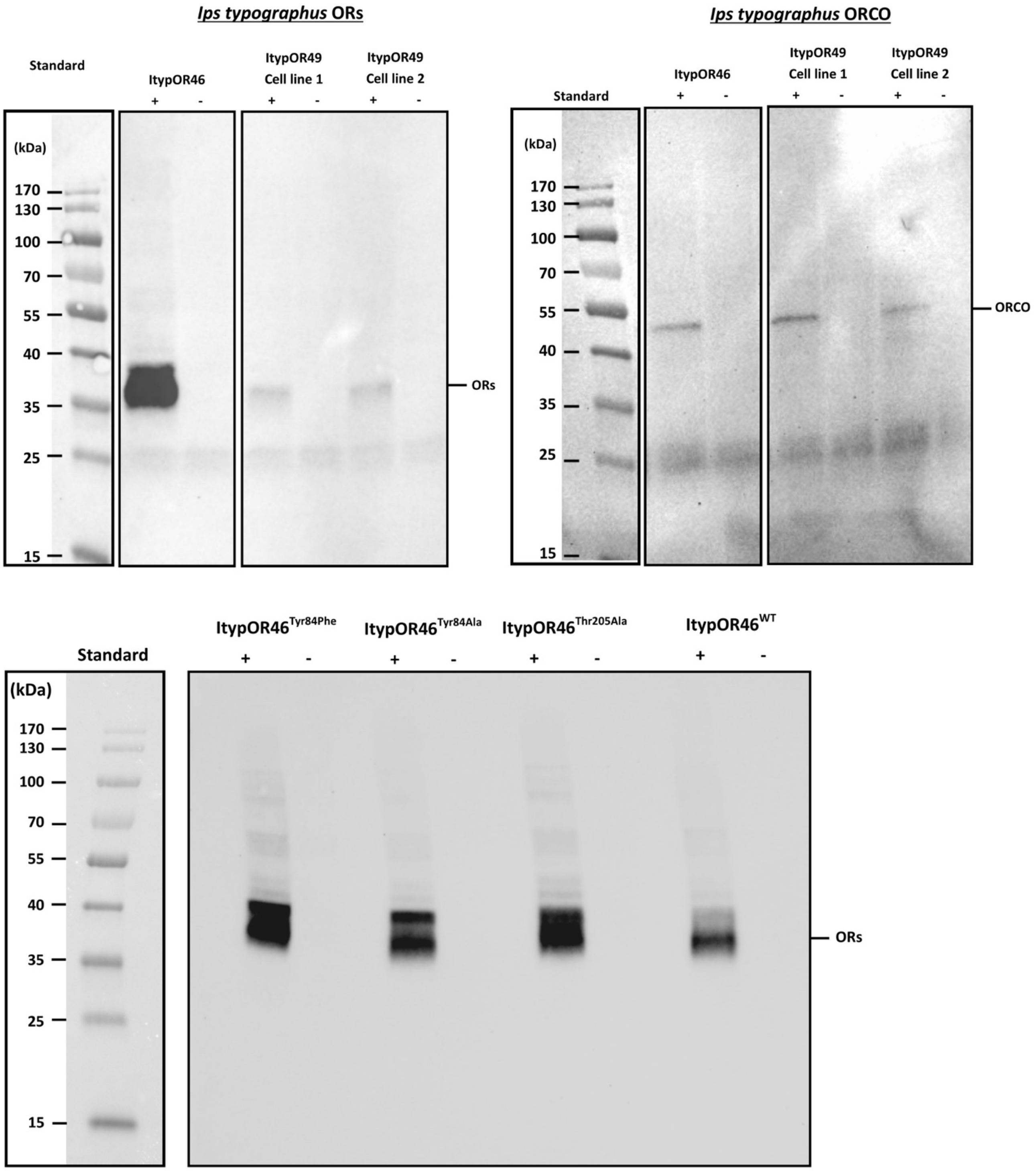

Supplementary Figure 1. Protein detection of *Ips typographus* odorant receptors (ORs; V5-tagged) and Orco (myc-tagged) from TREx/HEK293 cells by Western blot. *Upper left panel:* Detection of wildtype ItypOR46 and ItypOR49 (two cell lines). *Upper right panel:* detection of Orco in the same cell lines. *Lower panel:* detection of three versions of mutated ItypOR46 proteins and wildtype (WT) ItypOR46 (included as control). Proteins were only detected from cells induced (+) to express ItypORs and Orco, and not from non-induced (−) control cells, indicating proper regulation by the repression system.

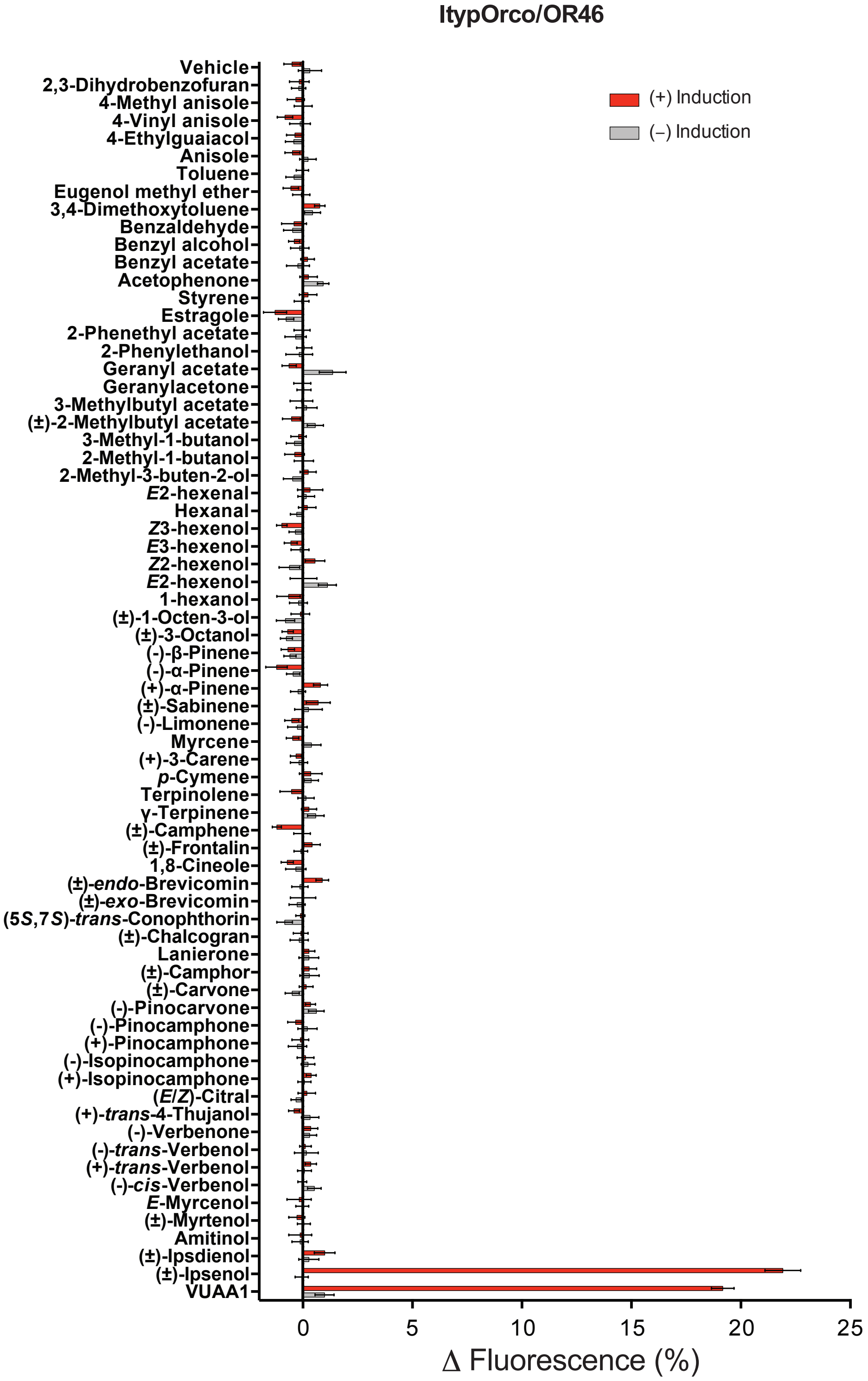

Supplementary Figure 2. Response of TREx/HEK293 cells expressing ItypOR46 and ItypOrco to all stimuli (30 μM) and vehicle control in the screening experiment (n = 3 biological replicates, each including 3 technical replicates, i.e., $n_{total} = 9$). (+)-Induction: response of cells induced to express ItypOrco and ItypOR46; (–)-Induction: response of non-induced control cells. Data represent mean responses ± SEM.

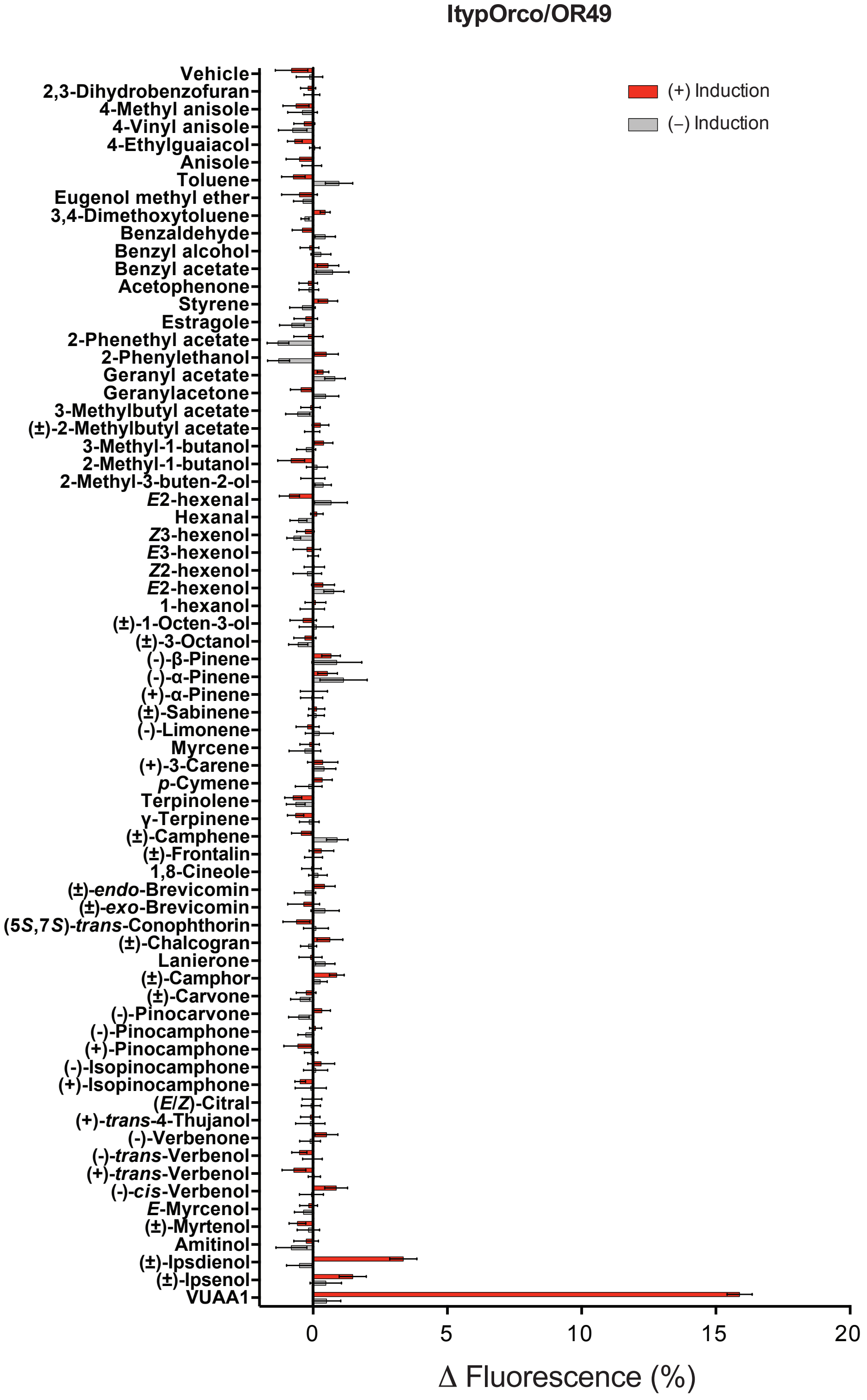

Supplementary Figure 3. Response of TREx/HEK293 cells expressing ItypOR49 and ItypOrco to all stimuli (30 µM) and vehicle control in the screening experiment (n = 3 biological replicates, each including 3 technical replicates, i.e., n_{total} = 9). (+)-Induction: response of cells induced to express ItypOrco and ItypOR49; (–)-Induction: response of non-induced control cells. Data represent mean responses ± SEM.

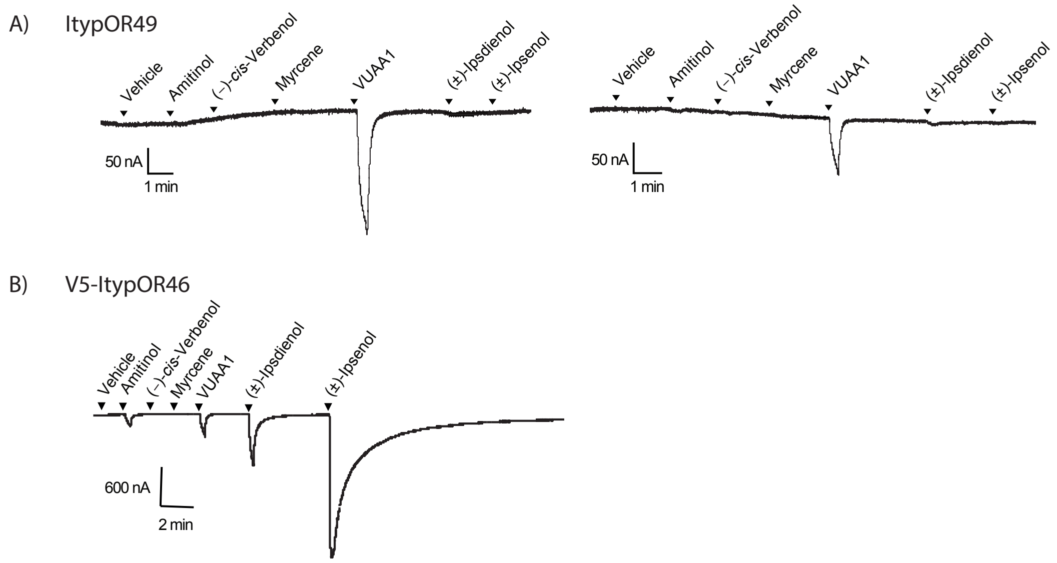

Supplementary Figure 4. A) Current traces of two oocytes expressing ItypOrco/ItypOR49, indicating responses to the Orco agonist VUAA1 and minute responses to racemic ipsdienol. **B)** Current trace of oocyte expressing myc-tagged ItypOrco and V5-tagged ItypOR46 showing the same specificity as non-tagged versions of these receptors in this system.

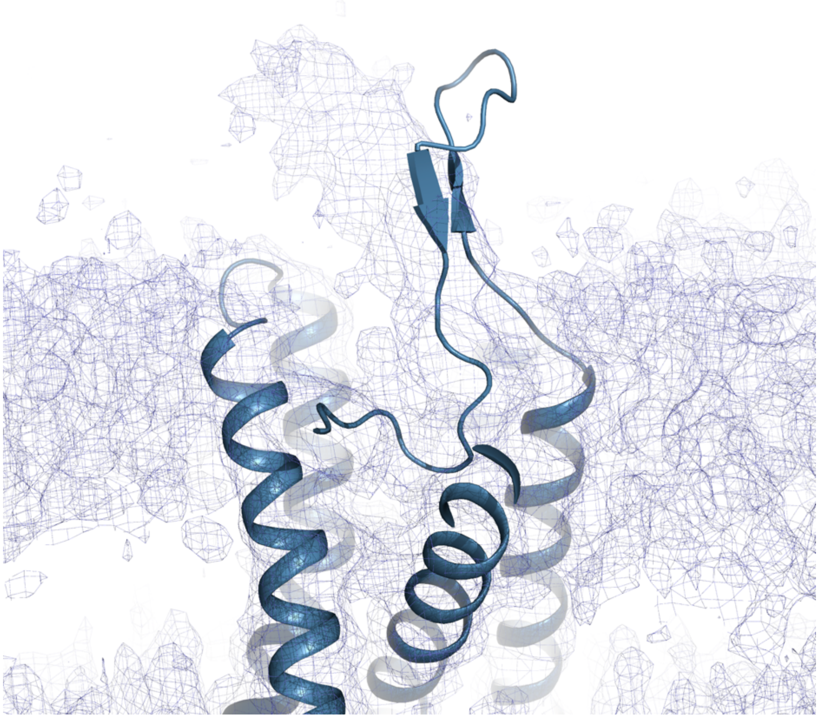

Supplementary Figure 5. Energy minimized model of ItypOR49 (blue cartoon) showing the extended extracellular loop 2 (EL2) overlaid with the published Orco Cryo-EM map (mesh). Homology models of ItypOR46 and ItypOR49 were built using the Orco structure (PDB ID 6c70) as template. The part of EL2 that was not included in the published structure (residues 156-170) was modelled *ab initio*. An extended β-sheet fold in this part of the loop is supported by the density in the Cryo-EM map. In neither of the models the putative binding cleft is blocked by this part of EL2. The RMSDs between the Cα of the template and energy minimized models for ItypOR46 and ItypOR49 (excluding the *ab initio* modelled parts) are 0.958 Å and 0.880 Å, respectively.

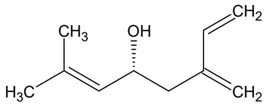

(*R*)-Ipsdienol
(4*R*)-2-methyl-6-methylideneocta-2,7-dien-4-ol

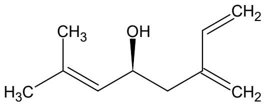

(*S*)-Ipsdienol
(4*S*)-2-methyl-6-methylideneocta-2,7-dien-4-ol

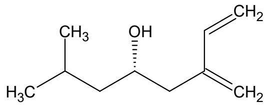

(*S*)-Ipsenol
(4*S*)-2-methyl-6-methylideneoct-7-en-4-ol

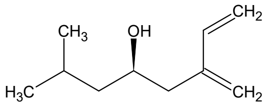

(*R*)-Ipsenol
(4*R*)-2-methyl-6-methylideneoct-7-en-4-ol

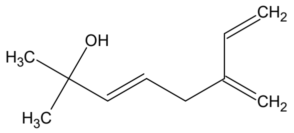

Amitinol
(3*E*)-2-methyl-6-methylideneocta-3,7-dien-2-ol

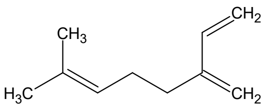

Myrcene
7-methyl-3-methylideneocta-1,6-diene

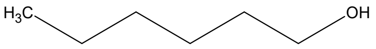

Hexan-1-ol

Supplementary Figure 6. Chemical structures and IUPAC names of compounds included in the molecular docking analyses against ItypOR46 and ItypOR49.

A)

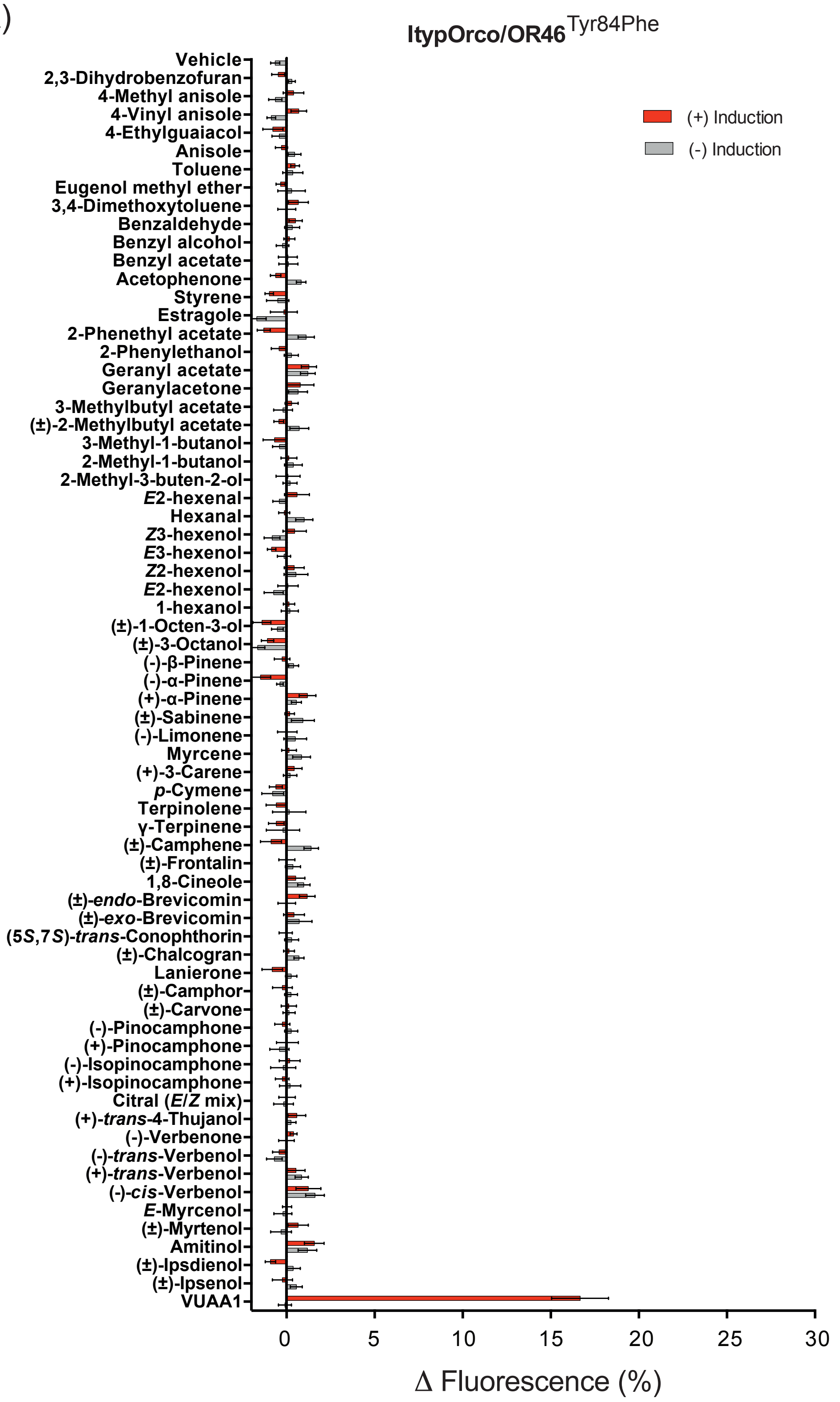
ItypOrco/OR46^{Tyr84Phe}
(+) Induction
(-) Induction
Vehicle
2,3-Dihydrobenzofuran
4-Methyl anisole
4-Vinyl anisole
4-Ethylguaiacol
Anisole
Toluene
Eugenol methyl ether
3,4-Dimethoxytoluene
Benzaldehyde
Benzyl alcohol
Benzyl acetate
Acetophenone
Styrene
Estragole
2-Phenethyl acetate
2-Phenylethanol
Geranyl acetate
Geranylacetone
3-Methylbutyl acetate
(±)-2-Methylbutyl acetate
3-Methyl-1-butanol
2-Methyl-1-butanol
2-Methyl-3-buten-2-ol
E2-hexenal
Hexanal
Z3-hexenol
E3-hexenol
Z2-hexenol
E2-hexenol
1-hexanol
(±)-1-Octen-3-ol
(±)-3-Octanol
(-)-β-Pinene
(-)-α-Pinene
(+)-α-Pinene
(±)-Sabinene
(-)-Limonene
Myrcene
(+)-3-Carene
p-Cymene
Terpinolene
γ-Terpinene
(±)-Camphene
(±)-Frontalin
1,8-Cineole
(±)-endo-Brevicomin
(±)-exo-Brevicomin
(5S,7S)-trans-Conophthorin
(±)-Chalcogran
Lanierone
(±)-Camphor
(±)-Carvone
(-)-Pinocamphone
(+)-Pinocamphone
(-)-Isopinocamphone
(+)-Isopinocamphone
Citral (E/Z mix)
(+)-trans-4-Thujanol
(-)-Verbenone
(-)-trans-Verbenol
(+)-trans-Verbenol
(-)-cis-Verbenol
E-Myrcenol
(±)-Myrtenol
Amitinol
(±)-Ipsdienol
(±)-Ipsenol
VUAA1
0
5
10
15
20
25
30
Δ Fluorescence (%)

B)

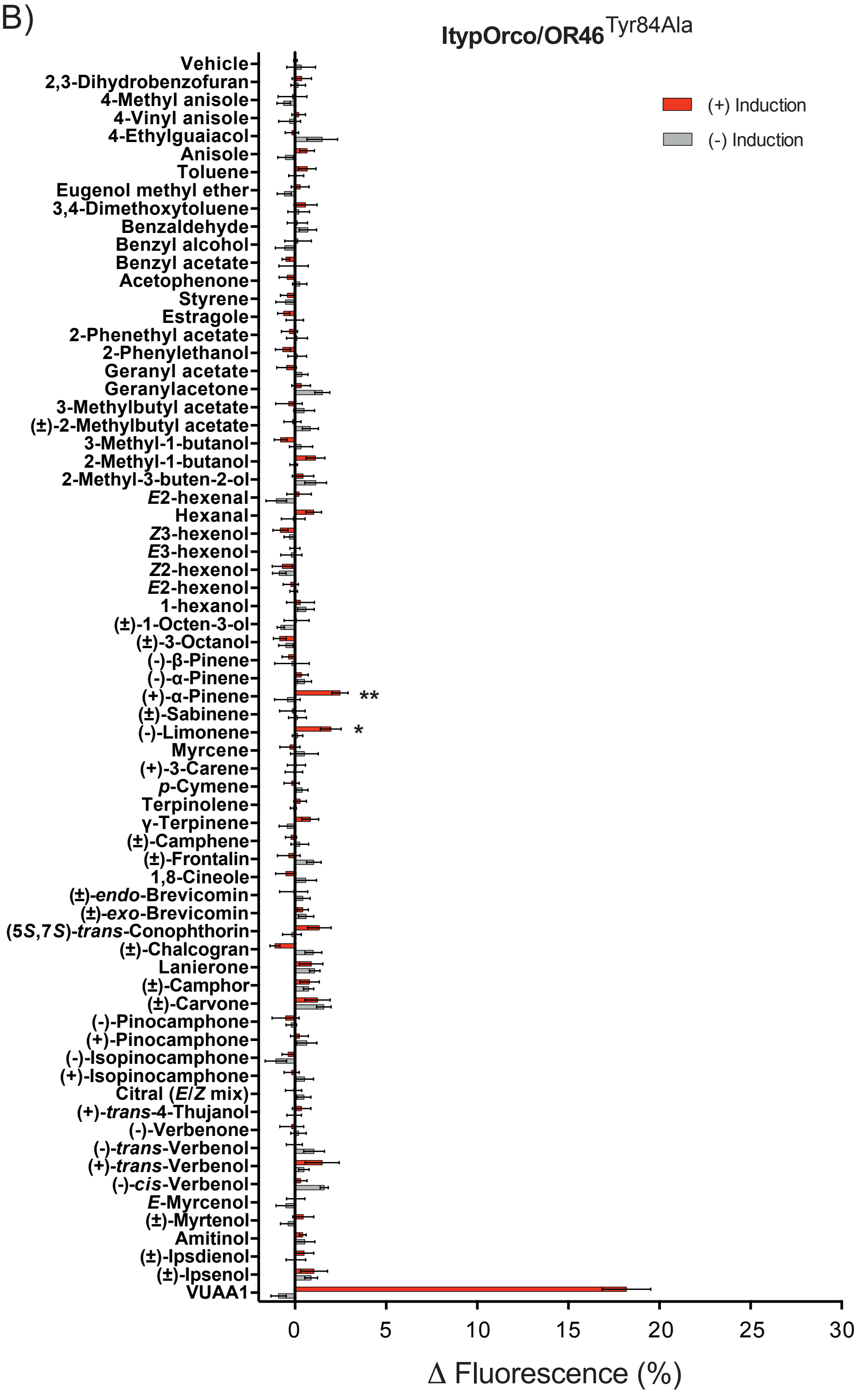
ItypOrco/OR46^Tyr84Ala
(+) Induction
(-) Induction
Vehicle
2,3-Dihydrobenzofuran
4-Methyl anisole
4-Vinyl anisole
4-Ethylguaiacol
Anisole
Toluene
Eugenol methyl ether
3,4-Dimethoxytoluene
Benzaldehyde
Benzyl alcohol
Benzyl acetate
Acetophenone
Styrene
Estragole
2-Phenethyl acetate
2-Phenylethanol
Geranyl acetate
Geranylacetone
3-Methylbutyl acetate
(±)-2-Methylbutyl acetate
3-Methyl-1-butanol
2-Methyl-1-butanol
2-Methyl-3-buten-2-ol
E2-hexenal
Hexanal
Z3-hexenol
E3-hexenol
Z2-hexenol
E2-hexenol
1-hexanol
(±)-1-Octen-3-ol
(±)-3-Octanol
(-)-β-Pinene
(-)-α-Pinene
(+)-α-Pinene
**
(±)-Sabinene
(-)-Limonene
*
Myrcene
(+)-3-Carene
p-Cymene
Terpinolene
γ-Terpinene
(±)-Camphene
(±)-Frontalin
1,8-Cineole
(±)-endo-Brevicomin
(±)-exo-Brevicomin
(5S,7S)-trans-Conophthorin
(±)-Chalcogran
Lanierone
(±)-Camphor
(±)-Carvone
(-)-Pinocamphone
(+)-Pinocamphone
(-)-Isopinocamphone
(+)-Isopinocamphone
Citral (E/Z mix)
(+)-trans-4-Thujanol
(-)-Verbenone
(-)-trans-Verbenol
(+)-trans-Verbenol
(-)-cis-Verbenol
E-Myrcenol
(±)-Myrtenol
Amitinol
(±)-Ipsdienol
(±)-Ipsenol
VUAA1
0
5
10
15
20
25
30
Δ Fluorescence (%)

C)

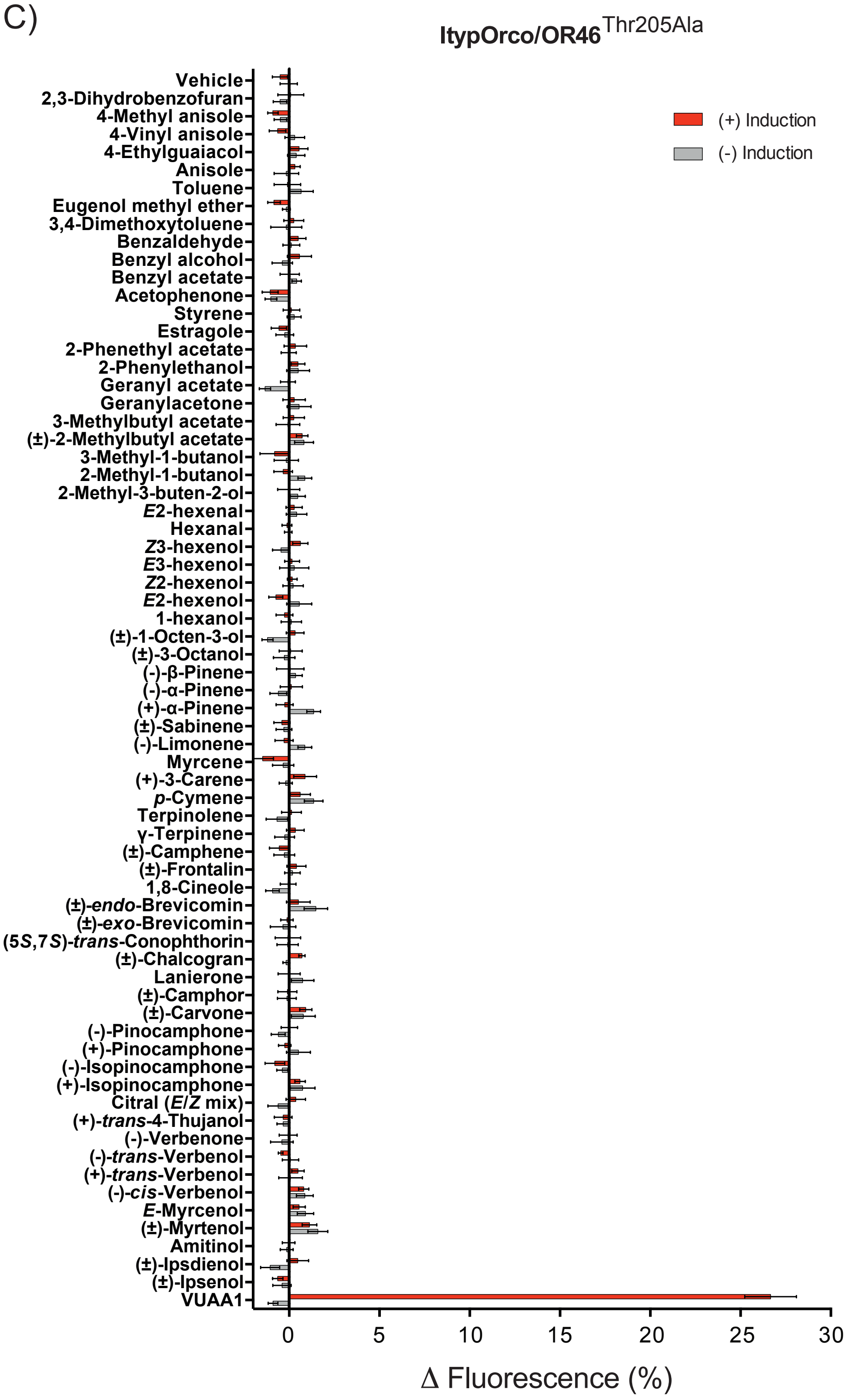

Supplementary Figure 7. Response of TREx/HEK293 cells expressing **(A)** ItypOR46^{Tyr84Phe}, **(B)** ItypOR46^{Tyr84Ala}, and **(C)** ItypOR46^{Thr205Ala} and ItypOrco to all stimuli (30 μM) and vehicle control in the screening experiment (n = 2 biological replicates, each including 3 technical replicates, i.e., n_{total} = 6). (+)-Induction: response of cells induced to express ItypOrco and the mutated ItypOR46; (−)-Induction: response of non-induced control cells. Data represent mean responses ± SEM. Asterisks (* $p < 0.05$; ** $p < 0.01$) in **(B)** indicate significant differences between induced and non-induced cells.

Supplementary Methods

Chemistry

Both enantiomers of ipsdienol were obtained by resolution of the corresponding racemate **1** (Scheme 1). The reaction of 2-methylbutenal with isoprenylpotassium[1,2] provided the mentioned racemate **1** that in turn was treated with chiral oxabicyclo[3.3.0]octene[3,4] to get a mixture of separable diastereomers **2a**,**b**. Purified diastereomers **2a** and **2b** were subjected to methanolysis under slightly acidic condition that furnished (*S*)-ipsdienol **1a** and (*R*)-ipsdienol **1b**.

Scheme 1.

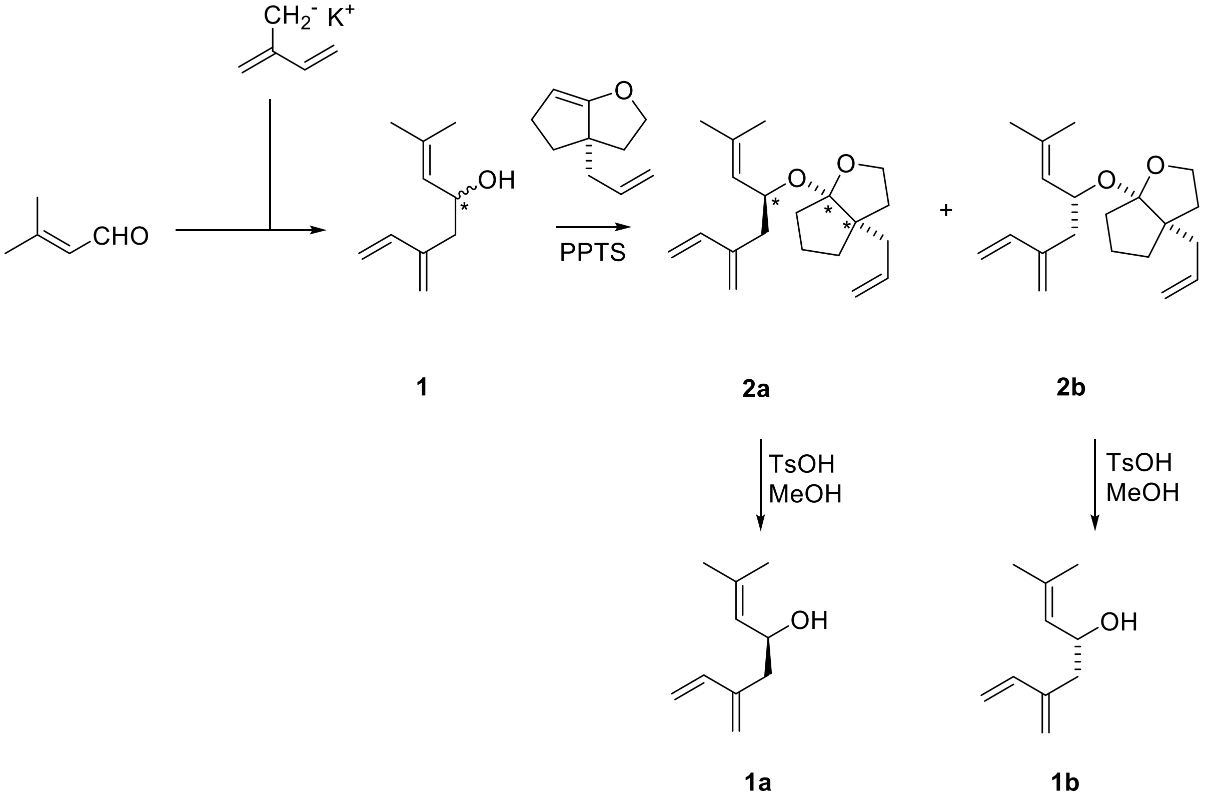

Both (*S*)- and (*R*)-ipsenol (**3a** and **3b**) were prepared with high enantioselectivity (>97% e.e.) following a slightly modified Brown's procedure (Scheme 2).[2] First, metallated isoprene was treated with commercially available chiral *B*-methoxy-diisopinocampheylborane (Ipc_2BOMe) to form the "ate" complex, followed by liberation of isoprenylboration reagent using $BF_3.Et_2O$. *In situ*-formed *B*-isoprenyl-diisopinocampheylborane (Ipc_2BIpn) was combined with isovaleraldehyde to provide a reaction mixture that was worked-up in a non-oxidative manner:

i.e. simple addition of neat acetaldehyde. The reaction mixture was then concentrated and subsequent column chromatography of the residue afforded ipsenols in good yields.

Scheme 2.

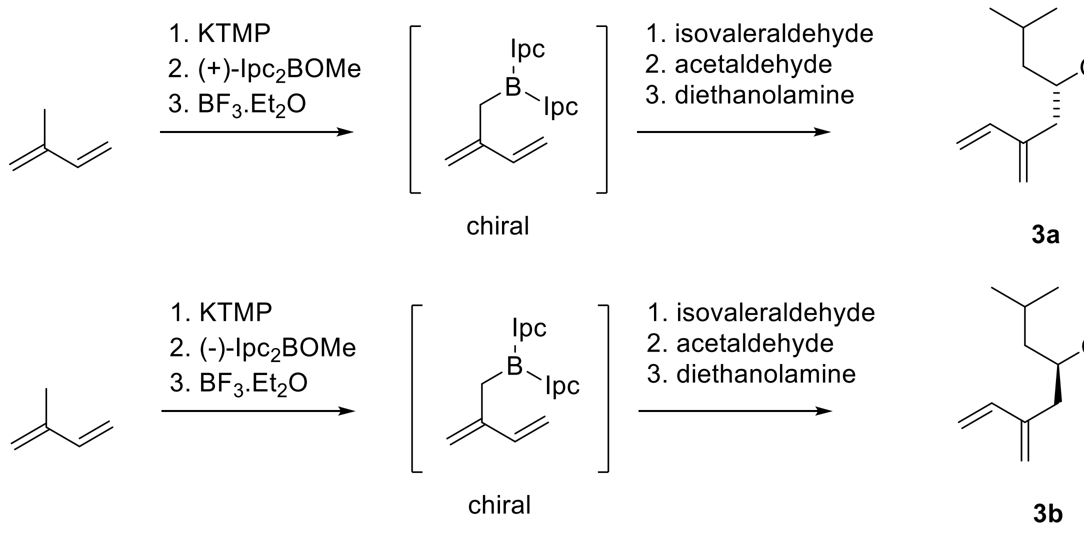

Preparation of racemic ipsenol **3** based on simple addition of isoprenylpotassium to isovaleraldehyde failed, and thus the racemate **3** was prepared in a stepwise fashion (Scheme 3). Isoprenylpotassium was allowed to react with isopropoxy-tetramethyldioxaborolane. The formed isoprenyl boronate **4** was isolated and purified.[5] Its reaction with isovaleraldehyde in toluene afforded racemic ipsenol **3**.

Scheme 3.

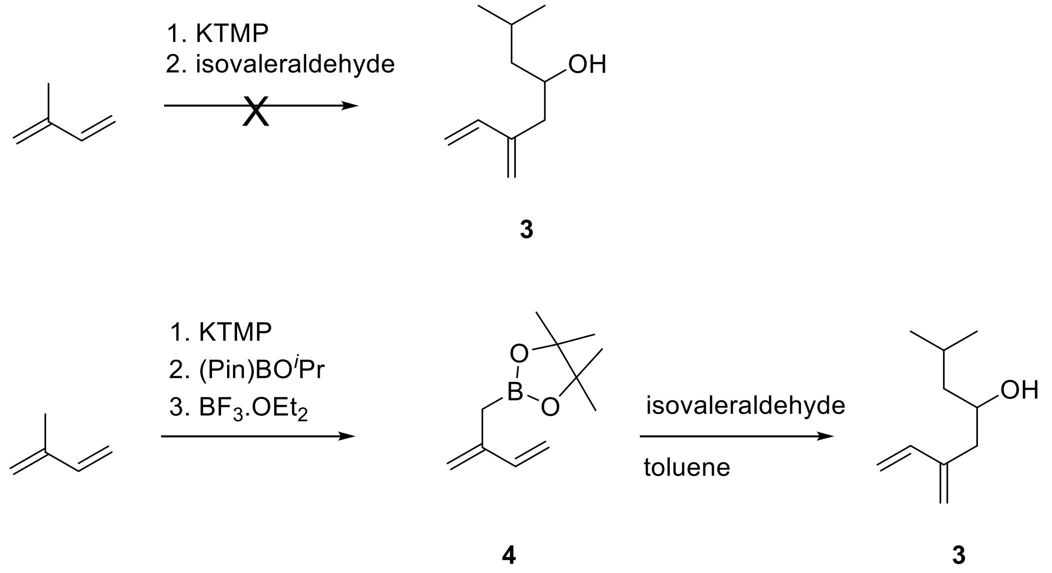

Experimental procedures

General: Unless otherwise noted, all reactions were carried out under argon using oven-dried glassware. The solvents used for reactions were distilled from drying agents indicated and were transferred under argon: THF (Na/benzophenone); toluene (Na). Chromatography was performed using Fluka silica gel 60 (0.040 - 0.063mm). For TLC analysis, F_{254} –coated aluminum sheets were used. The spots were detected both in UV and by the solution of $Ce(SO_4)_2{\cdot}4H_2O$ (1%) and $H_3P(Mo_3O_{10})_4$ (2%) in 10% sulfuric acid. All starting materials were used as purchased (Sigma Aldrich, TCI), unless otherwise noted. The ^{1}H-NMR spectra were measured at 400 MHz, the ^{13}C-NMR spectra at 100 MHz in $CDCl_3$ with tetramethylsilane or solvent peaks as an internal standard. The chemical shifts are given in δ-scale, coupling constants J are given in Hz. The EI mass spectra were determined at an ionizing voltage of 70 eV, the *m/z* values are given alone with their relative intensities (%). The ESI mass spectra were recorded using a ZQ micromass mass spectrometer (Waters) equipped with an ESCi multimode ion source and it was controlled by MassLynx software. Methanol was used as solvent.

Racemic ipsdienol **1**

The solution of butyllithium (5 mL, 2.5 M solution in hexane; 12.5 mmol) was added dropwise to a solution of tetramethylpiperidine (1.80 g; 12.5 mmol) in dry THF (5 mL) placed in an ice bath. After 15 min of stirring, the mixture was cooled to -78 °C. A solution of potassium *t*-butoxide (1.40 g; 12.5 mmol) in dry THF (8 mL) was added dropwise. Then solution of 3-methylbutenal (1.05 g; 12.5 mmol) was added slowly and the reaction mixture was removed from the cooling bath and was allowed to reach room temperature. After 30 min the reaction was quenched with a saturated solution of ammonium chloride. Crude product was extracted with diethyl ether. Combined organic layers were washed with water, brine, and concentrated under reduced pressure (120 Torr). Column chromatography (silica gel, eluent DCM) afforded 1.02 g (52%) of colorless liquid. ^{1}H NMR (400 MHz, $CDCl_3$) δ 6.60 – 6.23 (m, 1H), 5.45 – 4.96 (m, 5H), 4.54 (td, J = 8.4, 4.9 Hz, 1H), 2.63 – 2.27 (m, 2H), 1.76 (s, 3H), 1.73 (s, 3H).

Resolution of racemic ipsdienol, preparation of compounds **2a** and **2b**

A mixture of racemic ipsdienol (0.62 g; 4.07 mmol), (*S*)-5-allyl-2-oxabicyclo[3.3.0]oct-8-ene (0.73 g; 4.88 mmol) and catalytic amount of pyridinium *p*-toluenesulfonate (approx. 0.002 g)

in anhydrous DCM (5 mL) was stirred for 5 hours at room temperature. Afterwards the mixture was concentrated under reduced pressure. The obtained residue was subjected to thorough column chromatography (silica gel, eluent toluene: cyclohexane/2:3) that yielded two diastereomers. Diastereomer **2a**- 0.4 g (32%), $[\alpha]_D$ = -11.1° (c = 0.38, MeOH). ^{1}H NMR (400 MHz, $CDCl_3$) δ 6.38 (dd, *J* = 17.6, 10.8 Hz, 1H), 5.88 (ddt, *J* = 17.3, 10.1, 7.3 Hz, 1H), 5.36 (d, *J* = 17.6 Hz, 1H), 5.21 – 4.89 (m, 6H), 4.66 (dt, *J* = 9.1, 6.8 Hz, 1H), 3.92 – 3.58 (m, 2H), 2.51 (dd, *J* = 13.4, 7.1 Hz, 1H), 2.38 – 2.19 (m, 2H), 2.17 – 2.03 (m, 1H), 1.99 – 1.85 (m, 2H), 1.70 (m, 4H), 1.62 – 1.50 (m, 8H). ^{13}C NMR (100 MHz, $CDCl_3$) δ 143.2, 138.9, 137.2, 131.9, 128.5, 118.2, 117.2, 116.5, 113.4, 68.8, 65.7, 54.5, 40.3, 39.7, 38.4, 36.2, 35.3, 25.7, 21.7, 18.4. HR-CI-MS *m/z* calculated for $C_{20}H_{30}O_2$ $[M+H]^+$ 303.2324, found: 303.2328.

Diastereomer **2b**- 0.4 g (32%), $[\alpha]_D$ = -94.2° (c = 0.26, MeOH). ^{1}H NMR (400 MHz, $CDCl_3$) δ 6.38 (dd, *J* = 17.6, 10.7 Hz, 1H), 5.87 (ddt, *J* = 17.3, 10.1, 7.3 Hz, 1H), 5.28 (d, *J* = 17.6 Hz, 1H), 5.20 – 4.84 (m, 6H), 4.57 – 4.38 (m, 1H), 3.90 – 3.48 (m, 2H), 2.46 (dd, *J* = 13.7, 7.7 Hz, 1H), 2.36 – 2.20 (m, 2H), 2.15 – 1.99 (m, 1H), 1.92 (ddd, *J* = 11.8, 7.2, 4.3 Hz, 1H), 1.78 – 1.32 (m, 12H). ^{13}C NMR (100 MHz, $CDCl_3$) δ 143.1, 139.1, 137.2, 130.6, 128.7, 118.7, 117.6, 116.4, 113.2, 69.9, 65.5, 54.6, 40.4, 39.3, 38.2, 36.6, 34.7, 25.7, 21.4, 18.3.

Preparation of (*S*)-ipsdienol **1a**

A solution of compound **2a** (0.1 g; 0.33 mmol) and catalytic amount of pyridinium *p*-toluenesulfonate (approx. 0.005 g) in anhydrous methanol (2 mL) was stirred for five days. The reaction mixture was diluted with diethyl ether (5 mL) and washed with water. The organic layer was concentrated under reduced pressure and the residue was purified by column chromatography (silica gel, eluent DCM). Methanolysis afforded 0.03 g (58%) of the product. Chiral GC determined the enantiomeric excess to be 98.2%. $[\alpha]_D$ = +13.1° (c = 0.18, MeOH).

Preparation of (*R*)-ipsdienol **1b**

This compound was prepared using a procedure similar to the one used for the preparation of the enantiomer **1a**. Methanolysis of **2b** (0.1 g; 0.33 mmol) afforded 0.025 g (49%) of the title compound. Chiral GC determined the enantiomeric excess to be 98.0%. $[\alpha]_D$ = -13.2° (c = 0.19, MeOH).

Preparation of (*S*)-ipsenol **3a**

The solution of butyllithium (5 mL, 2.5 M solution in hexane; 12.5 mmol) was added dropwise to a solution of tetramethylpiperidine (1.80 g; 12.5 mmol) in dry THF (5 mL) placed in an ice bath. After 15 min of stirring the mixture was cooled to -78 °C. A solution of potassium *t*-butoxide (1.40 g; 12.5 mmol) in dry THF (8 mL) was added dropwise. Then neat isoprene (1.30 g; 18.0 mmol) was added slowly to the mixture, which turned red. The reaction mixture was stirred at -60 °C for 20 min. Afterwards the mixture was cooled to -78 °C and a solution of (+)-*B*-methoxy-diisopinocampheylborane (3.95 g; 12.5 mmol) in THF (3 mL) was added. The reaction mixture was allowed to stir for 20 min at -78 °C and then neat boron trifluoride etherate (2.0 mL; 16.0 mmol) was added dropwise in 5 min. Isovaleraldehyde (1.1 g; 12.5 mmol) in THF (3 mL) was added dropwise to a rapidly stirred mixture of Ipc_2BIpn, maintained at -78 °C. Neat acetaldehyde (1.1 mL) was added in one portion and the mixture was allowed to warm to 0 °C. THF was evaporated on the rotavap and the obtained residue was diluted with diethyl ether (20 mL). Finally, diethanolamine (1.3 mL; 14.0 mmol) was added. The mixture was stirred for 1 hour. The formed suspension was passed through a plug of Celite. The filtrate was concentrated under reduced pressure and chromatographed (silica gel; gradient elution started with straight pentane, then was used mixture of pentane:DCM/ 9:1 to 1:1). The *one pot* reaction afforded 0.7 g (36%) of colorless liquid. $[\alpha]_D$ = -13.0° (c = 0.29, MeOH). Chiral GC technique determined enantiomeric excess as 97.8%. HR-MS-TOF-EI+ *m/z* calculated for $C_{10}H_{18}O$ $[M]^+$ 154.1358, found: 154.1362.

Preparation of (*R*)-ipsenol **3b**

This compound was prepared using a procedure similar to the one used for the preparation of the enantiomer **3a**. The *one pot* reaction using of (-)-*B*-methoxy-diisopinocampheylborane (3.95 g; 12.5 mmol) afforded 0.8 g (41%) of colorless liquid. $[\alpha]_D$ = +10.4° (c = 0.27, MeOH). Chiral GC determined the enantiomeric excess to be 98.8%.

Preparation of isoprenylpinacolboronic ester **4**

The solution of butyllithium (10 mL, 2.5 M solution in hexane; 25.0 mmol) was added dropwise to a solution of tetramethylpiperidine (3.54 g; 25.0 mmol) in dry THF (10 mL) placed in an ice bath. After 15 min of stirring the mixture was cooled to -78 °C. A solution of potassium *t*-

butoxide (2.80 g; 25.0 mmol) in dry THF (15 mL) was added dropwise. Then neat isoprene (2.60 g; 37.0 mmol) was added slowly to the mixture, which turned red. The reaction mixture was stirred at -60 °C for 20 min. Afterwards the mixture was cooled to -78 °C and 2-isopropoxy-4,4,5,5-tetramethyldioxaborolane (4.6 g; 25.0 mmol) was added. After 20 min the reaction was quenched by addition of boron trifluoride etherate (4.0 mL; 32.0 mmol). The reaction mixture was concentrated down to thick oil that was loaded onto silica gel. Column chromatography (eluent cyclohexane:Et_2O/ 10:1) afforded 1.86 g (38%) of colorless oil. ^{1}H NMR (400 MHz, $CDCl_3$) δ 6.47 (ddd, *J* = 17.4, 10.5, 0.6 Hz, 1H), 5.20 (dd, *J* = 17.4, 0.8 Hz, 1H), 5.11 – 4.99 (m, 3H), 1.89 (s, 2H), 1.26 (s, 12H). ^{13}C NMR (100 MHz, $CDCl_3$) δ 143.1, 139.8, 116.6, 113.7, 83.4, 24.7.

Racemic ipsenol **3**

A mixture of boronate **4** (0.65 g; 3.35 mmol) and isovaleraldehyde (0.29 g; 3.35 mmol) in dry toluene (4 mL) was stirred for two days at room temperature. Then the reaction was quenched with a saturated solution of ammonium chloride (2 mL). The product was extracted with diethyl ether. Combined organic layers were washed with brine and gently concentrated (the ether was evaporated). Column chromatography (eluent cyclohexane: Et_2O/ 2:1) afforded 0.31 g (60%) of the desired product as colorless liquid. ^{1}H NMR (400 MHz, $CDCl_3$) δ 6.42 (dd, *J* = 17.6, 10.7 Hz, 1H), 5.31 – 4.96 (m, 4H), 3.85 (tt, *J* = 8.7, 4.0 Hz, 1H), 2.52 (ddd, *J* = 13.9, 3.6, 1.1 Hz, 1H), 2.23 (dd, *J* = 13.9, 9.1 Hz, 1H), 1.85 (dddd, *J* = 13.2, 12.1, 8.6, 6.6 Hz, 1H), 1.48 (ddd, *J* = 13.7, 8.5, 5.3 Hz, 1H), 1.30 (ddd, *J* = 13.6, 8.6, 4.2 Hz, 1H), 0.97 (d, *J* = 6.7 Hz, 3H), 0.95 (d, *J* = 6.6 Hz, 3H). ^{13}C NMR (100 MHz, $CDCl_3$) δ 143.2, 138.5, 118.5, 114.2, 67.6, 53.4, 46.5, 40.6, 24.7, 23.5, 22.1.

Gas chromatographic (GC) analysis with chiral column

All analyses were carried out using gas chromatography coupled with mass spectrometric detection (GC-MS) using a TRACE 1310 GC with an ISQ LT mass spectrometer with electron ionization (70 eV), equipped with a quadrupole mass analyzer (Thermo Scientific, Waltham, MA, USA) and a Cyclodex-B column (30 m, i.d. 0.25 mm, 0.25 μm film thickness, Agilent, Santa Clara, CA, USA). The temperature program was 90 °C (80 min) to 200 °C (10 min) at 15

°C/min. The inlet temperature was set to 200 °C and the samples were introduced in split mode (1:30). Helium was used as a carrier gas, set to a constant flow at 1 ml/min.

(*S*)-ipsdienol **1a**

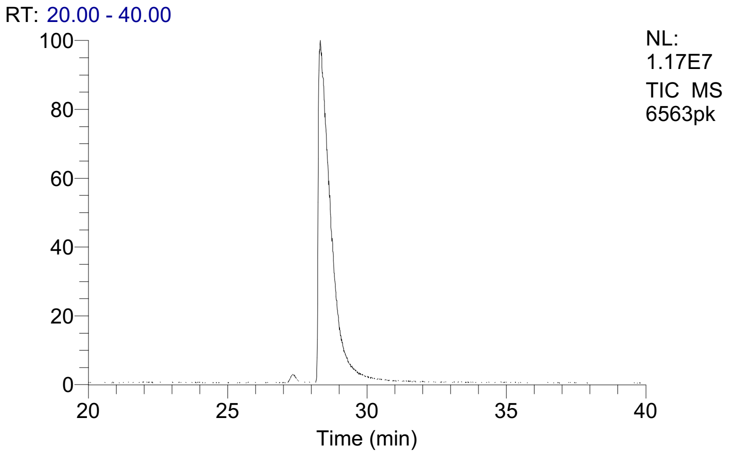

% Area
0.9
99.1

(*R*)-ipsdienol **1b**

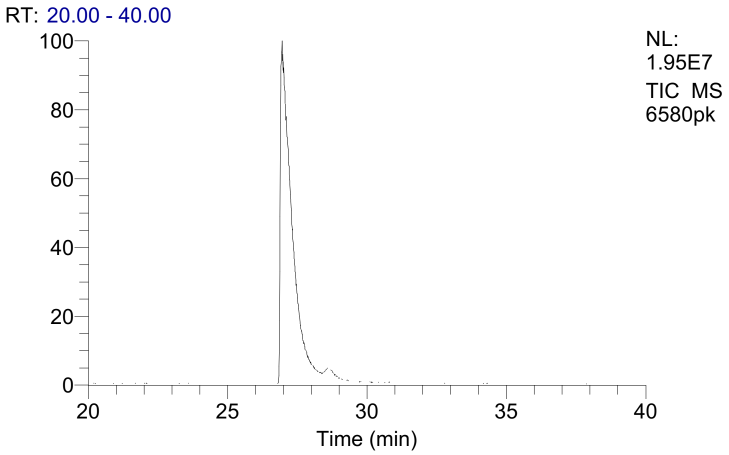

% Area
99.0
1.0

(*S*)-ipsenol **3a**

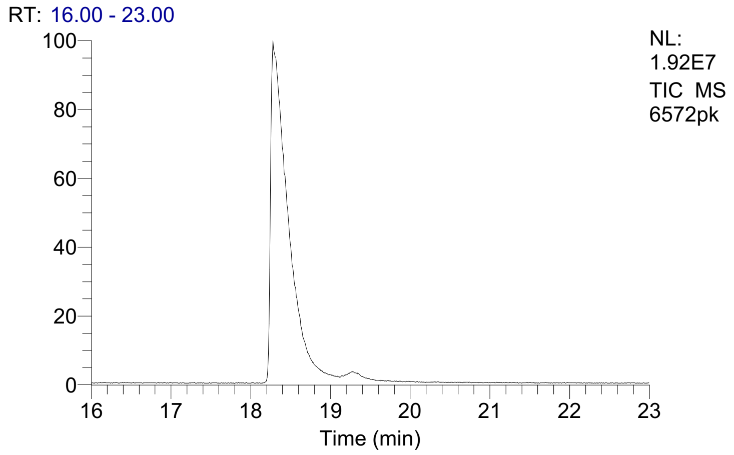

% Area
98.9
1.1

(*R*)-ipsenol **3b**

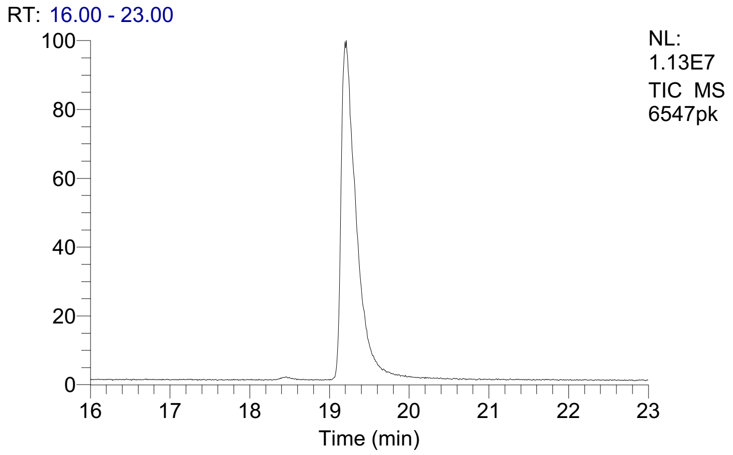

% Area
0.6
99.4

Copies of NMR spectra

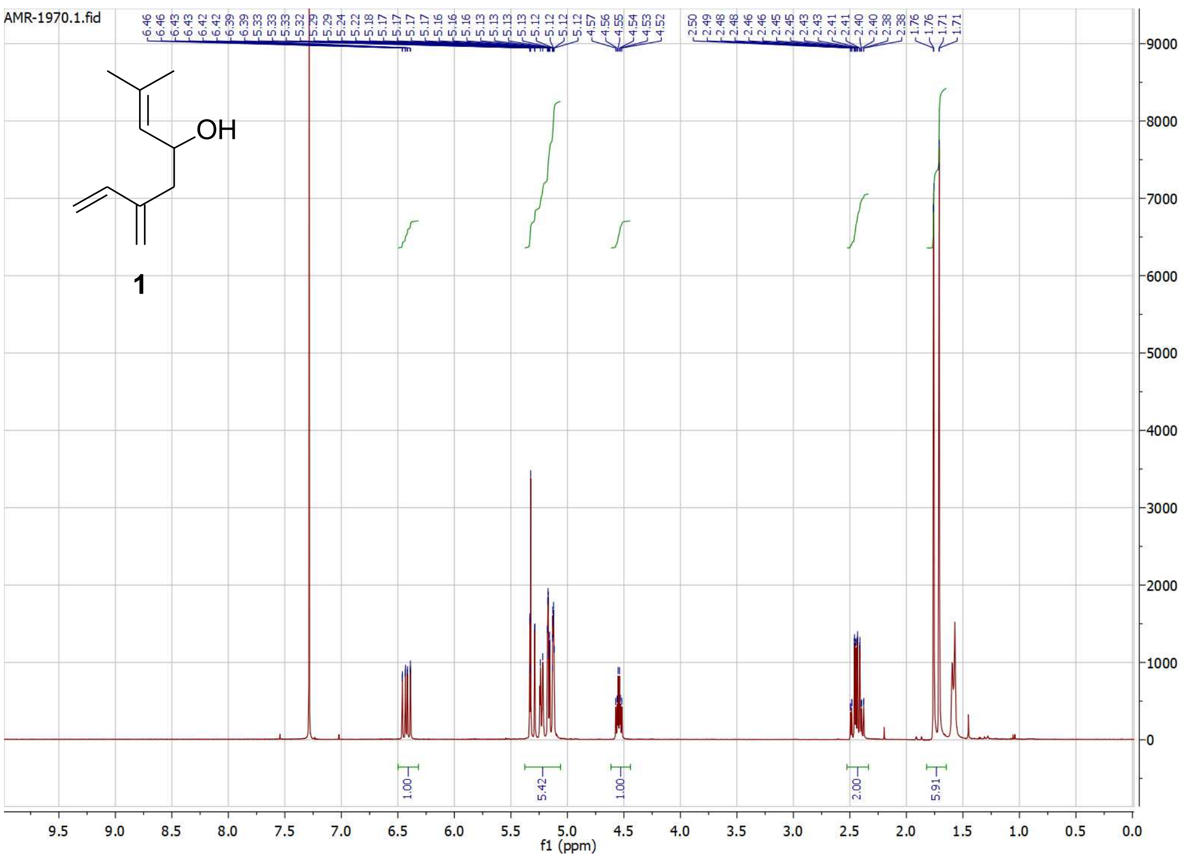

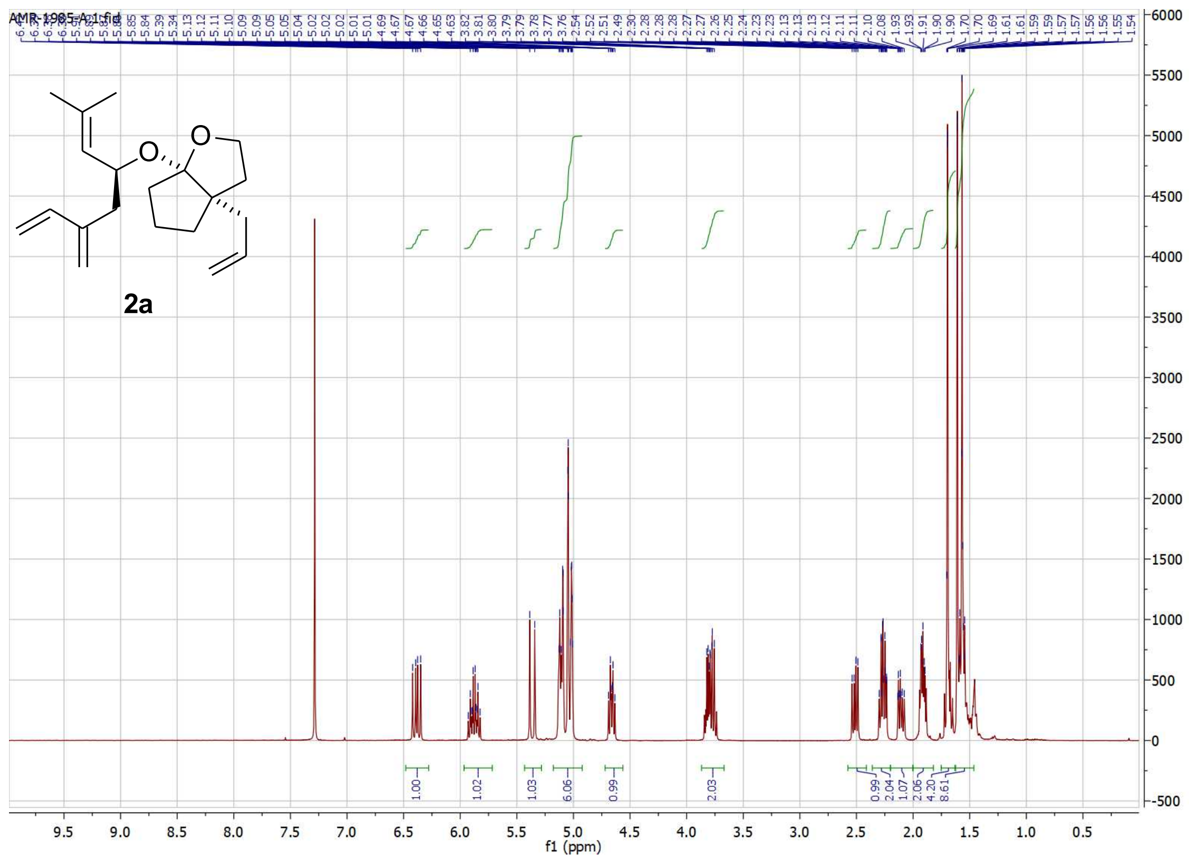

AMR-1985-A.1.fid
2a
f1 (ppm)

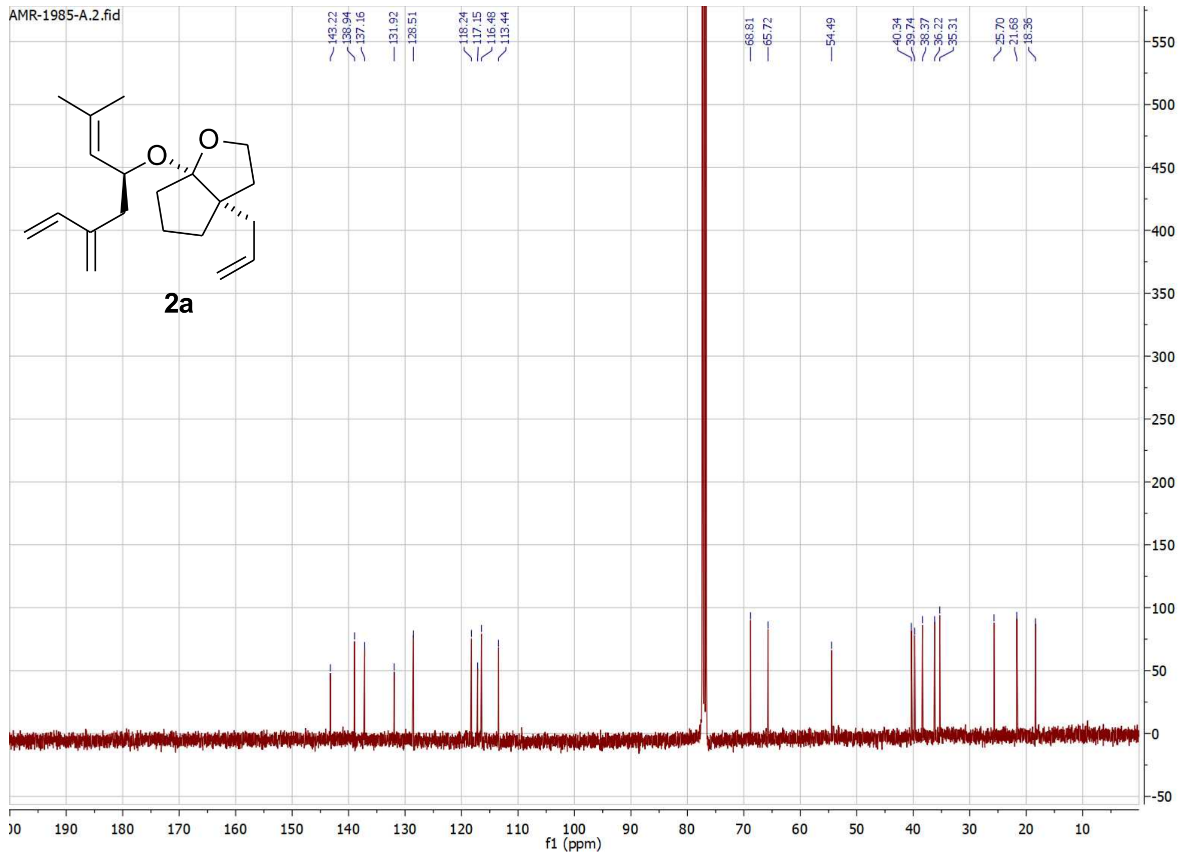

AMR-1985-A.2.fid
143.22
138.94
137.16
131.92
128.51
118.24
117.15
116.48
113.44
68.81
65.72
54.49
40.34
39.74
38.37
36.22
35.31
25.70
21.68
18.36
2a
f1 (ppm)

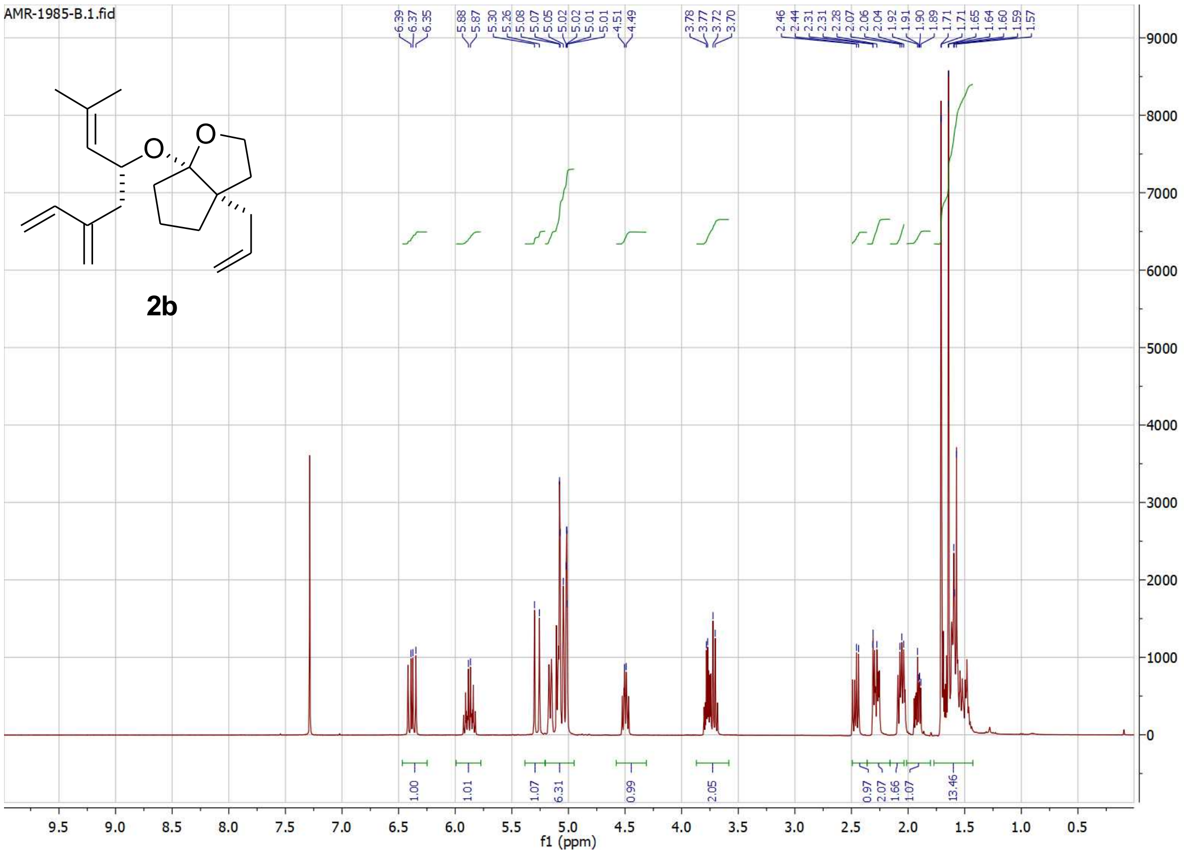
AMR-1985-B.1.fid
2b
6.39 6.37 6.35 5.88 5.87 5.30 5.26 5.08 5.07 5.05 5.02 5.02 5.01 5.01 4.51 4.49 3.78 3.77 3.72 3.70 2.46 2.44 2.31 2.31 2.28 2.07 2.06 2.04 1.92 1.91 1.90 1.89 1.71 1.71 1.65 1.64 1.60 1.59 1.57
1.00 1.01 1.07 6.31 0.99 2.05 0.97 2.07 1.66 1.07 13.46
f1 (ppm)

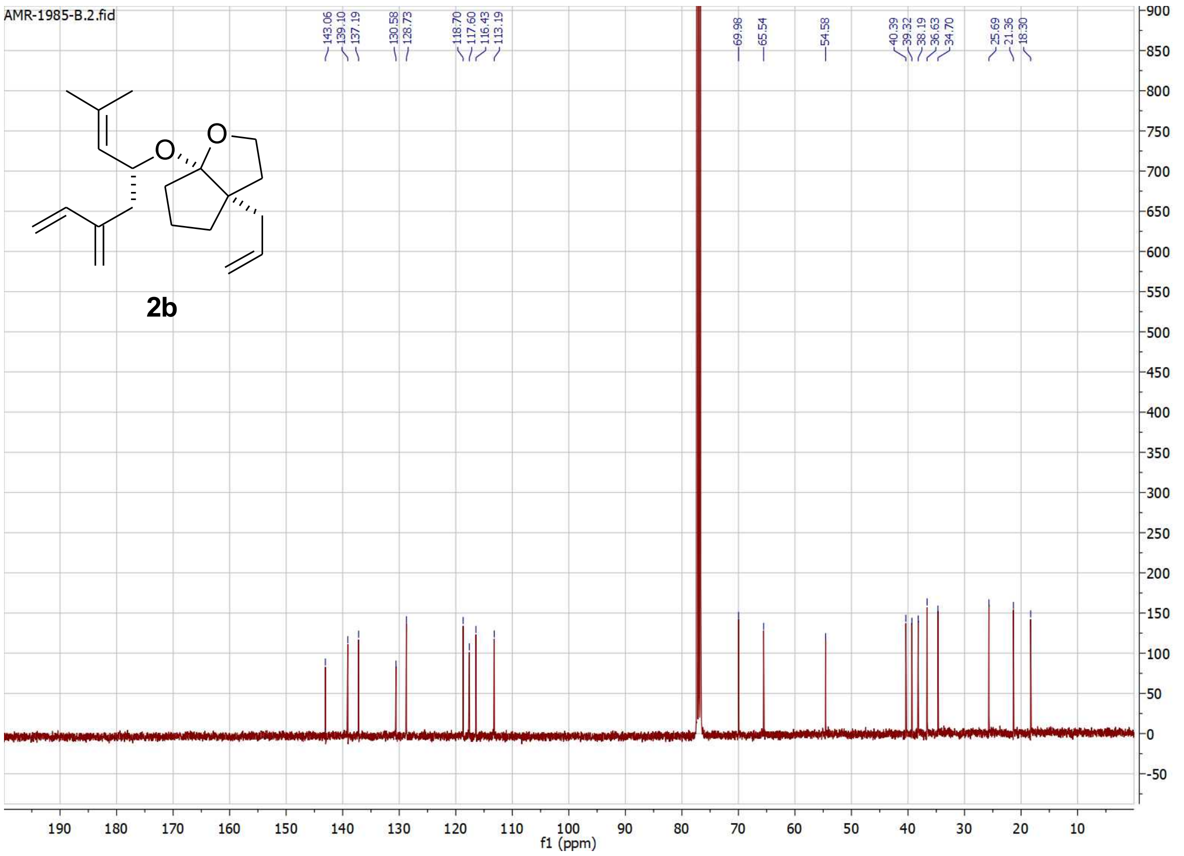
AMR-1985-B.2.fid
2b
143.06 139.10 137.19 130.58 128.73 118.70 117.60 116.43 113.19 69.98 65.54 54.58 40.39 39.32 38.19 36.63 34.70 25.69 21.36 18.30
f1 (ppm)

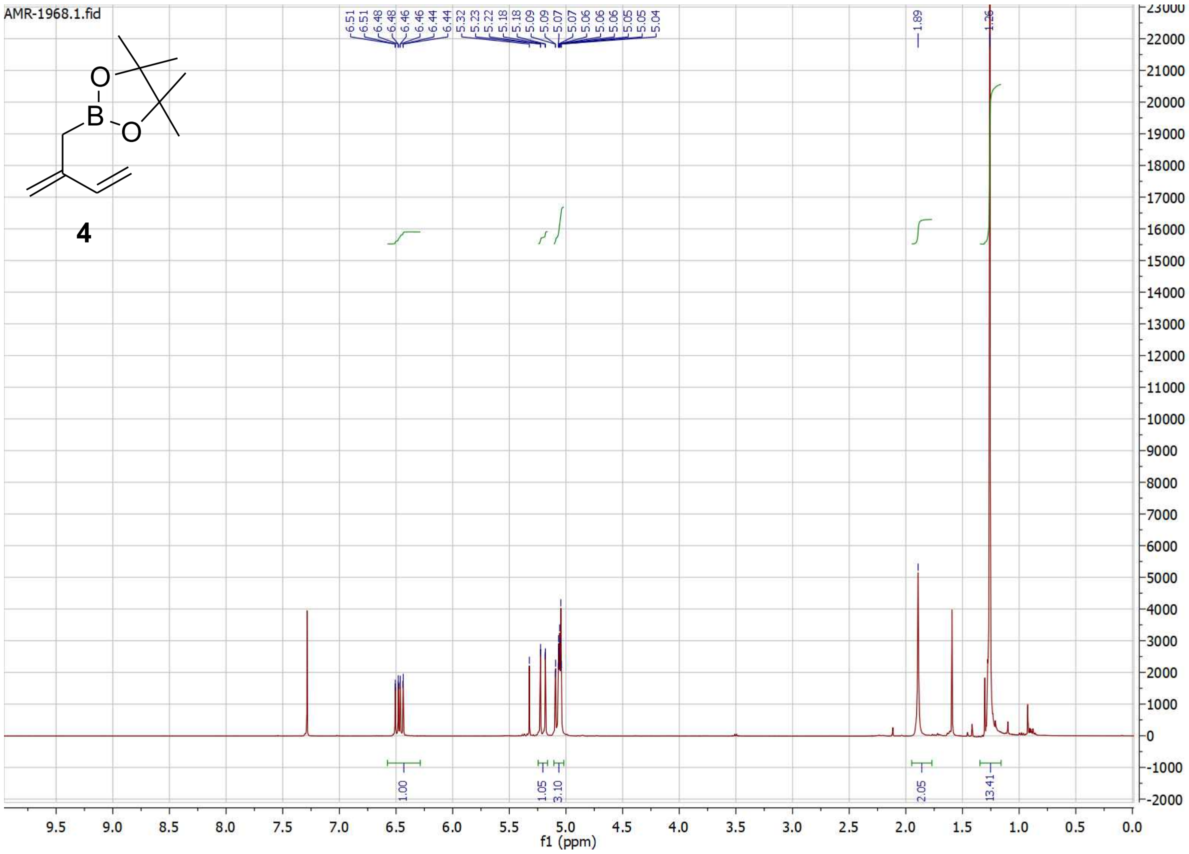
AMR-1968.1.fid
6.51
6.51
6.48
6.48
6.46
6.46
6.44
6.44
5.32
5.23
5.22
5.18
5.09
5.09
5.07
5.06
5.06
5.06
5.05
5.05
5.04
1.89
1.26
4
1.00
1.05
3.10
2.05
13.41
f1 (ppm)

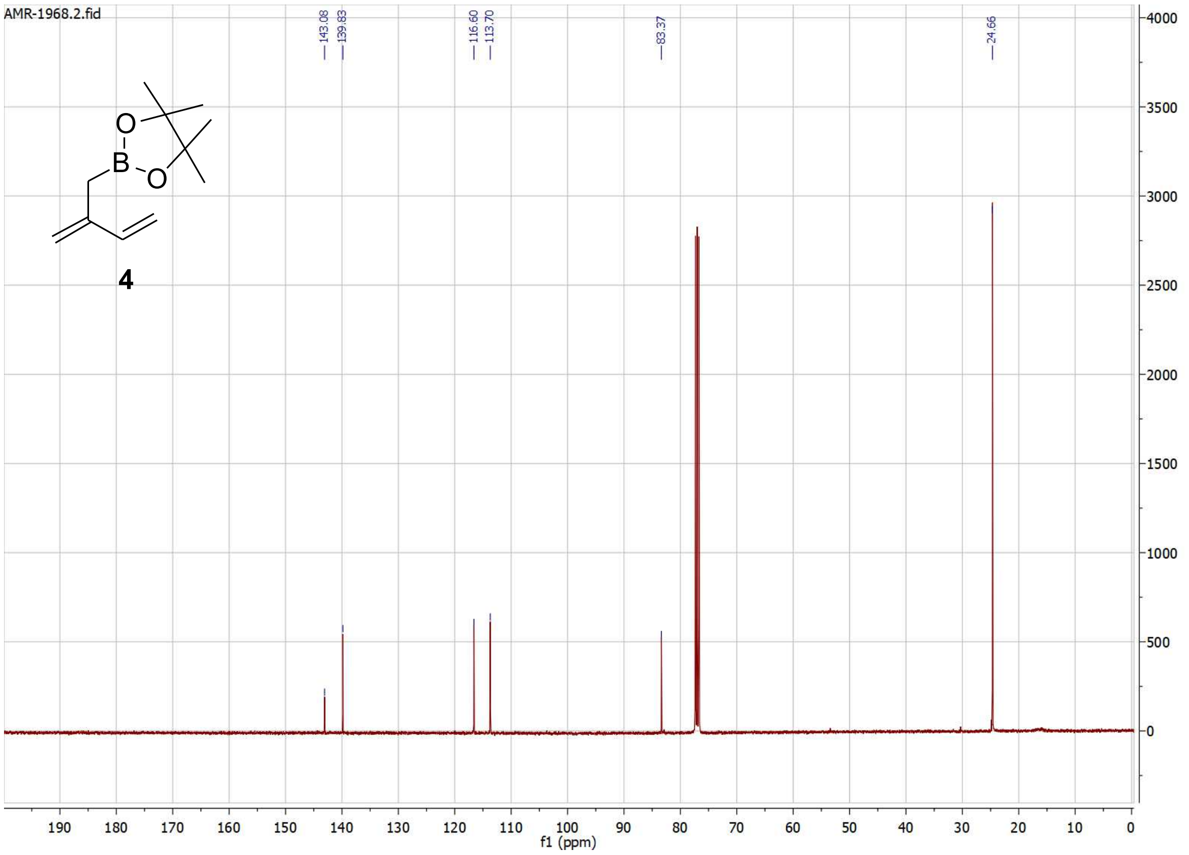
AMR-1968.2.fid
143.08
139.83
116.60
113.70
83.37
24.66
4
f1 (ppm)

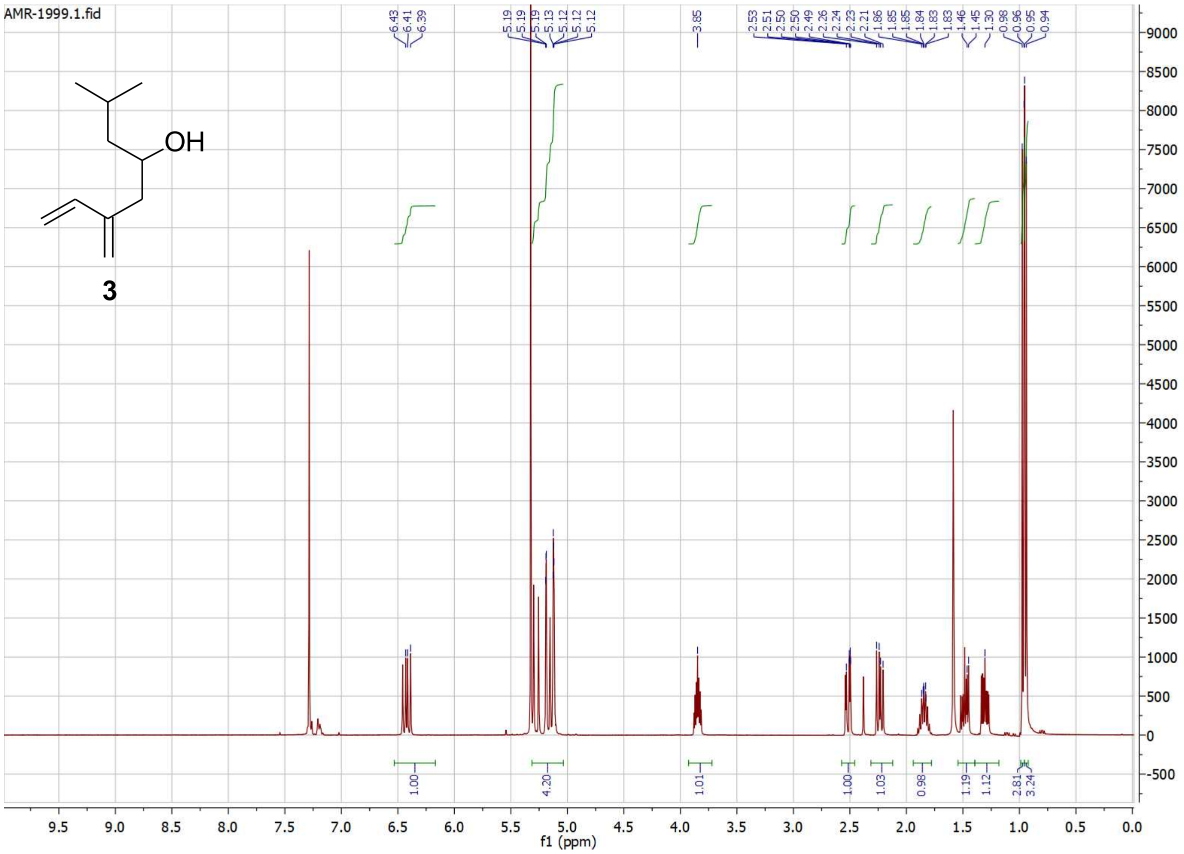
AMR-1999.1.fid
OH
3
f1 (ppm)

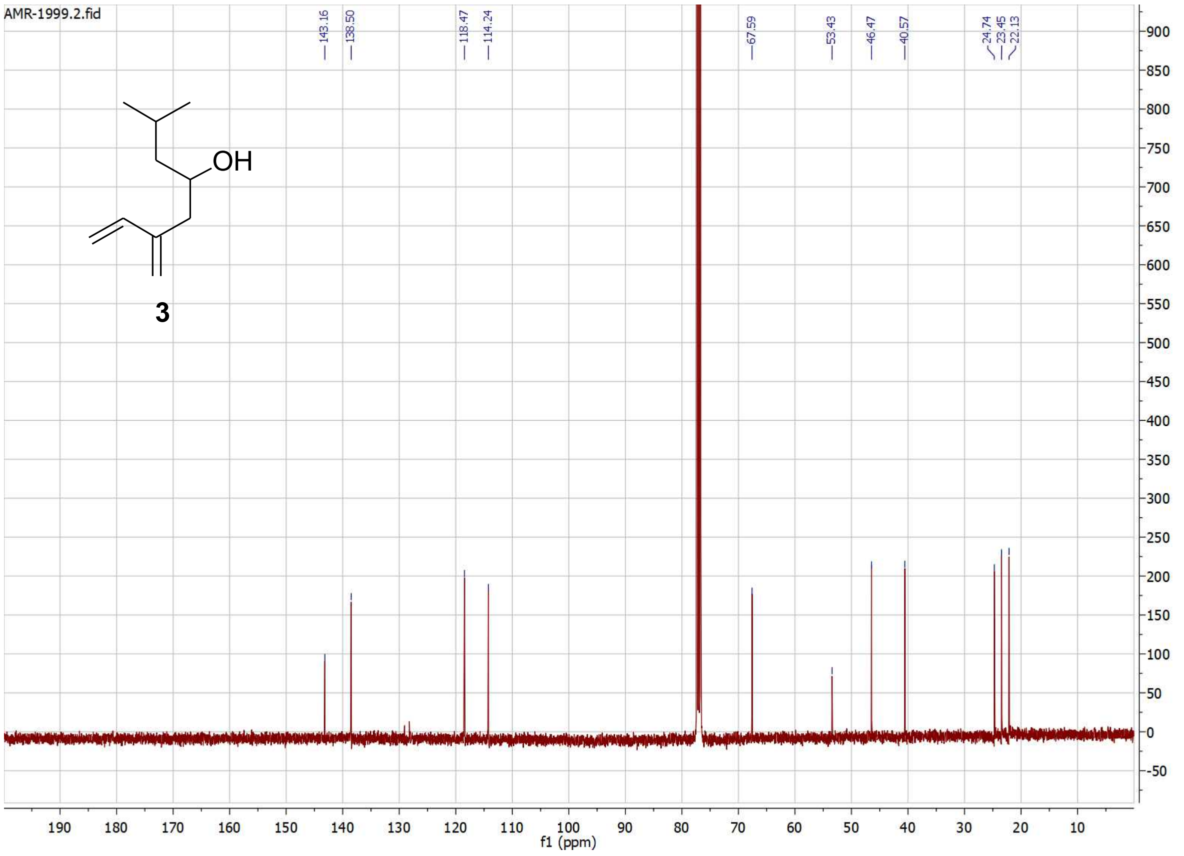
AMR-1999.2.fid
OH
3
143.16
138.50
118.47
114.24
67.59
53.43
46.47
40.57
24.74
23.45
22.13
f1 (ppm)

References

1. Klusener P.A.A.; Hommes H.H.; Verkuijsse H.D.; Brandsma L. *J. Chem. Soc. Chem. Commun.* **1985**, 1677.

2. Brown H.C.; Randad R.S. *Tetrahedron* **1990**, *46*, 4463.

3. Nemoto H. *Tetrahedron Lette*rs **1994**, *35*, 7785.

4. Nemoto H.; Zhong W.; Kawamura T.; Kamiya M.; Nakano Y.; Sakamoto K. *Synlett* **2007**, *15*, 2343.

5. Erver F.; Hilt G. *Org. Chem*. **2011**, *13*, 5700.

List of additional supplementary files

Supplementary Table 1, Additional file 1

Supplementary Data 1, Additional file 3

Supplementary Data 2, Additional file 4
